# Immunogenicity and safety of NVSI-06-07 as a heterologous booster after priming with BBIBP-CorV: a phase 2 trial

**DOI:** 10.1038/s41392-022-00984-2

**Published:** 2022-06-06

**Authors:** Nawal Al Kaabi, Yun Kai Yang, Jing Zhang, Ke Xu, Yu Liang, Yun Kang, Ji Guo Su, Tian Yang, Salah Hussein, Mohamed Saif ElDein, Shuai Shao, Sen Sen Yang, Wenwen Lei, Xue Jun Gao, Zhiwei Jiang, Hui Wang, Meng Li, Hanadi Mekki Mekki, Walid Zaher, Sally Mahmoud, Xue Zhang, Chang Qu, Dan Ying Liu, Jing Zhang, Mengjie Yang, Islam Eltantawy, Peng Xiao, Zhao Nian Wang, Jin Liang Yin, Xiao Yan Mao, Jin Zhang, Ning Liu, Fu Jie Shen, Liang Qu, Yun Tao Zhang, Xiao Ming Yang, Guizhen Wu, Qi Ming Li

**Affiliations:** 1 Sheikh Khalifa Medical City, SEHA, Abu Dhabi, United Arab Emirates; 2 College of Medicine and Health Sciences, Khalifa University, Abu Dhabi, United Arab Emirates; 3 China National Biotec Group Company Limited, Beijing, China; 4 The Sixth Laboratory, National Vaccine and Serum Institute (NVSI), Beijing, China; 5 National Engineering Center for New Vaccine Research, Beijing, China; 6 National Institute for Viral Disease Control and Prevention, Chinese Center for Disease Control and Prevention (China CDC), Beijing, China; 7 Lanzhou Institute of Biological Products Company Limited, Lanzhou, China; 8 Beijing Key Tech Statistical Consulting Co., Ltd, Beijing, China; 9 Beijing Institute of Biological Products Company Limited, Beijing, China; 10 Union 71, Abu Dhabi, United Arab Emirates; 11 G42 Healthcare, Abu Dhabi, United Arab Emirates

**Keywords:** Clinical trials, Vaccines

## Abstract

The increased coronavirus disease 2019 (COVID-19) breakthrough cases pose the need of booster vaccination. We conducted a randomised, double-blinded, controlled, phase 2 trial to assess the immunogenicity and safety of the heterologous prime-boost vaccination with an inactivated COVID-19 vaccine (BBIBP-CorV) followed by a recombinant protein-based vaccine (NVSI-06-07), using homologous boost with BBIBP-CorV as control. Three groups of healthy adults (600 individuals per group) who had completed two-dose BBIBP-CorV vaccinations 1–3 months, 4–6 months and ≥6 months earlier, respectively, were randomly assigned in a 1:1 ratio to receive either NVSI-06-07 or BBIBP-CorV boost. Immunogenicity assays showed that in NVSI-06-07 groups, neutralizing antibody geometric mean titers (GMTs) against the prototype SARS-CoV-2 increased by 21.01–63.85 folds on day 28 after vaccination, whereas only 4.20–16.78 folds of increases were observed in control groups. For Omicron variant, the neutralizing antibody GMT elicited by homologous boost was 37.91 on day 14, however, a significantly higher neutralizing GMT of 292.53 was induced by heterologous booster. Similar results were obtained for other SARS-CoV-2 variants of concerns (VOCs), including Alpha, Beta and Delta. Both heterologous and homologous boosters have a good safety profile. Local and systemic adverse reactions were absent, mild or moderate in most participants, and the overall safety was quite similar between two booster schemes. Our findings indicated that NVSI-06-07 is safe and immunogenic as a heterologous booster in BBIBP-CorV recipients and was immunogenically superior to the homologous booster against not only SARS-CoV-2 prototype strain but also VOCs, including Omicron.

## Introduction

The epidemic of coronavirus disease 2019 (COVID-19), caused by severe acute respiratory syndrome coronavirus 2 (SARS-CoV-2), has stimulated global efforts to develop safe and effective vaccines against the rapid spread of the virus. So far, great progress has been achieved, and a total of ten vaccines have been approved by the world health organization (WHO) for emergency use, including three inactivated, two mRNA-based, three viral vector-based and two recombinant nanoparticle protein-based vaccines (https://www.who.int/teams/regulation-prequalification/eul/covid-19). These COVID-19 vaccines have shown to offer effective protections against severe disease, hospitalization and death.^1^ According to the published data from clinical trials, the efficacy of several leading vaccines such as BNT162b2, ChAdOx1, Ad26.COV2.S, mRNA-1273, BBIBP-CorV, CoronaVac and NVX-CoV2373 were reported to be 95.0%, 70.4%, 67%, 94.1%, 78.1%, 51.0–83.5% and 90.4%, respectively.^2,3^ Among these vaccines, the inactivated vaccine BBIBP-CorV produced by Sinopharm has been used in large-scale populations worldwide, and many studies have demonstrated the effectiveness of this vaccine against the wild type SARS-CoV-2 and its variants.^4–8^ However, due to the waning of neutralization titer over time in vaccinated individuals and emergence of SARS-CoV-2 variants such as Omicron and Delta, breakthrough infection cases continuously increase,^9,10^ which raises the urgent need of new strategies to cope with this problem.

Booster vaccination may be an effective way to improve waning immunity and broaden protective immune responses against SARS-CoV-2. The clinical trials in adults who have received the two-dose primary vaccination series with mRNA-1273 or BNT162b2 vaccines showed that a booster injection of the same vaccine, six to eight months later, yielded 3.8- to 7-fold higher neutralizing antibody titers against the wild-type virus compared to the peak value after the primary series.^11–13^ Besides the homologous boosting, heterologous booster strategy has also attracted great concerns, and multiple clinical trials and cohort studies have shown that the immune response elicited by heterologous prime-booster vaccination was significantly greater than that induced by homologous counterparts.^14–21^ Currently, several clinical trials have been conducted to evaluate the safety, immunogenicity and efficacy of a heterologous booster dose of recombinant subunit vaccines, such as V-01 (NCT05096832), ZF2001 (NCT05205096, NCT05205083) and SCB-2019 (NCT05087368), following two-dose inactivated vaccines. Some preliminary study results have demonstrated that the heterologous booster of recombinant protein subunit vaccines distinctly improved the neutralizing antibody level^17–21^ and protective efficacy (https://en.livzon.com.cn/companyfile/1029.html) against various SARS-CoV-2 strains, including the Omicron variant, which was immunogenically superior to the homologous booster of inactivated vaccines.^17–21^

Based on structural and computational analysis of spike receptor-binding domain (RBD) of SARS-CoV-2, we have designed a recombinant COVID-19 vaccine (CHO cells), named NVSI-06-07, that uses a homologous trimeric form of RBD (homo-tri-RBD) as the antigen. In homo-tri-RBD, three RBDs, derived from the prototype SARS-CoV-2 strain, were connected end-to-end into a single molecule by using their own long loops at the N- and C-terminus without introducing any exogenous linker, which were then co-assembled into a trimeric structure.^22^ The safety and immunogenicity of this vaccine have been evaluated in the phase 1/2 clinical trial conducted in China. The interim analysis results showed that the immunogenicity of NVSI-06-07 was comparable to other recombinant protein-based COVID-19 vaccines, and no vaccine-related serious adverse events were reported in the trial (ClinicalTrials.gov number: NCT04869592, data not yet published). We sought to know whether the use of NVSI-06-07 as a heterologous booster vaccination can effectively improve the immune responses in the inactivated vaccine recipients.

Here, we report the immunogenicity and safety of heterologous booster vaccination with NVSI-06-07 at pre-specified time intervals in individuals who have previously received two doses of the inactivated vaccine BBIBP-CorV, which were then compared to those of homologous boosting strategy with a third dose of BBIBP-CorV. Moreover, as an exploratory study, the live-virus neutralization activities of the vaccinated sera were also evaluated against Omicron and other SARS-CoV-2 variants of concern (VOCs).

## Results

### Study participants

**Fig. 1:**
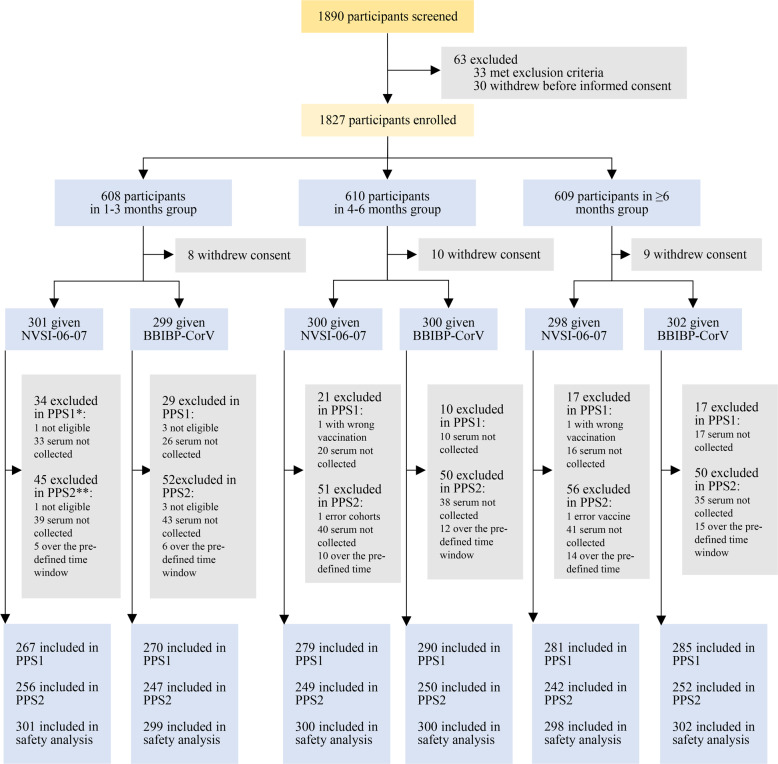
Trial profile. *PPS1: per-protocol analysis of immunogenicity on day 14 post booster vaccination; **PPS2: per-protocol analysis of immunogenicity on day 28 post booster vaccination; In PPS2, the sera from all the participants were used to evaluate the neutralizing antibody titers, and 255 participants in the 1–3 months group receiving NVSI-06-07 boost, 241 in the ≥6 months group receiving NVSI-06-07 and 251 in the ≥6 months group receiving BBIBP-CorV were used to detect the RBD-binging IgG concentrations

Healthy adults aged ≥18 yrs who received a full regimen (two doses) of BBIBP-CorV 1–3 months, 4–6 months and ≥6 months (maximum: 12.7 months, median: 7.3 months) ago, respectively, were recruited as shown in Fig. 1. For these three groups with different boosting intervals, a total of 1800 participants, with 600 of each group, form the United Arab Emirates (UAE) took part in the trial. For each group, participants were randomly assigned to receive either a heterologous booster vaccination with NVSI-06–07 or a homologous booster with a third dose of BBIBP-CorV (Fig. 1). Demographic characteristics were similar between the heterologous and homologous boosting groups. The participants in the two groups exhibited balanced distributions in age, sex, height and body weight (Table 1). The nationality of participants was provided in Supplementary Table 1. All the 1800 participants receiving booster vaccination were included in safety set (SS) for safety analysis. A total of 1672 participants completed the follow-up visit on day 14, and these individuals were included in per-protocol set 1 (PPS1) for day 14 immunogenicity analysis. A total of 1496 participants completed day 28 visit, which were included in per-protocol set 2 (PPS2) for day 28 immunogenicity analysis (Fig. 1).

**Table 1:** Demographic characteristics of the participants (FAS)

	1–3 months			4–6 months			≥6 months		
	NVSI-06-07 (*N* = 301)	BBIBP-CorV (*N* = 299)	*P* value	NVSI-06-07 (*N* = 300)	BBIBP-CorV (*N* = 300)	*P* value	NVSI-06-07 (*N* = 298)	BBIBP-CorV (*N* = 302)	*P* value
Age (yrs)									
Mean (SD)	33.30 (8.88)	33.43 (9.38)	0.8642	34.10 (7.89)	34.53 (8.59)	0.5159	35.48 (9.53)	36.12 (9.27)	0.4036
Median	31.88	32.50		33.37	33.72		34.08	34.72	
Min, Max	19.2, 65.4	18.0, 70.8		20.6, 61.8	18.9, 63.7		18.4, 63.7	18.2, 69.7	
Age group, *n*(%)			0.5519			0.3157			0.9830
18–59 yrs	296 (98.34)	292 (97.66)		299 (99.67)	297 (99.00)		293 (98.32)	297 (98.34)	
≥60 yrs	5 (1.66)	7 (2.34)		1 (0.33)	3 (1.00)		5 (1.68)	5 (1.66)	
Sex, *n*(%)			0.6146			0.5026			0.3923
Male	261 (86.71)	255 (85.28)		283 (94.33)	279 (93.00)		258 (86.58)	254 (84.11)	
Female	40 (13.29)	44 (14.72)		17 (5.67)	21 (7.00)		40 (13.42)	48 (15.89)	
Height (cm)									
Mean (SD)	167.52 (7.87)	167.61 (8.99)	0.8938	169.93 (7.97)	169.17 (7.79)	0.2389	170.51 (8.25)	169.47 (8.89)	0.1400
Median	168.00	168.00		170.00	169.00		171.00	171.00	
Min, Max	147.00, 190.00	125.00, 191.00		110.00, 187.00	143.00, 189.00		143.00, 190.50	141.00, 192.00	
Weight (kg)									
Mean (SD)	71.02 (14.22)	71.96 (14.17)	0.4165	76.10 (13.27)	74.11 (13.22)	0.0665	77.12 (15.56)	76.46 (14.41)	0.5894
Median	70.00	70.70		75.00	73.00		76.00	75.50	
Min, Max	42.00, 133.00	44.10, 126.00		46.80, 119.00	43.00, 128.00		43.50, 132.70	43.00, 127.00	
BMI (kg/m^2^)									
Mean (SD)	25.27 (4.56)	25.62 (4.72)	0.3562	26.37 (4.60)	25.88 (4.22)	0.1807	26.45 (4.59)	26.54 (4.22)	0.7905
Median	24.77	25.28		26.28	25.68		26.17	26.26	
Min, Max	16.53, 45.44	17.06, 49.28		17.30, 67.77	15.47, 43.03		16.14, 43.14	17.01, 41.95	

### Immunogenicity

For immunogenicity, the serologic RBD-specific IgG concentrations were detected before and after the boost vaccination by using ELISA kits to assess the antibody responses. The baseline IgG levels in the enrolled participants were initially determined, as shown in Table 2. There was no difference in the baseline IgG levels between participants assigned to heterologous and homologous boosting groups. On 14 days after boosting, notable increases were observed in IgG concentrations. In homologous BBIBP-CorV booster group, the seroconversion rates were 23.70% (95% CI,18.76%–29.24%), 25.17% (20.28%–30.58%) and 36.14% (30.56%–42.01%) for 1–3-month, 4–6-month and ≥6-month boosting-interval groups, respectively, whereas in heterologous NVSI-06–07 booster group, the seroconversion rates were 93.26% (95%CI, 89.55%–95.96%), 90.32% (86.23%–93.53%) and 85.77% (81.12%–89.63%), respectively (Table 2). Significantly higher seroconversion rates (*P* < 0.0001) were elicited by heterologous boosting than by homologous vaccination (Table 2). For participants receiving the homologous boost, IgG GMCs increased from baseline by 2.76-fold (95%CI, 2.39–3.17) in 1–3-month boosting-interval group, 2.63-fold (95% CI, 2.26–3.06) in 4–6-month group and 4.71-fold (3.77–5.89) in ≥6-month group, respectively. Notably, in participants receiving the heterologous boost with NVSI-06–07, IgG GMCs demonstrated a 43.41-fold (95%CI, 36.54–51.56), 44.68-fold (36.79–54.26) and 57.56-fold (44.72–74.07) of increases in the three groups with different boosting intervals, respectively (Table 2). IgG responses boosted by NVSI-06–07 were much higher (*P* < 0.0001) than those by BBIBP-CorV (Table 2). Similar results were observed on day 28 after the boost. Seroconversion rates were 84.23%–92.94% in different groups receiving heterologous boosting, which were significantly higher (all *P* < 0.0001) than those in participants receiving homologous boosting (17.60%–29.48%), as shown in Table 2. A similar increasing trend was observed in IgG GMCs, which were 1.97–3.57-fold in the groups receiving homologous prime-boost vaccination and 30.99–41.68-fold in the groups receiving heterologous prime-boost vaccination (all *P* < 0.0001). On day 28 after the boost, both seroconversion rates and IgG GMCs induced by the heterologous boost vaccination were significantly higher (*P* < 0.0001) than those induced by the homologous boost vaccination (Table 2).

**Table 2:** RBD-specific IgG response results (PPS)

	14 days after boosting			28 days after boosting		
	NVSI-06-07	BBIBP-CorV	*P* value	NVSI-06-07	BBIBP-CorV	*P* value
1–3 months						
*N* (missing)	267 (0)	270 (0)		255 (0)	247 (0)	
Pre-booster antibody GMC^a^ (95%CI)	110.14 (93.50, 129.74)	109.50 (94.24, 127.24)	0.9591	106.64 (90.17, 126.10)	106.30 (90.89, 124.33)	0.9785
Post-booster antibody GMC (95%CI)	4780.76 (4106.48, 5565.76)	301.71 (274.96, 331.07)		3304.37 (2870.60, 3803.68)	254.79 (231.81, 280.04)	
Post-booster adjusted antibody GMC (95%CI)	4776.34 (4255.49, 5360.94)	301.99 (269.23, 338.73)		3302.54 (2974.17, 3667.17)	254.93 (229.20, 283.55)	
Ratio of adjusted GMC between two groups (95%CI)^b^	15.82 (13.44, 18.61)		<0.0001^c^	12.95 (11.16, 15.04)		<0.0001^c^
Rate of seroconversion^d^, *n* (%)	249 (93.26)	64 (23.70)		237 (92.94)	54 (21.86)	
95%CI (%)	89.55, 95.96	18.76, 29.24		89.07, 95.76	16.87, 27.54	
Rate difference between two groups (%, 95%CI)^e^	69.55 (63.66,75.45)		<0.0001	71.08 (65.04,77.12)		<0.0001
Post-booster antibody GMC fold rise (95%CI)	43.41 (36.54, 51.56)	2.76(2.39, 3.17)	<0.0001	30.99 (26.47, 36.27)	2.40 (2.09, 2.75)	<0.0001
4–6 months						
*N* (missing)	279 (0)	290 (0)		249 (0)	250 (0)	
Pre-booster antibody GMC^a^ (95%CI)	152.16 (127.35, 181.81)	170.87 (142.36, 205.09)	0.3714	150.77 (124.60, 182.44)	185.48 (152.36, 225.80)	0.1370
Post-booster antibody GMC (95%CI)	6798.51 (5985.06, 7722.52)	448.93 (401.59, 501.84)		4925.67 (4361.44, 5562.89)	364.90 (324.38, 410.49)	
Post-booster adjusted antibody GMC (95%CI)	6901.42 (6174.88, 7713.43)	442.48 (396.75, 493.49)		5074.81 (4564.26, 5642.48)	354.22 (318.65, 393.76)	
Ratio of adjusted GMC between two groups (95%CI)^b^	15.60 (13.35, 18.23)		<0.0001^c^	14.33 (12.33, 16.64)		<0.0001^c^
Rate of seroconversion^d^, *n* (%)	252 (90.32)	73 (25.17)		223 (89.56)	44 (17.60)	
95%CI (%)	86.23, 93.53	20.28, 30.58		85.08, 93.06	13.09, 22.90	
Rate difference between two groups (%, 95%CI)^e^	65.15 (59.07,71.23)		<0.0001	71.96 (65.90,78.02)		<0.0001
Post-booster antibody GMC fold rise (95%CI)	44.68 (36.79, 54.26)	2.63 (2.26, 3.06)	<0.0001	32.67 (26.94, 39.62)	1.97 (1.69, 2.29)	<0.0001
≥6 months						
*N* (missing)	281 (0)	285 (0)		241 (0)	251 (0)	
Pre-booster antibody GMC^a^ (95%CI)	104.94 (82.03, 134.24)	125.99 (98.29, 161.50)	0.3039	114.54 (88.18, 148.78)	132.80 (102.75, 171.63)	0.4268
Post-booster antibody GMC (95%CI)	6039.76 (5238.91, 6963.03)	593.53 (519.22, 678.47)		4774.32 (4157.08, 5483.21)	473.53 (410.98, 545.60)	
Post-booster adjusted antibody GMC (95%CI)	6148.08 (5399.43, 7000.53)	583.21 (512.67, 663.47)		4846.54 (4249.18, 5527.88)	466.76 (410.31, 530.97)	
Ratio of adjusted GMC between two groups (95%CI)^b^	10.54 (8.78, 12.66)		<0.0001^c^	10.38 (8.64, 12.48)		<0.0001^c^
Rate of seroconversion^d^, *n* (%)	241 (85.77)	103 (36.14)		203 (84.23)	74 (29.48)	
95%CI (%)	81.12, 89.63	30.56, 42.01		79.01, 88.59	23.91, 35.54	
Rate difference between two groups (%, 95%CI)^e^	49.62 (42.71, 56.54)		<0.0001	54.75 (47.47, 62.03)		<0.0001
Post-booster antibody GMC fold rise (95%CI)	57.56 (44.72, 74.07)	4.71 (3.77, 5.89)	<0.0001	41.68 (32.05, 54.22)	3.57 (2.84, 4.48)	<0.0001

^a^GMC represents geometric mean concentration, and the unit of GMC is BAU/ml

^b^The ratio of adjusted GMC between two groups was calculated by “NVSI-06-07/ BBIBP-CorV”

^c^Covariance analysis with least square method was used to calculate the adjusted GMC and *P* value

^d^Seroconversion was defined as more than or equal to 4-fold rise form baseline in IgG concentration

^e^Rate difference = (NVSI-06-07) − (BBIBP-CorV). Rate difference and 95%CI were estimated by CMH method considering stratification factors

The immunogenic superiority of heterologous NVSI-06–07 booster to homologous BBIBP-CorV booster was further confirmed by neutralizing antibody response measured with live-virus neutralization assays. Before booster vaccination, most of the participants had detectable neutralizing activities against prototype SARS-CoV-2 and showed a comparable level between two boosting groups in the pre-booster neutralizing antibodies. The pre-booster neutralizing antibody GMT of participants in the group of over-6-month boosting-interval was about half of the values in the 4–6-month group, indicating wanning of neutralizing antibody responses over time (Table 3). On day 14 after the boost, the neutralizing antibody titers against prototype SARS-CoV-2 live virus were significantly improved in both the heterologous and homologous boosting recipients. In homologous boosting participants, the seroconversion rates in 1–3-month, 4–6-month and ≥6-month boosting-interval groups were 39.26% (95%CI, 33.40%–45.36%), 26.90% (21.88%–32.39%) and 52.98% (47.01%–58.89%), respectively, whereas they were 81.65% (76.47%–86.10%), 86.38% (81.79%–90.18%) and 86.83% (82.31%–90.56%) for the heterologous boost (Table 3). The seroconversion rates induced by heterologous boost were significantly higher (*P* < 0.0001) than those induced by homologous boost (Table 3). Compared with the pre-boosting baseline level, the homologous boost vaccination elicited 3.41-fold (95%CI, 2.90–4.00) higher neutralizing GMTs against prototype SARS-CoV-2 in 1–3-month boosting-interval group, 2.58-fold (95% CI, 2.21–3.00) higher in 4–6-month group and 7.36-fold (95%CI, 6.11–8.86) higher in ≥6-month group, respectively. A more remarkable improvement of neutralizing antibody responses was observed by heterologous boost vaccination against prototype live virus, in which neutralizing GMTs increased by 13.95-fold (95%CI, 12.01–16.20), 16.45-fold (14.10–19.19) and 35.86-fold (29.44–43.67) for the three groups (Table 3). On day 28 after the boost, live-virus neutralizing antibody responses were further improved in both homologous and heterologous boosting groups. By homologous boosting, seroconversion rates were further increased to 59.92% (95%CI, 53.52%–66.08%), 36.80% (30.81%–43.11%) and 81.75% (76.41%–86.31%) in the 1–3-month, 4–6-month and ≥6-month boosting-interval groups, respectively. Much higher seroconversion rates were obtained by heterologous boost, which reached at 90.63% (95%CI, 86.37%–93.90%), 89.96% (85.54%–93.40%) and 97.52% (94.68%–99.08%) in the three groups, respectively. On day 28 after the boost, the increases from baseline in neutralizing GMTs of heterologous prime-boost vaccination were also significantly higher than those of homologous vaccination. By homologous boosting, neutralizing GMTs improved by 7.08-fold (95%CI, 5.91–8.48), 4.20-fold (3.57–4.94) and 16.78-fold (13.51–20.83) in the three groups, respectively, whereas 21.01-fold (95%CI, 18.01–24.52), 23.10-fold (19.44–27.44) and 63.85-fold (52.15–78.18) of increases were obtained by heterologous boost (Table 3). Both on day 14 and 28 after the boost, neutralizing antibody levels improved by heterologous booster were much higher (*P* < 0.0001) than those by homologous booster, indicating that NVSI-06–07 is immunologically preferred as a booster choice over BBIBP-CorV (Table 3). Comparison among three groups with different prime-boosting intervals by using covariance analysis models showed that the ≥6 months groups have a significantly higher increase (*P* < 0.05) in neutralizing GMTs than the 1–3 months and 4–6 months groups both for heterologous and homologous boosts (Supplementary Table 2).

**Table 3:** Live-virus neutralizing antibody response results (PPS)

	14 days after boosting			28 days after boosting		
	NVSI-06-07	BBIBP-CorV	*P* value	NVSI-06-07	BBIBP-CorV	*P* value
1–3 months						
*N* (missing)	267 (0)	270 (0)		256 (0)	247 (0)	
Pre-booster antibody GMT^a^ (95%CI)	95.71 (81.88, 111.88)	86.93 (73.84, 102.33)	0.4018	93.44 (79.36, 110.02)	83.63 (70.42, 99.31)	0.3571
Post-booster antibody GMT (95%CI)	1335.43 (1152.56, 1547.31)	296.20 (266.22, 329.55)		1963.31 (1713.49, 2249.55)	592.12 (528.52, 663.38)	
Post-booster adjusted antibody GMT (95%CI)	1313.12 (1169.39, 1474.52)	301.17 (268.38, 337.97)		1933.53 (1722.58, 2170.32)	601.57 (534.82, 676.66)	
Ratio of adjusted GMT between two groups(95%CI)^b^	4.36 (3.70, 5.13)		<0.0001^c^	3.21(2.73, 3.79)		<0.0001^c^
Rate of seroconversion^d^, *n* (%)	218 (81.65)	106 (39.26)		232 (90.63)	148 (59.92)	
95%CI (%)	76.47, 86.10	33.40, 45.36		86.37, 93.90	53.52, 66.08	
Rate difference between two groups (%, 95%CI)^e^	42.39 (34.94, 49.84)		<0.0001	30.71 (23.63, 37.78)		<0.0001
Post-booster antibody GMT fold rise (95%CI)	13.95 (12.01, 16.20)	3.41 (2.90, 4.00)	<0.0001	21.01 (18.01, 24.52)	7.08 (5.91, 8.48)	<0.0001
4–6 months						
*N* (missing)	279 (0)	290 (0)		249 (0)	250 (0)	
Pre-booster antibody GMT^a^ (95%CI)	110.22 (93.72, 129.63)	127.18 (108.73, 148.76)	0.2121	109.41 (91.81, 130.39)	138.25 (117.12, 163.19)	0.0569
Post-booster antibody GMT (95%CI)	1812.82 (1604.36, 2048.37)	327.81 (297.72, 360.93)		2527.18 (2213.43, 2885.41)	580.70 (518.66, 650.16)	
Post-booster adjusted antibody GMT (95%CI)	1848.96 (1670.38, 2046.62)	321.64 (291.14, 355.33)		2612.33 (2332.20, 2926.09)	561.85 (501.72, 629.19)	
Ratio of adjusted GMT between two groups(95%CI)^b^	5.75 (4.99, 6.63)		<0.0001^c^	4.65 (3.96, 5.46)		<0.0001^c^
Rate of seroconversion^d^, *n* (%)	241 (86.38)	78 (26.90)		224 (89.96)	92 (36.80)	
95%CI (%)	81.79, 90.18	21.88, 32.39		85.54, 93.40	30.81, 43.11	
Rate difference between two groups (%, 95%CI)^e^	59.48 (52.98,65.98)		<0.0001	53.16 (46.11,60.21)		<0.0001
Post-booster antibody GMT fold rise (95%CI)	16.45 (14.10, 19.19)	2.58 (2.21, 3.00)	<0.0001	23.10 (19.44, 27.44)	4.20 (3.57, 4.94)	<0.0001
≥6 months						
*N* (missing)	281 (0)	285 (0)		242 (0)	252 (0)	
Pre-booster antibody GMT^a^ (95%CI)	53.17 (43.28, 65.32)	61.07 (50.26, 74.20)	0.3363	59.05 (47.80, 72.95)	64.92 (53.10, 79.38)	0.5224
Post-booster antibody GMT (95%CI)	1906.60 (1651.75, 2200.77)	449.30 (406.41, 496.72)		3770.62 (3263.18, 4356.98)	1089.23 (959.93, 1235.95)	
Post-booster adjusted antibody GMT (95%CI)	1937.97 (1728.18, 2173.23)	442.13 (394.58, 495.40)		3806.74 (3341.56, 4336.68)	1079.31(949.89, 1226.35)	
Ratio of adjusted GMT between two groups(95%CI)^b^	4.38 (3.73, 5.15)		<0.0001^c^	3.53 (2.94, 4.23)		<0.0001^c^
Rate of seroconversion^d^, *n* (%)	244 (86.83)	151 (52.98)		236 (97.52)	206 (81.75)	
95%CI (%)	82.31, 90.56	47.01, 58.89		94.68, 99.08	76.41, 86.31	
Rate difference between two groups (%, 95%CI)^e^	33.85 (26.84,40.87)		<0.0001	15.77 (10.62,20.93)		<0.0001
Post-booster antibody GMT fold rise (95%CI)	35.86 (29.44, 43.67)	7.36 (6.11, 8.86)	<0.0001	63.85 (52.15, 78.18)	16.78 (13.51, 20.83)	<0.0001

^a^GMT represent geometric mean titer

^b^The ratio of adjusted GMT between two groups was calculated by “NVSI-06-07/ BBIBP-CorV”, and the non-inferiority threshold of ratio between groups was set to 0.67

^c^Covariance analysis with least square method was used to calculate the adjusted GMT and *P* value

^d^Seroconversion was defined as more than or equal to 4-fold rise form baseline in neutralizing antibody titer

^e^Rate difference = (NVSI-06-07) − (BBIBP-CorV). Rate difference and 95%CI were estimated by CMH method considering stratification factors

In order to investigate whether the immune response elicited by booster vaccination was age-dependent, participants in each group were divided into different age subgroups and immunogenicity data was compared between these subgroups (Supplementary Tables 3–10). Statistical analysis showed that both RBD-binding IgG GMCs and neutralizing antibody GMTs induced by heterologous booster were comparable between different age subgroups (*P* > 0.05), except the IgG GMCs between 18–44 yrs and ≥45 yrs subgroups in 1–3 months group (*P* = 0.0467). These results indicated that the heterologous NVSI-06–07 booster exhibited similar immunogenicity across different age subgroups. However, it should be noted that the number of older participants was much smaller than that of younger participants in the trial. Immunogenicity of the NVSI-06-07 booster in elderly population should be further assessed in the future.

### Cross-reactive immunogenicity against main SARS-CoV-2 VOCs including Omicron

**Fig. 2:**
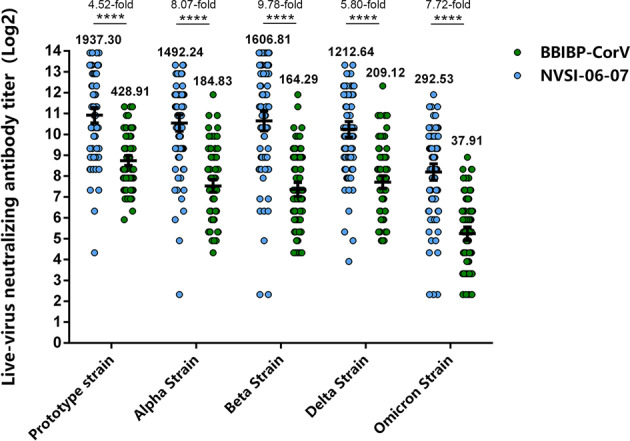
Cross-reactivity of neutralizing antibody responses against SARS-CoV-2 prototype strain and main VOCs, including Alpha, Beta, Delta and Omicron, elicited by heterologous NVSI-06-07 booster, compared with those elicited by homologous BBIBP-CorV booster. The live-virus neutralizing antibody titers were detected on day 14 post-boost. Serum samples of 192 (half boosted with homologous vaccination and the other half boosted with heterologous vaccination) participants with sequential enrollment numbers in ≥6-month boosting-interval group were tested. Both neutralizing antibody GMTs and the ratio of neutralizing GMTs between heterologous and homologous boosters are provided in the figure. Data are presented as GMT and 95% CI. ****P < 0.0001

Serum samples of 192 participants with sequential enrollment numbers in ≥6-month boosting-interval group (half boosted with homologous vaccination and the other half boosted with heterologous vaccination) were used to evaluate the neutralizing sensitivities to the Omicron variant using live-virus neutralization assays. In participants boosted with a third dose of BBIBP-CorV, neutralizing antibody GMT against Omicron was substantially reduced by 11.32 folds on day 14 post-boost compared with that against prototype SARS-CoV-2 strain, implying substantial escape of the Omicron variant from the antibody neutralization response elicited by BBIBP-CorV. By comparison, in participants receiving heterologous boost of NVSI-06-07, neutralizing antibody GMT against Omicron only declined by 6.62 folds, as shown in Fig. 2. Neutralizing antibody GMT against Omicron elicited by heterologous boost was 292.53 (95%CI, 222.81–384.07), which was significantly higher than 37.91 (95% CI, 30.35–47.35) induced by homologous boost. Heterologous prime-booster vaccination with BBIBP-CorV followed by NVSI-06–07 demonstrated much more robust neutralizing activities against Omicron compared with homologous prime-boost vaccination with three doses of BBIBP-CorV.

We also evaluated the immune response of booster vaccinations against other SARS-CoV-2 VOCs, including Alpha, Beta and Delta by using the subset of serum samples from ≥6-month boosting-interval group. On day 14 after boosting with BBIBP-CorV, the neutralizing antibody GMTs against Alpha, Beta and Delta showed 2.32, 2.61 and 2.05 folds decrease compared to that against prototype strain. All these three VOCs exhibited less sensitivities to sera neutralization, among which Beta variant showed the largest reduction in neutralization sensitivity. By comparison, the sera from the participants boosted with NVSI-06-07 showed only 1.30, 1.21 and 1.60 folds reduction in neutralization of the Alpha, Beta and Delta variants, respectively. By heterologous booster vaccination, neutralizing antibody GMTs against these three VOCs were 1492.24 (95% CI,1137.05–1958.38), 1606.81 (1152.66–2239.90) and 1212.64 (935.92–1571.18), respectively, whereas the GMTs by homologous boost were 184.83 (148.96–229.36), 164.29 (130.41–206.97) and 209.12 (168.94–258.85), respectively (Fig. 2). The heterologous boost elicited much higher neutralizing activities against the tested VOCs.

### Safety

**Fig. 3:**
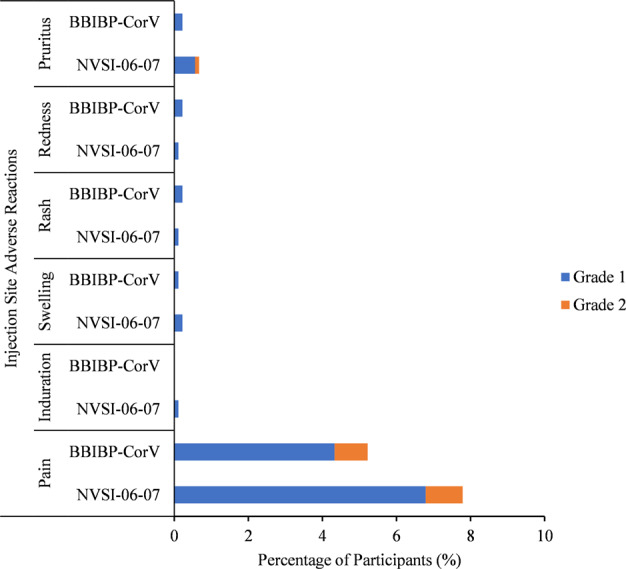
Injection site adverse reactions reported within 7 days after injection of NVSI-06-07 or BBIBP-CorV. Adverse reactions are graded according to the scale issued by the China National Medical Products Administration (NMPA). Grade 1 is mild and grade 2 is moderate

**Fig. 4:**
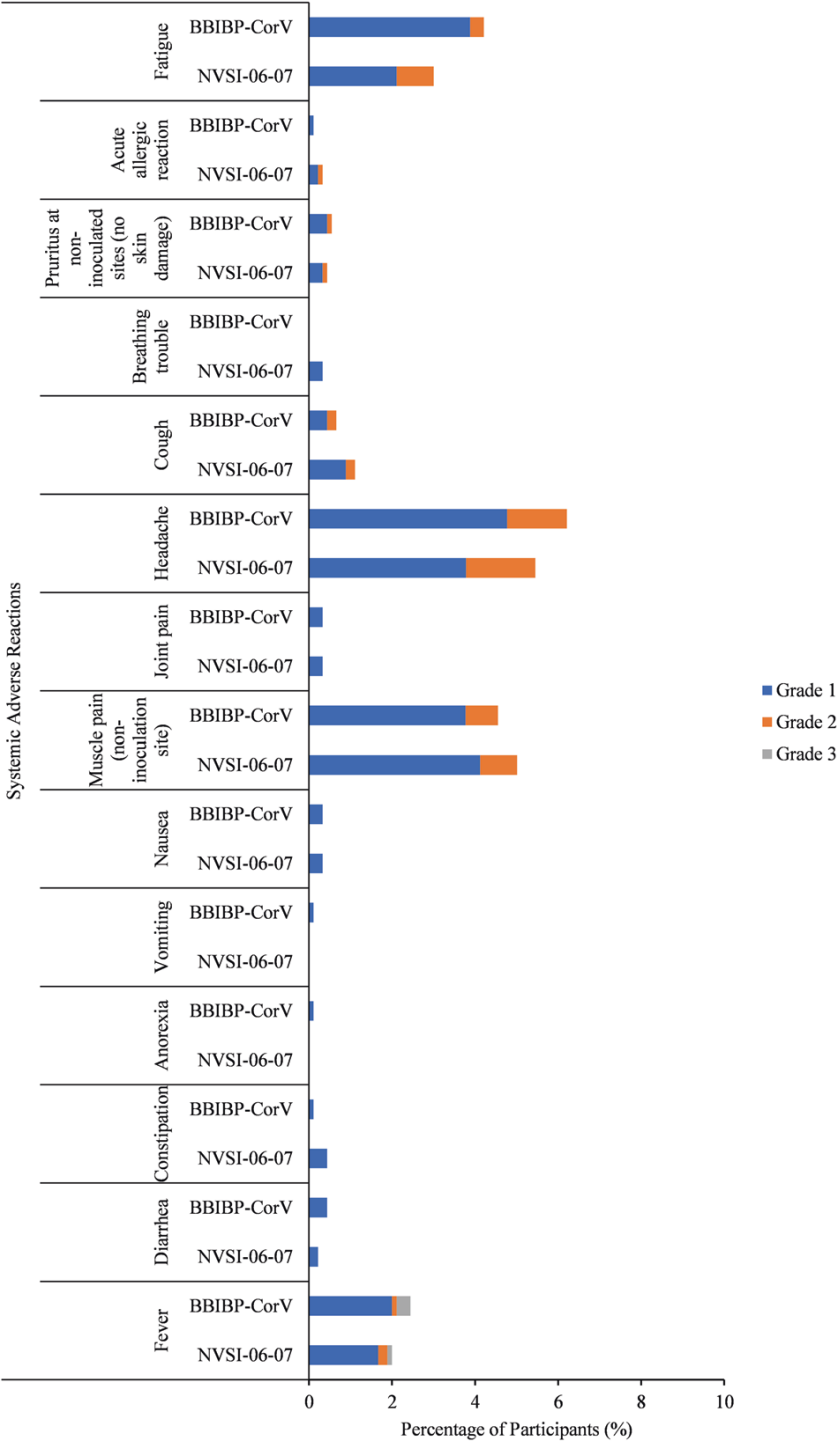
Systemic adverse reactions reported within 7 days after injection of NVSI-06-07 or BBIBP-CorV. Adverse reactions are graded according to the scale issued by the China National Medical Products Administration (NMPA). Grade 1 is mild, grade 2 is moderate, and grade 3 is severe

For safety analysis, four participants reported serious adverse events (SAEs) within 30 days after the boost, two of whom occurred in homologous booster group, and the other two was reported in heterologous booster group. None of these SAEs was related to the tested vaccines as assessed by the investigator (Supplementary Table 11). Besides, no adverse event of special interest (AESI) was reported. The overall occurrence of adverse reactions was low in both the heterologous and homologous booster vaccinations. The most frequent adverse reactions were grades 1 (mild) or 2 (moderate) in severity (Figs. 3, 4, and Supplementary Table 12). Among participants boosted with NVSI-06–07, 184 (20.47%) reported at least one adverse reaction within 30 days after the boost. And for the groups boosted with a third dose of BBIBP-CorV, the total number of participants reporting any adverse reaction was 177 (19.64%). No statistically significant difference was observed in the occurrence of adverse reactions between these two groups (*P* = 0.6805) (Supplementary Table 12).

The number of individuals reporting any unsolicited adverse event relevant to vaccination was 67 (7.45%) and 66 (7.33%) in heterologous and homologous boosting groups, respectively, within 30 days after booster vaccination (*P* = 0.9285) (Supplementary Table 12). These reported unsolicited adverse reactions were all ranked as grades 1 or 2. No adverse reaction was reported within 30 min. For solicited adverse reactions collected within 7 days after the boost, most of the local and systemic adverse reactions were graded as 1 (mild) or 2 (moderate) in both heterologous and homologous boosting groups, except for grade 3 systemic fever reported by 1 participant (0.11%) in heterologous boosting group and 3 participants (0.33%) in homologous boosting group (Fig. 4 and Supplementary Table 12). The most common injection site adverse reaction within 7 days was pain, reported in 70 (7.79%) subjects in the NVSI-06-07 boosting recipients and 47 (5.22%) in the BBIBP-CorV boosting recipients. Only the pain of grade 1 occurred in NVSI-06-07 booster groups was higher than that in BBIBP-CorV booster groups (*P* = 0.0237), and for all the other local adverse reactions, there was no statistically significant difference between these two boosting schemes (*P* > 0.05) (Fig. 3 and Supplementary Table 12). The most common systemic adverse reactions were headaches, muscle pain (non-inoculation site), fatigue and fever, which were reported in 48 (5.34%), 45 (5.01%), 27 (3.00%) and 18 (2.00%) participants in NVSI-06-07 boosting recipients, and 56 (6.22%), 41 (4.55%), 38 (4.22%) and 21 (2.33%) in the BBIBP-CorV boosting recipients. No statistically significant differences were observed in systemic adverse reactions between the heterologous and homologous boosting groups (*P* > 0.05), except that grade 1 fatigue reported in BBIBP-CorV booster groups was higher than that in NVSI-06-07 booster groups (*P* = 0.0373). (Fig. 4 and Supplementary Table 12).

## Discussion

Findings from this trial showed that both heterologous boost with NVSI-06-07 and homologous boost with BBIBP-CorV were immunogenic in the BBIBP-CorV recipients, but the immunogenicity of heterologous boost was much greater than that of homologous boost. The fold increases in both IgG GMCs and neutralizing antibody GMTs from the corresponding baseline were significantly higher after heterologous boost than those after homologous boost. Especially, for adults primed with BBIBP-CorV over 6 months ago, a 63.85-fold increase in neutralizing antibody GMTs was obtained by heterologous boost, in comparison to 16.78 folds by homologous boost. Compared with the peak value of neutralizing antibody titers primed with two doses of BBIBP-CorV as reported in the previous literature,^23^ neutralizing GMTs boosted by a third dose of BBIBP-CorV were improved by 2.09-3.85 folds on 28 days after the boost, while boosting with NVSI-06-07 induced significant 6.94-13.34-fold increases over the peak value, implying that the neutralizing antibody responses were substantially amplified by heterologous booster vaccination. Among the three tested groups with different prime-boosting intervals, the pre-booster neutralizing antibody level in the ≥6-month group was the lowest, indicating the waning of immunity over time before boosting. However, this group had much higher post-booster neutralizing titers than the other two groups with shorter prime-boosting intervals. The better immune response of a longer prime-boost interval is probably due to additional antibody maturation with increased antibody avidity. Similar observations were also reported in the booster shot of ZF2001, ChAdOx1, and BNT162b2 COVID-19 vaccines.^24–26^ The phenomenon of higher immunogenicity after a wider prime-boost interval has been well recognized in other viral and bacterial vaccines, such as influenza, human papillomavirus, Ebola, DTP (Pertussis, Diphtheria, Tetanus) and Polio vaccines.^27–30^ However, different from the neutralizing antibodies, the RBD-binding IgG level was not increased with longer prime-boost intervals. The results implied that wider dose spacing may contribute to the maturation of neutralizing antibodies, but may have little effects on non-neutralizing antibodies. In addition, our study also showed that the heterologous NVSI-06-07 booter exhibited similar immunogenicity in both older and younger adults. However, due to the number of older participants in the trial was far less than younger participants, this finding should be further validated in the future.

The overall occurrence of adverse reactions was low in both heterologous and homologous boost vaccinations. Most of reported adverse reactions were graded as mild or moderate with the most common symptoms of injection-site pain, headaches, muscle pain (non-inoculation site), fatigues and fever. Reactogenicity of the booster vaccinations was similar to that of the priming vaccinations described in the previously published literatures,^23^ and there was no obvious difference in overall safety between heterologous and homologous boosts.

The heterologous prime-boost combinations among viral vector COVID-19 vaccines, inactivated vaccines and mRNA vaccines have been proved to be able to significantly improve immune responses, and heterologous boost was more immunogenic than homologous boost.^14–16^ All possible prime-boost combinations among Ad26.CoV2.S, mRNA-1273 and BNT162b2 vaccines showed that the neutralizing antibody titer was improved by 4–20 fold after homologous boost and 6 to 73-fold after heterologous boost.^16^ A heterologous booster dose of Convidecia after two doses of CoronaVac elicited a 78.3-fold rise in neutralizing antibody titers, whereas only a 15.2-fold increase was obtained for homologous CoronaVac booster.^15^ The anti-spike IgG antibody concentrations in CoronaVac recipients were improved by 12-fold for homologous boost, and 152, 90 and 77-fold for heterologous BNT162b2, ChAdOx1 and AD26.COV2-S boosts, respectively.^31^ Seven different COVID-19 vaccines (ChAdOx1, BNT162b2, mRNA1273, NVX-CoV2373, Ad26.COV2.S, CVnCoV and VLA2001) as a booster dose following two doses of ChAdOx1 or BNT162b2 induced 1.3–32.3-fold increase in anti-spike IgG levels.^32^ Two small-scale, open-label studies showed that a booster dose of ZF2001 in participants primed with two-dose inactivated vaccines induced 33.9–75.6-fold increases in neutralizing antibody titers.^17–19^ Another two booster vaccination studies illustrated that the pseudo-virus neutralizing antibody titers against wild-type SARS-CoV-2 strain and Omicron variant elicited by ZF2001 booster following two-dose inactivated vaccines were 2.2–3.3-fold and 1.6–2.5-fold higher, respectively, than those induced by a homologous booster of inactivated vaccines.^20,21^ A nationwide cohort study conducted in Sweden showed that the effectiveness against symptomatic COVID-19 infection was 67% and 79% for the heterologous ChAdOx1/BNT162b2 and ChAdOx1/mRNA-1273 prime-boost vaccinations, respectively, which were higher than 50% of the homologous ChAdOx1 vaccination.^14^ The preliminary analysis results of a phase III clinical trial demonstrated that after a heterologous booster vaccination of the recombinant protein subunit vaccine V-01 in inactivated vaccine recipients, the person-year incidence rate of SARS-CoV-2 infections was reduced from 12.80% to 6.73%, and the absolute protective efficacy was 61.35% (https://en.livzon.com.cn/companyfile/1029.html). The results of this trial support the conclusion that the heterologous BBIBP-CorV/NVSI-06-07 prime-boost vaccination scheme serves as another heterologous boosting strategy to better combat SARS-CoV-2. BBIBP-CorV has been approved by many counties for emergency use or conditional marketing, and large-scale populations in the world have completed the primary series of BBIBP-CorV. Considering its high effectiveness and low side-effects, NVSI-06-07 could act as a booster shot to top up immunity against SARS-CoV-2.

Our study showed that heterologous NVSI-06-07 boost not only substantially increased neutralization activity, but also improved the breadth of neutralizing response. Compared to homologous boost with a third dose of BBIBP-CorV, significantly higher neutralizing antibody responses against SARS-CoV-2 VOCs, including Omicron, Alpha, Beta and Delta, were achieved by heterologous boost with NVSI-06-07. The results were consistent with other studies.^17,18^ Especially, owing to high transmissibility and immune-escape capability^33–35^ the Omicron variant has rapidly spread around the world. However, Omicron-specific vaccine is still not available and other strategies are urgently needed to control the pandemic of this variant. Considering that BBIBP-CorV has been applied in large-scale populations and the BBIBP-CorV/NVSI-06-07 prime-booster vaccination can elicit a certain level of neutralizing antibodies against Omicron, this heterologous prime-booster vaccination might serve as a possible strategy combating Omicron.

Many studies have revealed that the levels of neutralizing antibody response were highly correlated with the real-world protection efficacy of the COVID-19 vaccines.^36–41^ According to the previously determined threshold indicative of reduced risks of symptomatic infection,^41^ heterologous prime-boost vaccination of BBIBP-CorV combined with NVSI-06-07 might provide protective effects against SARS-CoV-2 in the real-world.

This study has limitations. First, for the volunteers enrolled in the trial, the number of men was much larger than that of women, and thus the data did not well represent the immune effects on women. Second, the proportion of older individuals aged ≥60 yrs in the participants was small, and the immunogenicity of NVSI-06-07 booster in elderly population should be further assessed in the future. Third, the reference serum used in our live-virus neutralization assays was not the WHO international standard reference material, and the reported results of neutralizing antibody titers were not converted to the international unit. Fourth, data on immune persistence of the booster vaccination is not yet available, and we will report the results once the data have been completed and analyzed. Finally, the cellular immunity was not evaluated in this trial.

In summary, heterologous booster vaccination with NVSI-06-07 in BBIBP-CorV recipients was well tolerated and immunogenic against not only SARS-CoV-2 prototype strain but also the VOCs including Omicron, which supported the approval of emergency use of this heterologous booster strategy.

## Materials and methods

### Trial design and participants

This trial was designed as a phase 2, randomised, double-blinded, controlled trial conducted at a single clinical site in UAE. Eligible participants were healthy adults, aged ≥18 yrs old, who had previously received a full series (two doses) of BBIBP-CorV, a COVID-19 inactivated vaccine. Three groups of participants, receiving their second dose of BBIBP-CorV 1-3 months, 4–6 months or at least 6 months ago, respectively, were enrolled with 600 individuals per group. Female volunteers were not pregnant or breastfeeding, and appropriate contraceptive measures had been taken within 2 weeks before enrollment. Participants needed to understand the trial procedures and were willing to complete the follow-up visits. Participants were screened for health status by inquiry and physical examination, prior to enrollment. Confirmed, suspected or asymptomatic cases of COVID-19 were excluded from the trial. Volunteers who had a history of SARS or MERS infection, or received any COVID-19 vaccine other than the inactivated vaccine BBIBP-CorV were also excluded. Other exclusion criteria include axillary temperature ≥37.3 °C (forehead temperature ≥37.8 °C); a history of severe allergic reactions to previous vaccinations, or allergy to any components of the vaccine; severe respiratory disease, severe liver and kidney diseases; hypertension (systolic blood pressure ≥150 mmHg, diastolic blood pressure ≥90 mmHg), diabetic complications, malignant tumors, various acute diseases or acute attacks of chronic diseases; congenital or acquired immunodeficiency, HIV infection, lymphoma, leukemia or other autoimmune diseases; a history or family history of convulsions, epilepsy, encephalopathy, infectious diseases or mental illness; congenital malformation or developmental disorder, genetic defect, severe malnutrition; a history of coagulation dysfunction (e.g. coagulation factors deficiency and coagulation diseases); asplenia or splenectomy, functional asplenia caused by any situation; under anti-TB (tuberculosis) treatment; receipt of immunoenhancement or inhibitor therapy within 3 months (continuous oral or IV administration for more than 14 days); receipt of other vaccines within 14 days; receipt of blood products within 3 months or other investigational drugs within 6 months; and other situation judged by the investigators that were not suitable for this trial. The detailed inclusion and exclusion criteria can be found on ClinicalTrials.gov (NCT05033847) or in the study protocol (Supplementary Protocol).

The trial protocol was reviewed and approved by Abu Dhabi Health Research and Technology Ethics Committee. The trial was performed in accordance with Good Clinical Practice (GCP), Declaration of Helsinki (with amendments) as well as the local legal and regulatory requirements, and trial safety was overseen by an independent safety monitoring committee. Written informed consent was provided for all participants prior to inclusion into the trial.

### Randomisation and masking

Randomisation was performed using an interactive web response system (IWRS). The randomisation list of participants was generated by the stratified blocked randomization method using SAS software (version 9.4), in which stratification was made according to different time intervals between the second priming dose of BBIBP-CorV and the booster dose, i.e., 1–3 months, 4–6 months and ≥6 months. Within each stratum, participants were randomised using a block randomisation method, with a block size of 10, in a ratio of 1:1 to receive either a heterologous booster dose of NVSI-06-07 or a homologous booster dose of BBIBP-CorV. A vaccine randomisation list with a randomisation block size of 10 was also generated by SAS software. Both the participant and vaccine randomisation lists were inputted into IWRS. At the trial site, according to the randomisation number and the corresponding vaccine number obtained from IWRS, participants were vaccinated accordingly. The trial is double-blind to avoid introducing bias by having randomization and masking process handled by independent personnel from trial operation. Participants, investigators and other staffs remained blinded to individual treatment assignment during the trial.

### Studied vaccines

NVSI-06-07, a recombinant COVID-19 vaccine (CHO cells), encoding a homologous trimeric form of RBD (homo-tri-RBD), was developed by the National Vaccine and Serum Institute (NVSI) and manufactured by Lanzhou Institute of Biological Products Co., Ltd. (LIBP) in accordance with good manufacturing practice (GMP). Homo-tri-RBD was composed of three RBDs from prototype SARS-CoV-2 strain, which were connected end-to-end and co-assembled into a single molecular to possibly mimic the native trimeric arrangements in the natural spike protein.^22^ This vaccine is in the liquid form of 0.5 ml per dose, containing 20 μg antigen and 0.3 mg aluminum hydroxide as adjuvant. The inactivated vaccine BBIBP-CorV, used as a control in this trial, was produced by Beijing Institute of Biological Products Co., Ltd. (BIBP). This vaccine has been approved by WHO for emergency use and applied in large populations. BBIBP-CorV was developed based on the 19nCoV-CDC-Tan-HB02 strain, which was passaged in Vero cells and inactivated by using β-propionolactone.^42^ The vaccine was manufactured in a liquid formulation of 0.5 ml per dose, containing 6.5 U antigen. All vaccines were stored at 2 °C–8 °C prior to use.

### Procedures

After screening, eligible participants received the booster inoculation intramuscularly with NVSI-06-07 or BBIBP-CorV, followed by clinical observation at the study site for no less than 30 min. Within the subsequent 7 days after booster vaccination, local and systemic adverse events (AEs) were self-reported daily by participants using standardized diary cards and verified by investigators. Solicited local AEs included pain, induration, swelling, rash, redness and pruritis. Solicited systemic AEs were fever, diarrhea, constipation, dysphagia, anorexia, vomiting, nausea, muscle pain (systemic), joint pain, headache, cough, breathing trouble, systemic pruritis (no skin damage), abnormal skin mucosa, acute allergic reaction, fatigue and dizziness. Other symptoms were collected as unsolicited AEs. From day 8 to day 30 post-vaccination, unsolicited AEs were recorded by participants in contact cards. Assessments were performed by study investigators to confirm subject safety. Serious adverse events (SAEs) and adverse events of special interest (AESIs) were monitored up to 6 months after vaccination. Safety oversight for specific vaccination pause rules and for advancement was done by an independent safety monitoring committee. The grade of AEs was assessed according to the relevant guidance of China National Medical Products Administration (NMPA). The causal relationship between adverse events and vaccination was determined by the investigators.

Blood samples were collected from the participants before booster vaccination, and on days 14 and 28 after the boost. The immunogenicity was assessed by RBD-specific binding antibody responses (IgG) and neutralizing antibody activities against live SARS-CoV-2 virus. The corresponding seroconversion rates, defined as ≥4-fold rise in IgG concentrations or neutralizing titers were determined based on the detected pre-booster and post-booster IgG or neutralizing antibody levels. In order to evaluate cross-neutralizing activities, besides prototype SARS-CoV-2 live virus, several VOCs, including Omicron, Alpha, Beta and Delta strains, were also tested in the neutralization assay for a subset of serum samples.

### Laboratory tests

IgG level specific to prototype RBD was measured using a magnetic chemiluminescence enzyme immunoassay kit purchased from Bioscience (Chongqing) Biotechnology Co. (approved by the China National Medical Products Administration; approval numbers 20203400183). Serum samples were heat-inactivated at 56 °C for 30 min, and then diluted to ensure the concentrations to be within the calibration range of the kit. The IgG concentration detections were performed on an automated chemiluminescence detector (Axceed 260) according to the manufacturer’s detailed instructions. The reference calibrator used in the kit has been calibrated using the WHO International Standard for anti-SARS-CoV-2 immunoglobulin (NIBSC code: 20/136).

Neutralizing antibody titer was detected using live-virus neutralization assay as described in our previous studies.^22^ Briefly, heat-inactivated human serum samples were diluted by a two-fold dilution series starting from an initial factor of 1:4 (in detection of neutralizing antibodies against SARS-CoV-2 prototype strain) or 1:10 (in detection of neutralizing antibodies against VOCs). Serum dilutions were then mixed with the same volume of 100 50% tissue culture infectious dose (TCID_50_) of SARS-CoV-2 live virus per well. After incubated at 37 °C for 2 h, Vero cells with a density of 1.5–2 × 10^5^ cells per mL were added into the well and subsequently incubated in a 5% CO_2_ incubator at 37 °C for 3–5 days. Both positive and negative reference serum controls were included in each assay. Neutralizing antibody titer was reported as the reciprocal of the highest serum dilution that protected 50% of cells from virus infection. The titer of the measurement below the lower limit of detection was assigned a value of half the detection limit. All the live-virus neutralization assays were carried out in the Biosafety Level 3 laboratory of National Institute for Viral Disease Control and Prevention, Chinese Center for Disease Control and Prevention (China CDC), Beijing, China. The live viruses of SARS-CoV-2 prototype (QD-01), Alpha (BJ-210122-14), Beta (GD84), Delta (GD96) and Omicron (NPRC2.192100003) strains were tested in the assays.

### Outcomes

The primary outcome was the comparative assessment of immunogenicity between heterologous and homologous booster vaccinations on 14 and 18 days after the boost. The secondary outcomes were safety profile within 30 min, and 7 and 30 days of booster vaccination. The exploratory outcome was the immunity against Omicron and other VOCs. Safety was assessed by the occurrence of all SAEs and AESIs, and the occurrence of the solicited or unsolicited adverse reactions within 30 days after vaccination. The occurrence and severity of adverse reactions were compared between heterologous NVSI-06-07 booster groups and the homologous BBIBP-CorV booster groups. The immunogenicity was evaluated by geometric mean concentrations (GMCs) of RBD-binding antibody IgG and geometric mean titers (GMTs) of live-virus neutralizing antibodies, as well as the corresponding seroconversion rate, on 14 and 28 days after booster vaccination. The comparisons of the immunogenicity between the heterologous and homologous booster groups were also carried out. The immunity against Omicron and other VOCs evaluated was determined using live-virus neutralizing antibody GMTs.

### Statistical analysis

Assuming that a 4-fold rise in neutralizing antibody titers for both heterogeneous and homologous booster groups reached at 85% and the non-inferiority threshold was set to -10%, the sample size was determined to be 208 using Miettinen & Nurminen method to achieve 80% power at one-sided significance level of 2.5%.^43^ Assuming that the neutralizing antibody GMTs between heterologous and homologous boosting groups are comparable, with the standard deviation (SD) of GMT after log10 transformation to be 0.7, and the non-inferiority threshold was set to 2/3, 250 participants per group was needed to achieve 80% power at one-sided significance level of 2.5%. Considering the above estimations and 15%~20% drop-out rate, 600 participants were enrolled into each of the three boosting groups (1–3 months, 4–6 months and ≥6 months). Half participants of each group were assigned to heterologous booster and the other half were assigned to homologous booster. Thus, a total of 1800 individuals (900 in heterologous groups and 900 in homogeneous groups) participated the trial.

For statistical analysis, the full analysis set (FAS), safety set (SS), per-protocol set 1 (PPS1) and PPS2 were defined. FAS included all participants who were randomly assigned to treatment and received the booster dose of vaccination. SS contained all participants who received the booster dose of vaccination. PPS1 and PPS2 included all participants who received the booster dose of vaccination and completed the follow-up visit on day 14 and 28 post-vaccination, respectively. Baseline characteristics were evaluated on FAS. Continuous variables were analyzed using Student’s t-test and categorical variables were analyzed with Chi-square test. Safety analysis was performed on SS, and immunogenicity analysis was carried out on PPS. RBD-specific IgG levels and the live-virus neutralizing antibody activities were presented by GMCs and GMTs, respectively. Additionally, based on pre-booster and post-booster values, 4-fold increase in antibody concentration or titer were calculated, with 95% confidence intervals (CIs) of seroconversion rate calculated using Clopper–Pearson method.^44^ Cochran–Mantel–Haenszel (CMH) method considering stratification factors was used to compare the proportion differences between heterologous and homologous booster groups.^45^ RBD-specific IgG concentrations and neutralizing antibody titers between the heterologous and homologous booster groups were compared after logarithmic conversion. For safety analysis, the number and proportion of participants reporting at least one adverse reaction post-vaccination were analyzed and differences between groups were compared using Fisher’s exact test (SAS Institute Inc. SAS/STAT^®^ User’s Guide). All statistical analyses were carried out using SAS software (version 9.4). All statistical tests were two-sided, and the statistical significance level was *P* < 0.05.

## Supplementary information

Supplementary Tables S1-S12

Supplementary Protocol
