# Supplementary material for: Immunogenicity and safety of NVSI-06-07 as a heterologous booster after priming with BBIBP-CorV: a phase 2 trial
Source: Signal Transduct Target Ther. 2022 Jun 6;7:172. doi: 10.1038/s41392-022-00984-2 (PMC9167817; doi:10.1038/s41392-022-00984-2)
Supplement: Supplementary file 1 — Supplementary Tables S1-S12 [file 41392_2022_984_MOESM1_ESM.docx]

Supplementary Materials for

Immunogenicity and safety of NVSI-06-07 as a heterologous booster after priming with BBIBP-CorV: a phase 2 trial

Nawal Al Kaabi, Yun Kai Yang, Jing Zhang, Ke Xu, Yu Liang, Yun Kang, Ji Guo Su, Tian Yang, Salah Hussein, Mohamed Saif ElDein, Shuai Shao, Sen Sen Yang, Wenwen Lei, Xue Jun Gao, Zhiwei Jiang, Hui Wang, Meng Li, Hanadi Mekki Mekki, Walid Zaher, Sally Mahmoud, Xue Zhang, Chang Qu, Dan Ying Liu, Jing Zhang, Mengjie Yang, Islam Eltantawy, Peng Xiao, Zhao Nian Wang, Jin Liang Yin, Xiao Yan Mao, Jin Zhang, Ning Liu, Fu Jie Shen, Liang Qu, Yun Tao Zhang, Xiao Ming Yang, Guizhen Wu, Qi Ming Li

Correspondence to: Qi Ming Li ([liqiming189@163.com](mailto:liqiming189@163.com)) or Guizhen Wu ([wugz@ivdc.chinacdc.cn](mailto:wugz@ivdc.chinacdc.cn)) or Xiao Ming Yang ([yangxiaoming@sinopharm.com](mailto:yangxiaoming@sinopharm.com)) or Yun Tao Zhang ([zhangyuntao@sinopharm.com](mailto:zhangyuntao@sinopharm.com))

**This PDF file includes:**

Tables S1 to S12.

**Table S1:** Baseline characteristic for the nationality of the participants (FAS)

|  | 1-3 months | | |  | 4-6 months | | |  | ≥6 months | | |
| --- | --- | --- | --- | --- | --- | --- | --- | --- | --- | --- | --- |
|  | NVSI-06-07 (N=301) | BBIBP-CorV (N=299) | P value |  | NVSI-06-07 (N=300) | BBIBP-CorV (N=300) | P value |  | NVSI-06-07 (N=298) | BBIBP-CorV (N=302) | P value |
| Countries, n(%) |  |  | 0.3109 |  |  |  | 0.2941 |  |  |  | 0.7785 |
| Afghanistan | 9 (2.99) | 15 (5.02) |  |  | 2 (0.67) | 1 (0.33) |  |  | 0 (0.00) | 1 (0.33) |  |
| Bangladesh | 96 (31.89) | 76 (25.42) |  |  | 53 (17.67) | 49 (16.33) |  |  | 15 (5.03) | 15 (4.97) |  |
| Cameroon | 1 (0.33) | 2 (0.67) |  |  | 0 (0.00) | 0 (0.00) |  |  | 1 (0.34) | 1 (0.33) |  |
| Canada | 0 (0.00) | 1 (0.33) |  |  | 0 (0.00) | 0 (0.00) |  |  | 0 (0.00) | 0 (0.00) |  |
| Chad | 0 (0.00) | 0 (0.00) |  |  | 0 (0.00) | 0 (0.00) |  |  | 1 (0.34) | 0 (0.00) |  |
| Comorin | 1 (0.33) | 0 (0.00) |  |  | 0 (0.00) | 0 (0.00) |  |  | 0 (0.00) | 0 (0.00) |  |
| Egypt | 5 (1.66) | 8 (2.68) |  |  | 4 (1.33) | 8 (2.67) |  |  | 7 (2.35) | 13 (4.30) |  |
| United Arab Emirates | 0 (0.00) | 0 (0.00) |  |  | 2 (0.67) | 0 (0.00) |  |  | 15 (5.03) | 18 (5.96) |  |
| Ethiopia | 0 (0.00) | 0 (0.00) |  |  | 0 (0.00) | 0 (0.00) |  |  | 1 (0.34) | 0 (0.00) |  |
| Falkland Islands | 0 (0.00) | 0 (0.00) |  |  | 1 (0.33) | 0 (0.00) |  |  | 0 (0.00) | 0 (0.00) |  |
| Philippines | 30 (9.97) | 36 (12.04) |  |  | 12 (4.00) | 22 (7.33) |  |  | 26 (8.72) | 28 (9.27) |  |
| Ghana | 0 (0.00) | 0 (0.00) |  |  | 0 (0.00) | 1 (0.33) |  |  | 6 (2.01) | 5 (1.66) |  |
| India | 44 (14.62) | 46 (15.38) |  |  | 91 (30.33) | 84 (28.00) |  |  | 52 (17.45) | 47 (15.56) |  |
| Indonesia | 0 (0.00) | 0 (0.00) |  |  | 1 (0.33) | 0 (0.00) |  |  | 0 (0.00) | 0 (0.00) |  |
| Iran | 0 (0.00) | 1 (0.33) |  |  | 0 (0.00) | 0 (0.00) |  |  | 0 (0.00) | 0 (0.00) |  |
| Iraq | 1 (0.33) | 1 (0.33) |  |  | 0 (0.00) | 0 (0.00) |  |  | 0 (0.00) | 0 (0.00) |  |
| Jordan | 5 (1.66) | 11 (3.68) |  |  | 0 (0.00) | 0 (0.00) |  |  | 1 (0.34) | 0 (0.00) |  |
| Korea | 0 (0.00) | 0 (0.00) |  |  | 0 (0.00) | 0 (0.00) |  |  | 0 (0.00) | 0 (0.00) |  |
| Lebanon | 0 (0.00) | 0 (0.00) |  |  | 0 (0.00) | 0 (0.00) |  |  | 1 (0.34) | 0 (0.00) |  |
| Mauritania | 0 (0.00) | 0 (0.00) |  |  | 0 (0.00) | 0 (0.00) |  |  | 0 (0.00) | 2 (0.66) |  |
| Morocco | 5 (1.66) | 1 (0.33) |  |  | 4 (1.33) | 2 (0.67) |  |  | 8 (2.68) | 5 (1.66) |  |
| Nepal | 10 (3.32) | 16 (5.35) |  |  | 12 (4.00) | 21 (7.00) |  |  | 35 (11.74) | 36 (11.92) |  |
| Nigeria | 1 (0.33) | 0 (0.00) |  |  | 0 (0.00) | 1 (0.33) |  |  | 0 (0.00) | 0 (0.00) |  |
| Oman | 0 (0.00) | 3 (1.00) |  |  | 0 (0.00) | 0 (0.00) |  |  | 0 (0.00) | 0 (0.00) |  |
| Pakistan | 80 (26.58) | 66 (22.07) |  |  | 115 (38.33) | 105 (35.00) |  |  | 106 (35.57) | 104 (34.44) |  |
| Palestine | 4 (1.33) | 6 (2.01) |  |  | 0 (0.00) | 2 (0.67) |  |  | 0 (0.00) | 0 (0.00) |  |
| Sierra Leone | 0 (0.00) | 0 (0.00) |  |  | 0 (0.00) | 0 (0.00) |  |  | 1 (0.34) | 1 (0.33) |  |
| Sri Lanka | 0 (0.00) | 1 (0.33) |  |  | 1 (0.33) | 0 (0.00) |  |  | 0 (0.00) | 2 (0.66) |  |
| Sudan | 2 (0.66) | 3 (1.00) |  |  | 0 (0.00) | 1 (0.33) |  |  | 9 (3.02) | 8 (2.65) |  |
| Syria | 1 (0.33) | 0 (0.00) |  |  | 0 (0.00) | 0 (0.00) |  |  | 2 (0.67) | 0 (0.00) |  |
| Tanzania | 1 (0.33) | 0 (0.00) |  |  | 0 (0.00) | 0 (0.00) |  |  | 0 (0.00) | 0 (0.00) |  |
| Tunisia | 0 (0.00) | 0 (0.00) |  |  | 0 (0.00) | 0 (0.00) |  |  | 0 (0.00) | 2 (0.66) |  |
| Uganda | 5 (1.66) | 4 (1.34) |  |  | 2 (0.67) | 2 (0.67) |  |  | 8 (2.68) | 11 (3.64) |  |
| Yemen | 0 (0.00) | 0 (0.00) |  |  | 0 (0.00) | 1 (0.33) |  |  | 3 (1.01) | 3 (0.99) |  |
| Germany | 0 (0.00) | 1 (0.33) |  |  | 0 (0.00) | 0 (0.00) |  |  | 0 (0.00) | 0 (0.00) |  |
| Iberia | 0 (0.00) | 1 (0.33) |  |  | 0 (0.00) | 0 (0.00) |  |  | 0 (0.00) | 0 (0.00) |  |
| Other | 0 (0.00) | 0 (0.00) |  |  | 0 (0.00) | 0 (0.00) |  |  | 0 (0.00) | 0 (0.00) |  |

**Table S2:** The difference in the increase of neutralizing antibody titers between three groups (1-3 months, 4-6 months, and ≥6 months) compared by using covariance analysis models^a^

|  | 14 days |  | 28 days |
| --- | --- | --- | --- |
|  | ≥6 months |  | ≥6 months |
| NVSI-06-07 |  |  |  |
| Scheffe |  |  |  |
| 1-3 months | <0.0001 |  | <0.0001 |
| 4-6 months | 0.0035 |  | <0.0001 |
| Sidak |  |  |  |
| 1-3 months | <0.0001 |  | <0.0001 |
| 4-6 months | 0.0023 |  | <0.0001 |
| Bonferroni |  |  |  |
| 1-3 months | <0.0001 |  | <0.0001 |
| 4-6 months | 0.0023 |  | <0.0001 |
| BBIBP-CorV |  |  |  |
| Scheffe |  |  |  |
| 1-3 months | <0.0001 |  | <0.0001 |
| 4-6 months | <0.0001 |  | <0.0001 |
| Sidak |  |  |  |
| 1-3 months | <0.0001 |  | <0.0001 |
| 4-6 months | <0.0001 |  | <0.0001 |
| Bonferroni |  |  |  |
| 1-3 months | <0.0001 |  | <0.0001 |
| 4-6 months | <0.0001 |  | <0.0001 |

^a^The log-transformed antibody titers after boost were used as the response variables, and the log-transformed values of the antibody titers before booster immunization were used as covariates. The prime-boost time interval, i.e., 1-3 months, 4-6 months and ≥6 months, was used as a fixed effect, and post hoc analysis was performed using Scheffe, Sidak and Bonferroni methods

**Table S3:** RBD-specific IgG Response Results for different age subgroups (14 days after boosting) (PPS1)

|  | <45 years old | | |  | >=45 years old | | |
| --- | --- | --- | --- | --- | --- | --- | --- |
|  | NVSI-06-07 | BBIBP-CorV | P value |  | NVSI-06-07 | BBIBP-CorV | P value |
| 1-3 months |  |  |  |  |  |  |  |
| N(missing) | 243(0) | 243(0) |  |  | 24(0) | 27(0) |  |
| Pre-booster antibody GMC^a^ (95%CI) | 109.10(92.40-128.82) | 104.77(90.20-121.68) | 0.7213 |  | 121.26(57.25-256.83) | 163.04(82.21-323.33) | 0.5499 |
| Post-booster antibody GMC (95%CI) | 4978.47(4282.81-5787.13) | 289.78(263.51-263.51) |  |  | 3171.80(1474.51-6822.80) | 433.90(304.70-617.88) |  |
| Post-booster antibody GMC adjusted between NVSI-06-07 and BBIBP-CorV groups (95%CI) | 4951.16(4399.46-5572.04) | 291.37(258.91-327.91) |  |  | 3452.27(2246.04-5306.28) | 402.42(268.35-603.46) |  |
| Ratio of adjusted GMC between NVSI-06-07 and BBIBP-CorV groups (95% CI)^b^ | 16.99(14.38-20.08) |  | <0.0001^c^ |  | 8.58(4.75-15.50) |  | <0.0001^c^ |
| Post-booster antibody GMC adjusted between different age subgroups (95%CI) | 4996.50(4322.05-5776.21) |  |  |  | 3057.80(1927.43-4851.09) |  |  |
| Ratio of adjusted GMC between two age subgroups (95%CI)^d^ | 1.63(1.01-2.65) |  |  |  | P=0.0467 |  |  |
| Rate of seroconversion^e^, n (%) | 227(93.42) | 57(23.46) | <0.0001 |  | 22(91.67) | 7(25.93) | <0.0001 |
| 95%CI (%) | 89.53-96.19 | 18.28-29.30 |  |  | 73.00-98.97 | 11.11-46.28 |  |
| Rate difference between NVSI-06-07 and BBIBP-CorV groups (%, 95%CI)^f^ | 69.96(63.79-76.13) |  |  |  | 65.74(45.85-85.63) |  |  |
| Post-booster antibody GMC fold rise (95%CI) | 45.63(38.08-54.68) | 2.77(2.39-3.21) | <0.0001 |  | 26.16(14.96-45.73) | 2.66(1.59-4.45) | <0.0001 |
| 4-6 months |  |  |  |  |  |  |  |
| N(missing) | 254(0) | 254(0) |  |  | 25(0) | 36(0) |  |
| Pre-booster antibody GMC^a^ (95%CI) | 141.96(117.71-171.19) | 174.73(143.82-212.29) | 0.1306 |  | 308.10(181.34-523.48) | 145.98(84.39-252.50) | 0.0593 |
| Post-booster antibody GMC (95%CI) | 6804.48(5956.61-7773.03) | 444.29(393.95-501.05) |  |  | 6738.21(4195.59-10821.70) | 483.06(355.35-656.67) |  |
| Post-booster antibody GMC adjusted between NVSI-06-07 and BBIBP-CorV groups (95%CI) | 6990.94(6221.28-7855.83) | 432.44(384.83-485.93) |  |  | 6085.33(4115.03-8999.00) | 518.49(374.90 -717.06) |  |
| Ratio of adjusted GMC between NVSI-06-07 and BBIBP-CorV groups (95% CI)^b^ | 16.17(13.71-19.07) |  | <0.0001^c^ |  | 11.74(7.01-19.65) |  | <0.0001^c^ |
| Post-booster antibody GMC adjusted between different age subgroups (95%CI) | 5998.19(3940.36-9130.73) |  |  |  | 6882.84(6039.85-7843.48) |  |  |
| Ratio of adjusted GMC between two age subgroups (95%CI)^d^ | 0.87 (0.56-1.35) |  |  |  | P=0.5395 |  |  |
| Rate of seroconversion^e^, n (%) | 229(90.16) | 65(25.59) | <0.0001 |  | 23(92.00) | 8(22.22) | <0.0001 |
| 95%CI (%) | 85.81-93.53 | 20.34-31.42 |  |  | 73.97-99.02 | 10.12-39.15 |  |
| Rate difference between NVSI-06-07 and BBIBP-CorV groups (%, 95%CI)^f^ | 64.57(58.07-71.06) |  |  |  | 69.78(52.53-87.03) |  |  |
| Post-booster antibody GMC fold rise (95%CI) | 47.93(39.11-58.75) | 2.54(2.17-2.99) | <0.0001 |  | 21.87(11.74-40.75) | 3.31(2.01-5.46) | <0.0001 |
| ≥6 months |  |  |  |  |  |  |  |
| N(missing) | 238(0) | 237(0) |  |  | 43(0) | 48(0) |  |
| Pre-booster antibody GMC^a^ (95%CI) | 106.20(81.36-138.61) | 127.86(98.10-166.64) | 0.3308 |  | 98.23(50.17-192.34) | 117.16(57.94-236.87) | 0.7178 |
| Post-booster antibody GMC (95%CI) | 5891.81(5075.26-6839.73) | 565.69(493.26-648.75) |  |  | 6928.42(4455.30-10774.4) | 752.38(492.58-1149.21) |  |
| Post-booster antibody GMC adjusted between NVSI-06-07 and BBIBP-CorV groups (95%CI) | 5987.31(5229.41-6855.06) | 556.63(486.03 -637.49) |  |  | 7110.93(4791.84-10552.39) | 735.06(505.91 -1067.98) |  |
| Ratio of adjusted GMC between NVSI-06-07 and BBIBP-CorV groups (95% CI)^b^ | 10.76(8.88-13.03) |  | <0.0001^c^ |  | 9.67(5.62-16.66) |  | <0.0001^c^ |
| Post-booster antibody GMC adjusted between different age subgroups (95%CI) | 5881.80(5061.75-6834.71) |  |  |  | 6993.91(4912.43-9957.37) |  |  |
| Ratio of adjusted GMC between two age subgroups (95%CI)^d^ | 0.84(0.57-1.23) |  |  |  | P=0.3753 |  |  |
| Rate of seroconversion^e^, n (%) | 202(84.87) | 83(35.02) | <0.0001 |  | 39(90.70) | 20(41.67) | <0.0001 |
| 95%CI (%) | 79.68-89.18 | 28.96-41.46 |  |  | 77.86-97.41 | 27.61-56.79 |  |
| Rate difference between NVSI-06-07 and BBIBP-CorV groups (%, 95%CI)^f^ | 49.85(42.26-57.44) |  |  |  | 49.03(32.60-65.46) |  |  |
| Post-booster antibody GMC fold rise (95%CI) | 55.48(41.96-73.36) | 4.42(3.50-5.59) | <0.0001 |  | 70.53(38.98-127.62) | 6.42(3.33-12.38) | <0.0001 |

^a^GMC represents geometric mean concentration, and the unit of GMC is BAU/ml

^b^The ratio of adjusted GMC between two groups was calculated by “NVSI-06-07/ BBIBP-CorV”

^c^Covariance analysis with least square method was used to calculate the adjusted GMC and P value

^d^The ratio of adjusted GMC between two age subgroups was calculated by “<45 years old / ≥45 years old”

^e^Seroconversion was defined as more than 4-fold rise form baseline in IgG concentration

^f^Rate difference=(NVSI-06-07)-(BBIBP-CorV). Rate difference and 95%CI were estimated by CMH method considering stratification factors

**Table S4:** RBD-specific IgG Response Results for different age subgroups (14 days after boosting) (PPS1) ( >=45 years old)

|  | <60 years old | | |  | >=60 years old | | |
| --- | --- | --- | --- | --- | --- | --- | --- |
|  | NVSI-06-07 | BBIBP-CorV | P value |  | NVSI-06-07 | BBIBP-CorV | P value |
| 1-3 months |  |  |  |  |  |  |  |
| N(missing) | 19(0) | 20(0) |  |  | 5(0) | 7(0) |  |
| Pre-booster antibody GMC^a^ (95%CI) | 100.20(46.05-218.00) | 167.73(73.80-381.25) | 0.3466 |  | 250.36(13.17-4761.05) | 150.35(28.57-791.26) | 0.6793 |
| Post-booster antibody GMC (95%CI) | 2872.20(1175.71-7016.67) | 440.35(287.82-673.71) |  |  | 4624.34(494.54-43241.7) | 415.98(177.93-972.50) |  |
| Post-booster antibody GMC adjusted between NVSI-06-07 and BBIBP-CorV groups (95%CI) | 3301.39(1920.01-5676.62) | 385.78(227.50-654.18) |  |  | 3895.96(1956.24-7759.05) | 470.16(262.88-840.87) |  |
| Ratio of adjusted GMC between NVSI-06-07 and BBIBP-CorV groups (95% CI)^b^ | 8.56(4.00-18.32) |  | <0.0001^c^ |  | 8.29(3.35-20.49) |  | 0.0005^c^ |
| Post-booster antibody GMC adjusted between different age subgroups (95%CI) | 3316.90(1781.19-6176.69) |  |  |  | 2675.99(783.22-9142.93) |  |  |
| Ratio of adjusted GMC between two age subgroups (95%CI)^d^ | 1.24(0.31-4.96) |  |  |  | P=0.7508 |  |  |
| Rate of seroconversion^e^, n (%) | 18(94.74) | 4(20.00) | <0.0001 |  | 4(80.00) | 3(42.86) | 0.2929 |
| 95%CI (%) | 73.97-99.87 | 5.73-43.66 |  |  | 28.36-99.49 | 9.90-81.59 |  |
| Rate difference between NVSI-06-07 and BBIBP-CorV groups (%, 95%CI)^f^ | 74.74(54.53-94.94) |  |  |  | 3714(-13.58-87.87) |  |  |
| Post-booster antibody GMC fold rise (95%CI) | 28.66(14.84-55.37) | 2.63(1.35-5.10) | <0.0001 |  | 18.47(4.09-83.43) | 2.77(1.12-6.84) | 0.0132 |
| 4-6 months |  |  |  |  |  |  |  |
| N(missing) | 24(0) | 33(0) |  |  | 1(0) | 3(0) |  |
| Pre-booster antibody GMC^a^ (95%CI) | 301.49 (173.61-523.57) | 158.62 (90.51-277.98) | 0.1099 |  | 518.50 | 58.53(0.30-11239.7) | -^g^ |
| Post-booster antibody GMC (95%CI) | 6916.73 (4229.55-11311.1) | 473.99(345.67-649.94) |  |  | 3597.50 | 595.04(24.32-14557.7) |  |
| Post-booster antibody GMC adjusted between NVSI-06-07 and BBIBP-CorV groups (95%CI) | 6278.33 (4237.72-9301.56) | 508.58 (364.18-710.23) |  |  | 3445.12 | 603.69 |  |
| Ratio of adjusted GMC between NVSI-06-07 and BBIBP-CorV groups (95% CI)^b^ | 12.34 (7.33-20.80) |  | <0.0001^c^ |  | 5.71 |  | -^g^ |
| Post-booster antibody GMC adjusted between different age subgroups (95%CI) | 6949.88 (4263.78-11328.15) |  |  |  | 3207.49 (290.58-35405.29) |  |  |
| Ratio of adjusted GMC between two age subgroups (95%CI)^d^ | 2.17(0.19-25.14) |  |  |  | P=0.5198 |  |  |
| Rate of seroconversion^e^, n (%) | 22(91.67) | 7(21.21) | <0.0001 |  | 1(100.00) | 1(33.33) | -^g^ |
| 95%CI (%) | 73.00-98.97 | 8.98-38.91 |  |  | 2.50-100.00 | 0.84-90.57 |  |
| Rate difference between NVSI-06-07 and BBIBP-CorV groups (%, 95%CI)^f^ | 70.45(52.66-88.25) |  |  |  | 66.67(13.32-100.00) |  |  |
| Post-booster antibody GMC fold rise (95%CI) | 22.94(12.07-43.60) | 2.99(1.83-4.87) | <0.0001 |  | 6.94 | 10.17 (0.02-4241.05) | -^g^ |
| ≥6 months |  |  |  |  |  |  |  |
| N(missing) | 39(0) | 43(0) |  |  | 4(0) | 5(0) |  |
| Pre-booster antibody GMC^a^ (95%CI) | 106.56(52.38-216.80) | 149.08 (71.53-310.69) | 0.5102 |  | 44.41(1.26-1561.49) | 14.75(1.54-140.76) | 0.4400 |
| Post-booster antibody GMC (95%CI) | 7092.87(4505.24-11166.7) | 783.76 (490.79-1251.63) |  |  | 5511.93(236.92-128236.00) | 529.43(212.42-1319.52) |  |
| Post-booster antibody GMC adjusted between NVSI-06-07 and BBIBP-CorV groups (95%CI) | 7451.81(4883.28-11371.35) | 749.44(501.14 -1120.78) |  |  | 4410.91(860.73-22604.38) | 632.74 (147.81-2708.68) |  |
| Ratio of adjusted GMC between NVSI-06-07 and BBIBP-CorV groups (95% CI)^b^ | 9.94(5.54-17.84) |  | <0.0001^c^ |  | 6.97(0.74-65.23) |  | 0.0778^c^ |
| Post-booster antibody GMC adjusted between different age subgroups (95%CI) | 6906.50(4569.12-10439.60) |  |  |  | 7145.82(1952.54-26152.00) |  |  |
| Ratio of adjusted GMC between two age subgroups (95%CI)^d^ | 0.97(0.25-3.78) |  |  |  | P=0.9600 |  |  |
| Rate of seroconversion^e^, n (%) | 35(89.74) | 16(37.21) | <0.0001 |  | 4(100.00) | 4(80.00) | 1.0000 |
| 95%CI (%) | 75.78-97.13 | 22.98-53.27 |  |  | 39.76-100.00 | 28.36-99.49 |  |
| Rate difference between NVSI-06-07 and BBIBP-CorV groups (%, 95%CI)^f^ | 52.53(35.23-69.84) |  |  |  | 20.00(-15.06-55.06) |  |  |
| Post-booster antibody GMC fold rise (95%CI) | 66.56(34.64-127.91) | 5.26(2.67-10.33) | <0.0001 |  | 124.10(81.25-189.576) | 35.90(2.04-633.28) | 0.2979 |

^a^GMC represents geometric mean concentration, and the unit of GMC is BAU/ml

^b^The ratio of adjusted GMC between two groups was calculated by “NVSI-06-07/ BBIBP-CorV”

^c^Covariance analysis with least square method was used to calculate the adjusted GMC and P value

^d^The ratio of adjusted GMC between two age subgroups was calculated by “<45 years old / ≥45 years old”

^e^Seroconversion was defined as more than 4-fold rise form baseline in IgG concentration

^f^Rate difference=(NVSI-06-07)-(BBIBP-CorV). Rate difference and 95%CI were estimated by CMH method considering stratification factors

^g^Due to only one participant in the subgroup, P value was not calculated

**Table S5:** RBD-specific IgG Response Results for different age subgroups (28days after boosting) (PPS2)

|  | <45 years old | | |  | >=45 years old | | |
| --- | --- | --- | --- | --- | --- | --- | --- |
|  | NVSI-06-07 | BBIBP-CorV | P value |  | NVSI-06-07 | BBIBP-CorV | P value |
| 1-3 months |  |  |  |  |  |  |  |
| N(missing) | 231(0) | 222(0) |  |  | 24(0) | 25(0) |  |
| Pre-booster antibody GMC^a^ (95%CI) | 105.06(88.60-124.58) | 101.36(86.64-118.58) | 0.7609 |  | 123.04(58.36-259.42) | 162.21(80.80-325.64) | 0.5782 |
| Post-booster antibody GMC (95%CI) | 3391.21(2948.89-3899.89) | 245.56(223.07-270.32) |  |  | 2574.24(1288.75-5141.98) | 353.45(241.62-517.05) |  |
| Post-booster antibody GMC adjusted between NVSI-06-07 and BBIBP-CorV groups (95%CI) | 3372.48(3027.43-3756.84) | 246.98(221.24-275.73) |  |  | 2787.37(1922.08-4042.19) | 327.47(227.52-471.32) |  |
| Ratio of adjusted GMC between NVSI-06-07 and BBIBP-CorV groups (95% CI)^b^ | 13.65(11.70-15.93) |  | <0.0001^c^ |  | 8.51(5.05-14.33) |  | <0.0001^c^ |
| Post-booster antibody GMC adjusted between different age subgroups (95%CI) | 3412.14(2999.92-3881.00) |  |  |  | 2426.25(1626.96-3618.22) |  |  |
| Ratio of adjusted GMC between two age subgroups (95%CI)^d^ | 1.41(0.92-2.14) |  |  |  | P=0.1110 |  |  |
| Rate of seroconversion^e^, n (%) | 215(93.07) | 49(22.07) | <0.0001 |  | 22(91.67) | 5(20.00) | <0.0001 |
| 95%CI (%) | 89.00-95.99 | 16.80-28.11 |  |  | 73.00-98.97 | 6.83-40.70 |  |
| Rate difference between NVSI-06-07 and BBIBP-CorV groups (%, 95%CI)^f^ | 71.00(64.64-77.36) |  |  |  | 71.67(52.48-90.85) |  |  |
| Post-booster antibody GMC fold rise (95%CI) | 32.28(27.34-38.10) | 2.42(2.09-2.80) | <0.0001 |  | 20.92(12.59-34.76) | 2.18(1.36-3.48) | <0.0001 |
| 4-6 months |  |  |  |  |  |  |  |
| N(missing) | 225(0) | 218(0) |  |  | 24(0) | 32(0) |  |
| Pre-booster antibody GMC^a^ (95%CI) | 141.84(116.16-173.20) | 197.27(159.65-243.75) | 0.0259 |  | 267.20(141.27-505.38) | 121.90(71.10-208.97) | 0.0581 |
| Post-booster antibody GMC (95%CI) | 4985.59(4404.68-5643.13) | 365.02(321.60-414.30) |  |  | 4397.73(2591.61-7462.53) | 364.09(260.00-509.86) |  |
| Post-booster antibody GMC adjusted between NVSI-06-07 and BBIBP-CorV groups (95%CI) | 5233.47(4695.78-5832.72) | 347.19(310.98-387.62) |  |  | 3955.80(2572.35-6083.28) | 394.19(272.03-571.21) |  |
| Ratio of adjusted GMC between NVSI-06-07 and BBIBP-CorV groups (95% CI)^b^ | 15.07(12.91-17.60) |  | <0.0001^c^ |  | 10.04(5.63-17.88) |  | <0.0001^c^ |
| Post-booster antibody GMC adjusted between different age subgroups (95%CI) | 3927.93(2694.98 -5724.97) |  |  |  | 5046.04(4465.21-5702.42) |  |  |
| Ratio of adjusted GMC between two age subgroups (95%CI)^d^ | 0.78 (0.52-1.16) |  |  |  | 0.2147 |  |  |
| Rate of seroconversion^e^, n (%) | 202(89.78) | 38(17.43) | <0.0001 |  | 21(87.50) | 6(18.75) | <0.0001 |
| 95%CI (%) | 85.06-93.41 | 12.64-23.13 |  |  | 67.64-97.34 | 7.21-36.44 |  |
| Rate difference between NVSI-06-07 and BBIBP-CorV groups (%, 95%CI)^f^ | 72.35(65.94-78.75) |  |  |  | 68.75(49.83-87.67) |  |  |
| Post-booster antibody GMC fold rise (95%CI) | 35.15(28.73-43.00) | 1.85(1.58-2.16) | <0.0001 |  | 16.46(8.69-31.18) | 2.99(1.71-5.23) | 0.0001 |
| ≥6 months |  |  |  |  |  |  |  |
| N(missing) | 203(0) | 205(0) |  |  | 38(0) | 46(0) |  |
| Pre-booster antibody GMC^a^ (95%CI) | 112.42(84.69-149.25) | 140.00(106.55-183.95) | 0.2722 |  | 126.51(62.20-257.31) | 104.94(51.40-214.22) | 0.7113 |
| Post-booster antibody GMC (95%CI) | 4589.39(3975.87-5297.59) | 464.48(401.67-537.11) |  |  | 5896.06(3795.35-9159.52) | 516.06(333.55-798.45) |  |
| Post-booster antibody GMC adjusted between NVSI-06-07 and BBIBP-CorV groups (95%CI) | 4676.67(4079.72-5360.95) | 455.90(397.97 -522.26) |  |  | 5713.06(3839.73-8500.35) | 529.68(369.13-760.06) |  |
| Ratio of adjusted GMC between NVSI-06-07 and BBIBP-CorV groups (95% CI)^b^ | 10.26(8.46-12.44) |  | <0.0001^c^ |  | 10.79(6.30-18.46) |  | <0.0001^c^ |
| Post-booster antibody GMC adjusted between different age subgroups (95%CI) | 4600.87(3975.94-5324.03) |  |  |  | 5817.89(4151.52-8153.13) |  |  |
| Ratio of adjusted GMC between two age subgroups (95%CI)^d^ | 0.79(0.55-1.14) |  |  |  | P=0.2099 |  |  |
| Rate of seroconversion^e^, n (%) | 170(83.74) | 57(27.80) | <0.0001 |  | 33(86.84) | 17(36.96) | <0.0001 |
| 95%CI (%) | 77.93-88.54 | 21.79-34.47 |  |  | 71.91-95.59 | 23.21-52.45 |  |
| Rate difference between NVSI-06-07 and BBIBP-CorV groups (%, 95%CI)^f^ | 55.94(47.98-63.90) |  |  |  | 49.89(32.28-67.49) |  |  |
| Post-booster antibody GMC fold rise (95%CI) | 40.82(30.36-54.88) | 3.32(2.62-4.21) | <0.0001 |  | 46.61(26.75-81.19) | 4.92(2.53-9.54) | <0.0001 |

^a^GMC represents geometric mean concentration, and the unit of GMC is BAU/ml

^b^The ratio of adjusted GMC between two groups was calculated by “NVSI-06-07/ BBIBP-CorV”

^c^Covariance analysis with least square method was used to calculate the adjusted GMC and P value

^d^The ratio of adjusted GMC between two age subgroups was calculated by “<45 years old / ≥45 years old”

^e^Seroconversion was defined as more than 4-fold rise form baseline in IgG concentration

^f^Rate difference=(NVSI-06-07)-(BBIBP-CorV). Rate difference and 95%CI were estimated by CMH method considering stratification factors

**Table S6:** RBD-specific IgG Response Results for different age subgroups (28 days after boosting) (PPS2) ( >=45 years old)

|  | <60 years old | | |  | >=60 years old | | |
| --- | --- | --- | --- | --- | --- | --- | --- |
|  | NVSI-06-07 | BBIBP-CorV | P value |  | NVSI-06-07 | BBIBP-CorV | P value |
| 1-3 months |  |  |  |  |  |  |  |
| N(missing) | 19(0) | 19(0) |  |  | 5(0) | 6(0) |  |
| Pre-booster antibody GMC^a^ (95%CI) | 102.06(47.20-220.69) | 191.78(82.26-447.11) | 0.2548 |  | 250.36(13.17-4761.05) | 95.45(20.60-442.23) | 0.4283 |
| Post-booster antibody GMC (95%CI) | 2287.49(1034.87-5056.30) | 407.02(261.34-633.91) |  |  | 4032.29(481.00-33803.4) | 226.08(92.84-550.56) |  |
| Post-booster antibody GMC adjusted between NVSI-06-07 and BBIBP-CorV groups (95%CI) | 2712.15(1702.61-4320.30) | 343.29(215.51-546.84) |  |  | 2920.70(1557.95-5475.46) | 295.79(166.98-523.97) |  |
| Ratio of adjusted GMC between NVSI-06-07 and BBIBP-CorV groups (95% CI)^b^ | 7.90(4.07-15.35) |  | <0.0001^c^ |  | 9.87(4.16-23.45) |  | 0.0003^c^ |
| Post-booster antibody GMC adjusted between different age subgroups (95%CI) | 2608.17(1519.08-4478.08) |  |  |  | 2449.29(842.01-7124.70) |  |  |
| Ratio of adjusted GMC between two age subgroups (95%CI)^d^ | 1.06(0.32-3.56) |  |  |  | P=0.9147 |  |  |
| Rate of seroconversion^e^, n (%) | 18(94.74) | 3(15.79) | <0.0001 |  | 4(80.00) | 2(33.33) | 0.2424 |
| 95%CI (%) | 73.97-99.87 | 3.38-39.58 |  |  | 28.36-99.49 | 4.33-77.72 |  |
| Rate difference between NVSI-06-07 and BBIBP-CorV groups (%, 95%CI)^f^ | 78.95(59.72-98.17) |  |  |  | 46.67(-4.83-98.16) |  |  |
| Post-booster antibody GMC fold rise (95%CI) | 22.41(12.38-40.56) | 2.12(1.16-3.90) | <0.0001 |  | 16.11(3.74-69.38) | 2.37(1.17-4.81) | 0.0079 |
| 4-6 months |  |  |  |  |  |  |  |
| N(missing) | 23(0) | 29(0) |  |  | 1(0) | 3(0) |  |
| Pre-booster antibody GMC^a^ (95%CI) | 259.61(133.59-504.50) | 131.51(75.87-227.94) | 0.1076 |  | 518.50 | 58.53(0.30-11239.70) | -^g^ |
| Post-booster antibody GMC (95%CI) | 4446.07(2556.94-7730.91) | 357.43(251.60-507.77) |  |  | 3420.00 | 435.28(19.96-9493.91) |  |
| Post-booster antibody GMC adjusted between NVSI-06-07 and BBIBP-CorV groups (95%CI) | 4044.33(2599.09-6293.22) | 385.31(260.20-570.56) |  |  | 2481.62 | 484.39 |  |
| Ratio of adjusted GMC between NVSI-06-07 and BBIBP-CorV groups (95% CI)^b^ | 10.50(5.77-19.20) |  | <0.0001^c^ |  | 5.12 |  | -^g^ |
| Post-booster antibody GMC adjusted between different age subgroups (95%CI) | 4490.89(2680.88-7522.94) |  |  |  | 2715.32(226.44-32560.50) |  |  |
| Ratio of adjusted GMC between two age subgroups (95%CI)^d^ | 1.65(0.13-20.93) |  |  |  | P=0.6843 |  |  |
| Rate of seroconversion^e^, n (%) | 20(86.96) | 5(17.24) | <0.0001 |  | 1(100.00) | 1(33.33) | -^g^ |
| 95%CI (%) | 66.41-97.22 | 5.85-35.77 |  |  | 2.50-100.00 | 0.84-90.57 |  |
| Rate difference between NVSI-06-07 and BBIBP-CorV groups (%, 95%CI)^f^ | 69.72(50.26-89.17) |  |  |  | 66.67(13.32-100.00) |  |  |
| Post-booster antibody GMC fold rise (95%CI) | 17.13(8.82-33.25) | 2.72(1.53-4.82) | <0.0001 |  | 6.60 | 7.44(0.04-1255.63) | -^g^ |
| ≥6 months |  |  |  |  |  |  |  |
| N(missing) | 34(0) | 41(0) |  |  | 4(0) | 5(0) |  |
| Pre-booster antibody GMC^a^ (95%CI) | 143.09(67.55-303.10) | 133.31(63.05-281.85) | 0.8936 |  | 44.41(1.26-1561.49) | 14.75(1.54-140.76) | 0.4400 |
| Post-booster antibody GMC (95%CI) | 5982.19(3777.41-9473.87) | 521.18(321.13-845.84) |  |  | 5212.26(326.50-83209.0) | 475.97(161.88-1399.48) |  |
| Post-booster antibody GMC adjusted between NVSI-06-07 and BBIBP-CorV groups (95%CI) | 5906.29(3868.11-9018.43) | 526.73(358.26-774.42) |  |  | 4467.07(860.72-23183.89) | 538.50(124.38-2331.35) |  |
| Ratio of adjusted GMC between NVSI-06-07 and BBIBP-CorV groups (95% CI)^b^ | 11.21(6.33-19.88) |  | <0.0001^c^ |  | 8.30(0.87-78.98) |  | 0.0613^c^ |
| Post-booster antibody GMC adjusted between different age subgroups (95%CI) | 5698.35(3920.49-8282.43) |  |  |  | 7879.04(2615.55 -23734.72) |  |  |
| Ratio of adjusted GMC between two age subgroups (95%CI)^d^ | 0.72(0.23-2.32) |  |  |  | P=0.5767 |  |  |
| Rate of seroconversion^e^, n (%) | 29(85.29) | 13(31.71) | <0.0001 |  | 4(100.00) | 4(80.00) | 1.0000 |
| 95%CI (%) | 68.94-95.05 | 18.08-48.09 |  |  | 39.76-100.00 | 28.36-99.49 |  |
| Rate difference between NVSI-06-07 and BBIBP-CorV groups (%, 95%CI)^f^ | 53.59(35.02-72.15) |  |  |  | 20.00(-15.06-55.06) |  |  |
| Post-booster antibody GMC fold rise (95%CI) | 41.81(22.74-76.87) | 3.91(2.00-7.63) | <0.0001 |  | 117.36(44.01-312.96） | 32.28(1.45-718.37） | 0.3523 |

^a^GMC represents geometric mean concentration, and the unit of GMC is BAU/ml

^b^The ratio of adjusted GMC between two groups was calculated by “NVSI-06-07/ BBIBP-CorV”

^c^Covariance analysis with least square method was used to calculate the adjusted GMC and P value

^d^The ratio of adjusted GMC between two age subgroups was calculated by “<45 years old / ≥45 years old”

^e^Seroconversion was defined as more than 4-fold rise form baseline in IgG concentration

^f^Rate difference=(NVSI-06-07)-(BBIBP-CorV). Rate difference and 95%CI were estimated by CMH method considering stratification factors

^g^Due to only one participant in the subgroup, P value was not calculated

**Table S7:** Neutralizing antibody response results for different age subgroups (14 days after boosting) (PPS1)

|  | <45 years old | | |  | >=45 years old | | |
| --- | --- | --- | --- | --- | --- | --- | --- |
|  | NVSI-06-07 | BBIBP-CorV | P value |  | NVSI-06-07 | BBIBP-CorV | P value |
| 1-3 months |  |  |  |  |  |  |  |
| N(missing) | 243 (0) | 243 (0) |  |  | 24 (0) | 27 (0) |  |
| Pre-booster antibody GMT^a^ (95%CI) | 97.85 (83.44-114.75) | 84.78 (71.83-100.06) | 0.2198 |  | 76.53 (38.59-151.77) | 108.88 (54.19-218.77) | 0.4628 |
| Post-booster antibody GMT (95%CI) | 1398.22 (1204.09-1623.65) | 297.44 (265.95-332.65) |  |  | 838.64 (441.76-1592.06) | 285.30 (195.90-415.50) |  |
| Post-booster antibody GMT adjusted between NVSI-06-07 and BBIBP-CorV groups (95%CI) | 1366.72 (1210.45-1543.15) | 304.29 (269.50-343.57) |  |  | 922.05 (633.19-1342.69) | 262.24 (184.02-373.71) |  |
| Ratio of adjusted GMT between NVSI-06-07 and BBIBP-CorV groups (95% CI)^b^ | 4.49 (3.78-5.33) |  | <0.0001^c^ |  | 3.52 (2.09-5.90) |  | <0.0001^c^ |
| Post-booster antibody GMT adjusted between different age subgroups (95%CI) | 1383.44 (1212.26-1578.79) |  |  |  | 933.90 (613.12-1422.51) |  |  |
| Ratio of adjusted GMT between two age subgroups (95%CI)^d^ | 1.48 (0.95-2.30) |  |  |  | P=0.0806 |  |  |
| Rate of seroconversion^e^, n (%) | 198 (81.48) | 99 (40.74) | <0.0001 |  | 20 (83.33) | 7 (25.93) | <0.0001 |
| 95%CI (%) | 76.02-86.16 | 34.50-47.21 |  |  | 62.62-95.26 | 11.11-46.28 |  |
| Rate difference between NVSI-06-07 and BBIBP-CorV groups (%, 95%CI)^f^ | 40.74 (32.87-48.62) |  |  |  | 57.41 (35.15-79.67) |  |  |
| Post-booster antibody GMT fold rise (95%CI) | 14.29 (12.19-16.75) | 3.51 (2.96-4.16) | <0.0001 |  | 10.96 (6.92-17.36) | 2.62 (1.54-4.46) | 0.0001 |
| 4-6 months |  |  |  |  |  |  |  |
| N(missing) | 254 (0) | 254 (0) |  |  | 25 (0) | 36 (0) |  |
| Pre-booster antibody GMT^a^ (95%CI) | 107.38 (90.51-127.39) | 130.93 (111.39-153.90) | 0.0975 |  | 143.77 (84.11-245.73) | 103.61 (59.04-181.81) | 0.4126 |
| Post-booster antibody GMT (95%CI) | 1830.38 (1608.85-2082.42) | 321.72 (290.58-356.21) |  |  | 1643.68 (1100.46-2455.05) | 374.11 (276.53-506.12) |  |
| Post-booster antibody GMT adjusted between NVSI-06-07 and BBIBP-CorV groups (95%CI) | 1881.07 (1690.54-2093.08) | 313.06 (281.35-348.34) |  |  | 1563.54 (1113.43-2195.61) | 387.33 (291.96-513.83) |  |
| Ratio of adjusted GMT between NVSI-06-07 and BBIBP-CorV groups (95% CI)^b^ | 6.01 (5.17-6.99) |  | <0.0001^c^ |  | 4.04 (2.59-6.29) |  | <0.0001^c^ |
| Post-booster antibody GMT adjusted between different age subgroups (95%CI) | 1846.44 (1645.56-2071.85) |  |  |  | 1504.09 (1041.33-2172.51) |  |  |
| Ratio of adjusted GMT between two age subgroups (95%CI)^d^ | 1.23 (0.83-1.80) |  |  |  | P=0.2958 |  |  |
| Rate of seroconversion^e^, n (%) | 220 (86.61) | 65 (25.59) | <0.0001 |  | 21 (84.00) | 13 (36.11) | 0.0002 |
| 95%CI (%) | 81.80-90.55 | 20.34-31.42 |  |  | 63.92-95.46 | 20.82-53.78 |  |
| Rate difference between NVSI-06-07 and BBIBP-CorV groups (%, 95%CI)^f^ | 61.02 (54.22-67.83) |  |  |  | 47.89 (26.61-69.17) |  |  |
| Post-booster antibody GMT fold rise (95%CI) | 17.05 (14.49-20.05) | 2.46 (2.09-2.88) | <0.0001 |  | 11.43 (6.88-19.01) | 3.61 (2.16-6.03) | 0.0026 |
| ≥6 months |  |  |  |  |  |  |  |
| N(missing) | 238 (0) | 237 (0) |  |  | 43 (0) | 48 (0) |  |
| Pre-booster antibody GMT^a^ (95%CI) | 55.22 (44.28-68.85) | 63.31 (51.34-78.07) | 0.3768 |  | 43.14 (24.11-77.18) | 51.13 (30.04-87.04) | 0.6644 |
| Post-booster antibody GMT (95%CI) | 1863.72 (1603.20-2166.57) | 440.79 (397.14-489.23) |  |  | 2162.41 (1386.06-3373.59) | 493.79 (362.63-672.38) |  |
| Post-booster antibody GMT adjusted between NVSI-06-07 and BBIBP-CorV groups (95%CI) | 1889.62 (1672.93-2134.38) | 434.72 (384.77-491.15) |  |  | 2237.83 (1630.78-3070.84) | 478.85 (354.92-646.06) |  |
| Ratio of adjusted GMT between NVSI-06-07 and BBIBP-CorV groups (95% CI)^b^ | 4.35 (3.66-5.17) |  | <0.0001^c^ |  | 4.67 (3.02-7.23) |  | <0.0001^c^ |
| Post-booster antibody GMT adjusted between different age subgroups (95%CI) | 1843.68 (1598.69-2126.21) |  |  |  | 2295.78 (1641.05-3211.73) |  |  |
| Ratio of adjusted GMT between two age subgroups (95%CI)^d^ | 0.80 (0.56-1.16) |  |  |  | P=0.2378 |  |  |
| Rate of seroconversion^e^, n (%) | 203 (85.29) | 124 (52.32) | <0.0001 |  | 41 (95.35) | 27 (56.25) | <0.0001 |
| 95%CI (%) | 80.15-89.54 | 45.76-58.83 |  |  | 84.19-99.43 | 41.18-70.52 |  |
| Rate difference between NVSI-06-07 and BBIBP-CorV groups (%, 95%CI)^f^ | 32.97 (25.18-40.76) |  |  |  | 39.10 (23.72-54.48) |  |  |
| Post-booster antibody GMT fold rise (95%CI) | 33.75 (27.10-42.03) | 6.96 (5.69-8.52) | <0.0001 |  | 50.13 (32.45-77.44) | 9.66 (5.97-15.63) | <0.0001 |

^a^GMT represent geometric mean titer

^b^The ratio of adjusted GMT between two groups was calculated by “NVSI-06-07/ BBIBP-CorV”, and the non-inferiority threshold of ratio between groups was set to 0.67

^c^Covariance analysis with least square method was used to calculate the adjusted GMT and P value

^d^The ratio of adjusted GMT between two age subgroups was calculated by “<45 years old / ≥45 years old”

^e^Seroconversion was defined as more than 4-fold rise form baseline in neutralizing antibody titer

^f^Rate difference=(NVSI-06-07)-(BBIBP-CorV). Rate difference and 95%CI were estimated by CMH method considering stratification factors

**Table S8:** Neutralizing antibody response results for different age subgroups (14 days after boosting) (PPS1) ( >=45 years old)

|  | 45-59 years old | | |  | >59 years old | | |
| --- | --- | --- | --- | --- | --- | --- | --- |
|  | NVSI-06-07 | BBIBP-CorV | P value |  | NVSI-06-07 | BBIBP-CorV | P value |
| 1-3 months |  |  |  |  |  |  |  |
| N(missing) | 19 (0) | 20 (0) |  |  | 5 (0) | 7 (0) |  |
| Pre-booster antibody GMT^a^ (95%CI) | 68.89 (33.23-142.83) | 118.50 (52.82-265.85) | 0.3046 |  | 114.09 (8.02-1622.37) | 85.50 (13.61-537.23) | 0.8148 |
| Post-booster antibody GMT (95%CI) | 714.53 (341.68-1494.25) | 311.14 (205.27-471.60) |  |  | 1541.26 (252.51-9407.51) | 222.71 (77.08-643.47) |  |
| Post-booster antibody GMT adjusted between NVSI-06-07 and BBIBP-CorV groups (95%CI) | 811.37 (505.29-1302.86) | 275.75 (173.83-437.43) |  |  | 1394.33 (921.42-2109.97) | 239.24 (168.60-339.47) |  |
| Ratio of adjusted GMT between NVSI-06-07 and BBIBP-CorV groups (95% CI)^b^ | 2.94 (1.51-5.73) |  | 0.0023^c^ |  | 5.83 (3.39-10.03) |  | <0.0001^c^ |
| Post-booster antibody GMT adjusted between different age subgroups (95%CI) | 768.96 (473.37-1249.13) |  |  |  | 1166.08 (450.79-3016.37) |  |  |
| Ratio of adjusted GMT between two age subgroups (95%CI)^d^ | 0.66 (0.23-1.92) |  |  |  | P=0.4275 |  |  |
| Rate of seroconversion^e^, n (%) | 15 (78.95) | 5 (25.00) | 0.0009 |  | 5 (100.00) | 2 (28.57) | 0.0178 |
| 95%CI (%) | 54.43-93.95 | 8.66-49.10 |  |  | 47.82-100.00 | 3.67-70.96 |  |
| Rate difference between NVSI-06-07 and BBIBP-CorV groups (%, 95%CI)^f^ | 53.95 (27.56-80.33) |  |  |  | 71.43 (37.96-100.00) |  |  |
| Post-booster antibody GMT fold rise (95%CI) | 10.37 (5.95-18.07) | 2.63 (1.31-5.25) | 0.0027 |  | 13.51 (4.47-40.80) | 2.60 (1.10-6.17) | 0.0118 |
| 4-6 months |  |  |  |  |  |  |  |
| N(missing) | 24 (0) | 33 (0) |  |  | 1 (0) | 3 (0) |  |
| Pre-booster antibody GMT^a^ (95%CI) | 157.54 (93.26-266.11) | 106.92 (60.38-189.36) | 0.3295 |  | 16.00 | 73.26 (0.13-42918.9) | -^g^ |
| Post-booster antibody GMT (95%CI) | 1680.92 (1108.36-2549.26) | 370.25 (268.94-509.74) |  |  | 960.00 | 419.32 (38.01-4626.14) |  |
| Post-booster antibody GMT adjusted between NVSI-06-07 and BBIBP-CorV groups (95%CI) | 1584.84 (1111.23-2260.30) | 386.45 (285.62-522.87) |  |  | 1272.92 | 381.68 |  |
| Ratio of adjusted GMT between NVSI-06-07 and BBIBP-CorV groups (95% CI)^b^ | 4.10 (2.57-6.55) |  | <0.0001^c^ |  | 3.34 |  | -^g^ |
| Post-booster antibody GMT adjusted between different age subgroups (95%CI) | 1629.11 (1107.05-2397.36) |  |  |  | 2035.24 (272.28-15212.97) |  |  |
| Ratio of adjusted GMT between two age subgroups (95%CI)^d^ | 0.80 (0.10-6.27) |  |  |  | P=0.8246 |  |  |
| Rate of seroconversion^e^, n (%) | 20 (83.33) | 12 (36.36) | 0.0005 |  | 1 (100.00) | 1 (33.33) | -^g^ |
| 95%CI (%) | 62.62-95.26 | 20.40-54.88 |  |  | 2.50-100.00 | 0.84-90.57 |  |
| Rate difference between NVSI-06-07 and BBIBP-CorV groups (%, 95%CI)^f^ | 46.97 (24.80-69.14) |  |  |  | 66.67 (13.32-100.00) |  |  |
| Post-booster antibody GMT fold rise (95%CI) | 10.67 (6.41-17.77) | 3.46 (2.04-5.88) | 0.0036 |  | 60.00 | 5.72 (0.03-965.33) | -^g^ |
| ≥6 months |  |  |  |  |  |  |  |
| N(missing) | 39 (0) | 43 (0) |  |  | 4 (0) | 5 (0) |  |
| Pre-booster antibody GMT^a^ (95%CI) | 48.61 (26.85-88.01) | 60.85 (35.02-105.74) | 0.5769 |  | 13.45 (0.32-574.28) | 11.45 (1.63-80.39) | 0.9051 |
| Post-booster antibody GMT (95%CI) | 2195.56 (1378.25-3497.53) | 517.91 (368.97-726.97) |  |  | 1864.29 (114.58-30331.9) | 327.63 (165.72-647.72) |  |
| Post-booster antibody GMT adjusted between NVSI-06-07 and BBIBP-CorV groups (95%CI) | 2297.43 (1633.17-3231.86) | 497.04 (359.13-687.90) |  |  | 1788.54 (601.88-5314.83) | 338.68 (127.88-897.01) |  |
| Ratio of adjusted GMT between NVSI-06-07 and BBIBP-CorV groups (95% CI)^b^ | 4.62 (2.88-7.41) |  | <0.0001^c^ |  | 5.28 (1.22-22.78) |  | 0.0318^c^ |
| Post-booster antibody GMT adjusted between different age subgroups (95%CI) | 2061.11 (1448.51-2932.80) |  |  |  | 3452.13 (1126.66-10577.50) |  |  |
| Ratio of adjusted GMT between two age subgroups (95%CI)^d^ | 0.60 (0.18-1.94) |  |  |  | P=0.3815 |  |  |
| Rate of seroconversion^e^, n (%) | 37 (94.87) | 23 (53.49) | <0.0001 |  | 4 (100.00) | 4 (80.00) | 0.3711 |
| 95%CI (%) | 82.68-99.37 | 37.65-68.82 |  |  | 39.76-100.00 | 28.36-99.49 |  |
| Rate difference between NVSI-06-07 and BBIBP-CorV groups (%, 95%CI)^f^ | 41.38 (24.95-57.82) |  |  |  | 20.00 (-15.06-55.06) |  |  |
| Post-booster antibody GMT fold rise (95%CI) | 45.16 (28.39-71.83) | 8.51 (5.15-14.06) | <0.0001 |  | 138.56 (45.97-417.68) | 28.62 (3.66-223.98) | 0.1210 |

^a^GMT represent geometric mean titer

^b^The ratio of adjusted GMT between two groups was calculated by “NVSI-06-07/ BBIBP-CorV”, and the non-inferiority threshold of ratio between groups was set to 0.67

^c^Covariance analysis with least square method was used to calculate the adjusted GMT and P value

^d^The ratio of adjusted GMT between two age subgroups was calculated by “<45 years old / ≥45 years old”

^e^Seroconversion was defined as more than 4-fold rise form baseline in neutralizing antibody titer

^f^Rate difference=(NVSI-06-07)-(BBIBP-CorV). Rate difference and 95%CI were estimated by CMH method considering stratification factors

^g^Due to only one participant in the subgroup, P value was not calculated

**Table S9:** Neutralizing antibody response results for different age subgroups (28days after boosting) (PPS2)

|  | <45 years old | | |  | >=45 years old | | |
| --- | --- | --- | --- | --- | --- | --- | --- |
|  | NVSI-06-07 | BBIBP-CorV | P value |  | NVSI-06-07 | BBIBP-CorV | P value |
| 1-3 months |  |  |  |  |  |  |  |
| N(missing) | 232 (0) | 222 (0) |  |  | 24 (0) | 25 (0) |  |
| Pre-booster antibody GMT^a^ (95%CI) | 95.67 (80.94-113.09) | 80.48 (67.66-95.73) | 0.1579 |  | 74.35 (37.26-148.37) | 117.60 (55.55-248.98) | 0.3587 |
| Post-booster antibody GMT (95%CI) | 2006.05 (1742.63-2309.28) | 596.30 (530.26-670.55) |  |  | 1594.38 (932.73-2725.40) | 556.29 (355.76-869.85) |  |
| Post-booster antibody GMT adjusted between NVSI-06-07 and BBIBP-CorV groups (95%CI) | 1961.24 (1738.50-2212.50) | 610.54 (539.75-690.62) |  |  | 1733.97 (1141.91-2632.99) | 513.23 (340.88-772.72) |  |
| Ratio of adjusted GMT between NVSI-06-07 and BBIBP-CorV groups (95% CI)^b^ | 3.21 (2.70-3.82) |  | <0.0001^c^ |  | 3.38 (1.88-6.08) |  | 0.0001^c^ |
| Post-booster antibody GMT adjusted between different age subgroups (95%CI) | 1987.21 (1752.35-2253.54) |  |  |  | 1746.63 (1180.75-2583.69) |  |  |
| Ratio of adjusted GMT between two age subgroups (95%CI)^d^ | 1.14 (0.75-1.72) |  |  |  | P=0.5372 |  |  |
| Rate of seroconversion^e^, n (%) | 211 (90.95) | 137 (61.71) | <0.0001 |  | 21 (87.50) | 11(44.00) | 0.0016 |
| 95%CI (%) | 86.50-94.31 | 54.97-68.14 |  |  | 67.64-97.34 | 24.40-65.07 |  |
| Rate difference between NVSI-06-07 and BBIBP-CorV groups (%, 95%CI)^f^ | 29.24 (21.85-36.62) |  |  |  | 43.50 (19.97-67.03) |  |  |
| Post-booster antibody GMT fold rise (95%CI) | 20.97 (17.90-24.56) | 7.41 (6.12-8.97) | <0.0001 |  | 21.44 (11.10-41.44) | 4.73 (2.62-8.53) | 0.0009 |
| 4-6 months |  |  |  |  |  |  |  |
| N(missing) | 225 (0) | 218 (0) |  |  | 24 (0) | 32 (0) |  |
| Pre-booster antibody GMT^a^ (95%CI) | 107.67 (89.51-129.52) | 145.79 (123.13-172.62) | 0.0176 |  | 127.17 (69.84-231.56) | 96.27 (52.21-177.52) | 0.5182 |
| Post-booster antibody GMT (95%CI) | 2599.18 (2266.83-2980.26) | 573.19 (507.74-647.08) |  |  | 1942.06 (1153.99-3268.30) | 634.54 (457.55-880.00) |  |
| Post-booster antibody GMT adjusted between NVSI-06-07 and BBIBP-CorV groups (95%CI) | 2719.96 (2419.62-3057.58) | 546.94 (485.64-615.99) |  |  | 1886.23 (1237.62-2874.75) | 648.57 (450.36-934.03) |  |
| Ratio of adjusted GMT between NVSI-06-07 and BBIBP-CorV groups (95% CI)^b^ | 4.97 (4.21-5.88) |  | <0.0001^c^ |  | 2.91 (1.66-5.08) |  | 0.0003^c^ |
| Post-booster antibody GMT adjusted between different age subgroups (95%CI) | 2611.99 (2299.27-2967.24) |  |  |  | 1854.62 (1254.88-2740.99) |  |  |
| Ratio of adjusted GMT between two age subgroups (95%CI)^d^ | 1.41 (0.93-2.12) |  |  |  | P=0.1020 |  |  |
| Rate of seroconversion^e^, n (%) | 205 (91.11) | 80 (36.70) | <0.0001 |  | 19 (79.17) | 12 (37.50) | 0.0021 |
| 95%CI (%) | 86.61-94.49 | 30.29-43.47 |  |  | 57.85-92.87 | 21.10-56.31 |  |
| Rate difference between NVSI-06-07 and BBIBP-CorV groups (%, 95%CI)^f^ | 54.41 (47.01-61.81) |  |  |  | 41.67 (18.31-65.02) |  |  |
| Post-booster antibody GMT fold rise (95%CI) | 24.14 (20.16-28.91) | 3.93 (3.35-4.61) | <0.0001 |  | 15.27 (8.46-27.56) | 6.59 (3.43-12.65) | 0.0641 |
| ≥6 months |  |  |  |  |  |  |  |
| N(missing) | 204 (0) | 205 (0) |  |  | 38 (0) | 47 (0) |  |
| Pre-booster antibody GMT^a^ (95%CI) | 60.11 (47.91-75.42) | 68.79 (55.47-85.30) | 0.3958 |  | 53.68 (29.50-97.68) | 50.45 (29.31-86.83) | 0.8773 |
| Post-booster antibody GMT (95%CI) | 3631.61 (3128.23-4216.00) | 1073.50 (937.45-1229.29) |  |  | 4613.07 (2888.89-7366.30) | 1160.59 (823.94-1634.79) |  |
| Post-booster antibody GMT adjusted between NVSI-06-07 and BBIBP-CorV groups (95%CI) | 3672.66 (3200.36-4214.67) | 1061.56 (925.36-1217.81) |  |  | 4562.40 (3146.93-6614.54) | 1171.00 (838.53-1635.29) |  |
| Ratio of adjusted GMT between NVSI-06-07 and BBIBP-CorV groups (95% CI)^b^ | 3.46 (2.85-4.20) |  | <0.0001^c^ |  | 3.90 (2.36-6.42) |  | <0.0001^c^ |
| Post-booster antibody GMT adjusted between different age subgroups (95%CI) | 3613.81 (3128.84-4173.96) |  |  |  | 4736.38 (3391.67-6614.24) |  |  |
| Ratio of adjusted GMT between two age subgroups (95%CI)^d^ | 0.76 (0.53-1.10) |  |  |  | P=0.1442 |  |  |
| Rate of seroconversion^e^, n (%) | 198 (97.06) | 168 (81.95) | <0.0001 |  | 38 (100.00) | 38 (80.85) | 0.0046 |
| 95%CI (%) | 93.71-98.91 | 75.99-86.96 |  |  | 90.75-100.00 | 66.74-90.85 |  |
| Rate difference between NVSI-06-07 and BBIBP-CorV groups (%, 95%CI)^f^ | 15.11 (9.35-20.86) |  |  |  | 19.15 (7.90-30.40) |  |  |
| Post-booster antibody GMT fold rise (95%CI) | 60.41 (48.15-75.80) | 15.61 (12.36-19.70) | <0.0001 |  | 85.94 (55.76-132.44) | 23.00 (13.01-40.67) | 0.0004 |

^a^GMT represent geometric mean titer

^b^The ratio of adjusted GMT between two groups was calculated by “NVSI-06-07/ BBIBP-CorV”, and the non-inferiority threshold of ratio between groups was set to 0.67

^c^Covariance analysis with least square method was used to calculate the adjusted GMT and P value

^d^The ratio of adjusted GMT between two age subgroups was calculated by “<45 years old / ≥45 years old”

^e^Seroconversion was defined as more than 4-fold rise form baseline in neutralizing antibody titer

^f^Rate difference=(NVSI-06-07)-(BBIBP-CorV). Rate difference and 95%CI were estimated by CMH method considering stratification factors

**Table S10:** Neutralizing antibody response results for different age subgroups (28 days after boosting) (PPS2) ( >=45 years old)

|  | 45-59 years old | | |  | >59 years old | | |
| --- | --- | --- | --- | --- | --- | --- | --- |
|  | NVSI-06-07 | BBIBP-CorV | P value |  | NVSI-06-07 | BBIBP-CorV | P value |
| 1-3 months |  |  |  |  |  |  |  |
| N(missing) | 19 (0) | 19 (0) |  |  | 5 (0) | 6 (0) |  |
| Pre-booster antibody GMT^a^ (95%CI) | 66.43 (31.77-138.87) | 140.76 (60.33-328.43) | 0.1687 |  | 114.09 (8.02-1622.37) | 66.56 (7.72-573.68) | 0.6804 |
| Post-booster antibody GMT (95%CI) | 1560.68 (866.44-2811.18) | 617.51 (372.88-1022.63) |  |  | 1729.23 (238.06-12560.9) | 399.69 (109.89-1453.69) |  |
| Post-booster antibody GMT adjusted between NVSI-06-07 and BBIBP-CorV groups (95%CI) | 1734.80 (1055.38-2851.61) | 555.54 (337.96-913.17) |  |  | 1475.16 (589.18-3693.45) | 456.28 (197.56-1053.82) |  |
| Ratio of adjusted GMT between NVSI-06-07 and BBIBP-CorV groups (95% CI)^b^ | 3.12 (1.53-6.37) |  | 0.0026^c^ |  | 3.23 (0.93-11.27) |  | 0.0620^c^ |
| Post-booster antibody GMT adjusted between different age subgroups (95%CI) | 1623.40 (919.91-2864.88) |  |  |  | 1488.77 (488.98-4532.82) |  |  |
| Ratio of adjusted GMT between two age subgroups (95%CI)^d^ | 1.09 (0.31-3.82) |  |  |  | P=0.8872 |  |  |
| Rate of seroconversion^e^, n (%) | 16 (84.21) | 7 (36.84) | 0.0032 |  | 5 (100.00) | 4 (66.67) | 0.1736 |
| 95%CI (%) | 60.42-96.62 | 16.29-61.64 |  |  | 47.82-100.00 | 22.28-95.67 |  |
| Rate difference between NVSI-06-07 and BBIBP-CorV groups (%, 95%CI)^f^ | 47.37 (20.18-74.56) |  |  |  | 33.33 (-4.39-71.05) |  |  |
| Post-booster antibody GMT fold rise (95%CI) | 23.50 (10.68-51.68) | 4.39 (2.12-9.09) | 0.0023 |  | 15.16 (2.91-78.98) | 6.00 (1.66-21.71) | 0.2601 |
| 4-6 months |  |  |  |  |  |  |  |
| N(missing) | 23 (0) | 29 (0) |  |  | 1 (0) | 3 (0) |  |
| Pre-booster antibody GMT^a^ (95%CI) | 139.16 (76.65-252.66) | 99.03 (52.94-185.23) | 0.4309 |  | 16.00 | 73.26 (0.13-42918.9) | -^g^ |
| Post-booster antibody GMT (95%CI) | 1943.02 (1126.60-3351.07) | 640.30 (456.54-898.02) |  |  | 1920.00 | 581.48 (23.50-14388.8) |  |
| Post-booster antibody GMT adjusted between NVSI-06-07 and BBIBP-CorV groups (95%CI) | 1881.73 (1211.50-2922.75) | 656.78 (443.85-971.86) |  |  | 2930.91 | 505.01 |  |
| Ratio of adjusted GMT between NVSI-06-07 and BBIBP-CorV groups (95% CI)^b^ | 2.87 (1.59-5.18) |  | 0.0008^c^ |  | 5.80 |  | -^g^ |
| Post-booster antibody GMT adjusted between different age subgroups (95%CI) | 1868.64 (1140.55-3061.51) |  |  |  | 4711.97 (394.30-56308.86) |  |  |
| Ratio of adjusted GMT between two age subgroups (95%CI)^d^ | 0.40 (0.03-5.02) |  |  |  | P=0.4572 |  |  |
| Rate of seroconversion^e^, n (%) | 18 (78.26) | 10 (34.48) | 0.0018 |  | 1 (100.00) | 2 (66.67) | -^g^ |
| 95%CI (%) | 56.30-92.54 | 17.94-54.33 |  |  | 2.50-100.00 | 9.43-99.16 |  |
| Rate difference between NVSI-06-07 and BBIBP-CorV groups (%, 95%CI)^f^ | 43.78 (19.62-67.93) |  |  |  | 33.33 (-20.01-86.68) |  |  |
| Post-booster antibody GMT fold rise (95%CI) | 13.96 (7.76-25.11) | 6.47 (3.21-13.00) | 0.0996 |  | 120.00 | 7.94 (0.08-759.14) | -^g^ |
| ≥6 months |  |  |  |  |  |  |  |
| N(missing) | 34 (0) | 42 (0) |  |  | 4 (0) | 5 (0) |  |
| Pre-booster antibody GMT^a^ (95%CI) | 63.17 (34.68-115.07) | 60.19 (34.18-105.99) | 0.9064 |  | 13.45 (0.32-574.28) | 11.45 (1.63-80.39) | 0.9051 |
| Post-booster antibody GMT (95%CI) | 4690.21 (2800.11-7856.12) | 1230.38 (846.63-1788.09) |  |  | 4006.62 (859.73-18672.1) | 710.61 (278.37-1813.96) |  |
| Post-booster antibody GMT adjusted between NVSI-06-07 and BBIBP-CorV groups (95%CI) | 4645.69 (3105.38-6950.00) | 1239.92 (862.98-1781.50) |  |  | 3979.03 (1312.07-12066.94) | 714.54 (264.93-1927.22) |  |
| Ratio of adjusted GMT between NVSI-06-07 and BBIBP-CorV groups (95% CI)^b^ | 3.75 (2.18-6.44) |  | <0.0001^c^ |  | 5.57 (1.26-24.69) |  | 0.0303^c^ |
| Post-booster antibody GMT adjusted between different age subgroups (95%CI) | 4269.17 (2983.17-6109.54) |  |  |  | 8912.38 (3041.04-26119.52) |  |  |
| Ratio of adjusted GMT between two age subgroups (95%CI)^d^ | 0.48 (0.15-1.50) |  |  |  | P=0.1988 |  |  |
| Rate of seroconversion^e^, n (%) | 34 (100.00) | 34 (80.95) | 0.0075 |  | 4 (100.00) | 4 (80.00) | 0.3711 |
| 95%CI (%) | 89.72-100.00 | 65.88-91.40 |  |  | 39.76-100.00 | 28.36-99.49 |  |
| Rate difference between NVSI-06-07 and BBIBP-CorV groups (%, 95%CI)^f^ | 19.05 (7.17-30.92) |  |  |  | 20.00 (-15.06-55.06) |  |  |
| Post-booster antibody GMT fold rise (95%CI) | 74.25 (48.25-114.26) | 20.44 (11.33-36.88) | 0.0006 |  | 297.79 (27.19-3261.74) | 62.07 (3.67-1050.49) | 0.2772 |

^a^GMT represent geometric mean titer

^b^The ratio of adjusted GMT between two groups was calculated by “NVSI-06-07/ BBIBP-CorV”, and the non-inferiority threshold of ratio between groups was set to 0.67

^c^Covariance analysis with least square method was used to calculate the adjusted GMT and P value

^d^The ratio of adjusted GMT between two age subgroups was calculated by “<45 years old / ≥45 years old”

^e^Seroconversion was defined as more than 4-fold rise form baseline in neutralizing antibody titer

^f^Rate difference=(NVSI-06-07)-(BBIBP-CorV). Rate difference and 95%CI were estimated by CMH method considering stratification factors

^g^Due to only one participant in the subgroup, P value was not calculated

**Table S11:** Serious adverse event list (SS)

| Description of adverse events | System Organ Class (SOC) | Preferred Term (PT) | Date of vaccination | Start date | End date | Duration days | Days to onset from boost | SAE classification | Outcome | Causality assessment |
| --- | --- | --- | --- | --- | --- | --- | --- | --- | --- | --- |
| Subject number: 0184, Group: NVSI-06-07, Age (years): 24.5, Gender: Female, Nationality: Indian | | | | | | | | | | |
| Acute Bronchitis | Infections and infestations | Bronchitis | 2021-09-22 | 2021-11-14 | 2021-11-16 | 3 | 53 | Hospitalization | Symptoms disappeared | Impossible |
| Subject number: 0255, Group: BBIBP-CorV, Age (years): 37.7, Gender: Female, Nationality: Palestine | | | | | | | | | | |
| Schizoaffective disorder, bipolar type | Psychiatric disorders | Schizoaffective disorder bipolar type | 2021-09-28 | 2021-11-02 | 2021-11-03 | 2 | 35 | Hospitalization | Symptoms disappeared | Impossible |
| Schizoaffective disorder, bipolar type | Psychiatric disorders | Insomnia | 2021-09-28 | 2021-11-02 | 2021-11-03 | 2 | 35 | Hospitalization | Symptoms disappeared | Impossible |
| Schizoaffective disorder, bipolar type | Psychiatric disorders | Schizoaffective disorder bipolar type | 2021-09-28 | 2021-11-09 | 2021-11-11 | 3 | 42 | Hospitalization | Symptoms disappeared | Impossible |
| Schizoaffective disorder, bipolar type | Psychiatric disorders | Aggression | 2021-09-28 | 2021-11-09 | 2021-11-11 | 3 | 42 | Hospitalization | Symptoms disappeared | Impossible |
| Subject number: 2690, Group: NVSI-06-07, Age (years): 41.4, Gender: Male, Nationality: Pakistan | | | | | | | | | | |
| Acute duodenal ulcer with perforation | Gastrointestinal disorders | Duodenal ulcer perforation | 2021-09-16 | 2021-11-07 | 2021-11-10 | 4 | 52 | Hospitalization | Symptoms disappeared | Impossible |
| Acute duodenal ulcer with perforation | Gastrointestinal disorders | Gastrooesophagel reflux disease | 2021-09-16 | 2021-11-07 | 2021-11-10 | 4 | 52 | Hospitalization | Symptoms disappeared | Impossible |
| Subject number: 2691, Group: BBIBP-CorV, Age (years):49.6, Gender: Female, Nationality: Indian | | | | | | | | | | |
| Community acquired pneumonia (CAP) | Infections and infestations | Pneumonia | 2021-09-16 | 2021-09-30 | 2021-10-08 | 9 | 14 | Hospitalization | Symptoms disappeared | Impossible |
| Community acquired pneumonia (CAP) | Immune system disorders | Drug hypersensitivity | 2021-09-16 | 2021-09-30 | 2021-10-08 | 9 | 14 | Hospitalization | Symptoms disappeared | Impossible |
| Klebsiella Cystitis | Infections and infestations | Cystitis klebsiella | 2021-09-16 | 2021-10-11 | 2021-10-19 | 9 | 25 | Hospitalization | Symptoms disappeared | Impossible |
| Klebsiella Cystitis | Gastrointestinal disorders | Duodenitis | 2021-09-16 | 2021-10-11 | 2021-10-19 | 9 | 25 | Hospitalization | Symptoms disappeared | Impossible |
| Klebsiella Cystitis | Gastrointestinal disorders | Chronic gastritis | 2021-09-16 | 2021-10-11 | 2021-10-19 | 9 | 25 | Hospitalization | Symptoms disappeared | Impossible |

**Table S12:** Adverse reactions of booster vaccinations

|  | NVSI-06-07（N=899） | BBIBP-CorV（N=901） | Total （N=1800） | P value^a^ |
| --- | --- | --- | --- | --- |
| **Solicited adverse reactions within 0-7 days** | 166 (18.46)^b^ | 151 (16.76) | 317 (17.61) | 0.3537 |
| Grade 1 | 145 (16.13) | 135 (14.98) | 280 (15.56) | 0.5159 |
| Grade 2 | 37 (4.12) | 29 (3.22) | 66 (3.67) | 0.3190 |
| Grade 3 | 1 (0.11) | 3 (0.33) | 4 (0.22) | 0.6246 |
| **Injection site adverse reactions** | 73 (8.12) | 49 (5.44) | 122 (6.78) | 0.0245^c^ |
| Grade 1 | 64 (7.12) | 41 (4.55) | 105 (5.83) | 0.0208 |
| Grade 2 | 10 (1.11) | 8 (0.89) | 18 (1.00) | 0.6456 |
| Pain | 70 (7.79) | 47 (5.22) | 117 (6.50) | 0.0280 |
| Grade 1 | 61 (6.79) | 39 (4.33) | 100 (5.56) | 0.0237 |
| Grade 2 | 9 (1.00) | 8 (0.89) | 17 (0.94) | 0.8135 |
| Induration | 1 (0.11) | 0 | 1 (0.06) | 0.4994 |
| Grade 1 | 1 (0.11) | 0 | 1 (0.06) | 0.4994 |
| Swelling | 2 (0.22) | 1 (0.11) | 3 (0.17) | 0.6244 |
| Grade 1 | 2 (0.22) | 1 (0.11) | 3 (0.17) | 0.6244 |
| Rash | 1 (0.11) | 2 (0.22) | 3 (0.17) | 1.0000 |
| Grade 1 | 1 (0.11) | 2 (0.22) | 3 (0.17) | 1.0000 |
| Redness | 1 (0.11) | 2 (0.22) | 3 (0.17) | 1.0000 |
| Grade 1 | 1 (0.11) | 2 (0.22) | 3 (0.17) | 1.0000 |
| Pruritus | 6 (0.67) | 2 (0.22) | 8 (0.44) | 0.1784 |
| Grade 1 | 5 (0.56) | 2 (0.22) | 7 (0.39) | 0.2877 |
| Grade 2 | 1 (0.11) | 0 | 1 (0.06) | 0.4994 |
| **Systemic adverse reactions** | 120 (13.35) | 122 (13.54) | 242 (13.44) | 0.9449 |
| Grade 1 | 101 (11.23) | 106 (11.76) | 207 (11.50) | 0.7677 |
| Grade 2 | 29 (3.23) | 24 (2.66) | 53 (2.94) | 0.4898 |
| Grade 3 | 1 (0.11) | 3 (0.33) | 4 (0.22) | 0.6246 |
| Fever | 18 (2.00) | 21 (2.33) | 39 (2.17) | 0.7466 |
| Grade 1 | 15 (1.67) | 18 (2.00) | 33 (1.83) | 0.7259 |
| Grade 2 | 2 (0.22) | 1 (0.11) | 3 (0.17) | 0.6244 |
| Grade 3 | 1 (0.11) | 3 (0.33) | 4 (0.22) | 0.6246 |
| Diarrhea | 2 (0.22) | 4 (0.44) | 6 (0.33) | 0.6870 |
| Grade 1 | 2 (0.22) | 4 (0.44) | 6 (0.33) | 0.6870 |
| Constipation | 4 (0.44) | 1 (0.11) | 5 (0.28) | 0.2177 |
| Grade 1 | 4 (0.44) | 1 (0.11) | 5 (0.28) | 0.2177 |
| Anorexia | 0 | 1 (0.11) | 1 (0.06) | 1.0000 |
| Grade 1 | 0 | 1 (0.11) | 1 (0.06) | 1.0000 |
| Vomiting | 0 | 1 (0.11) | 1 (0.06) | 1.0000 |
| Grade 1 | 0 | 1 (0.11) | 1 (0.06) | 1.0000 |
| Nausea | 3 (0.33) | 3 (0.33) | 6 (0.33) | 1.0000 |
| Grade 1 | 3 (0.33) | 3 (0.33) | 6 (0.33) | 1.0000 |
| Muscle pain (non-inoculation site) | 45 (5.01) | 41 (4.55) | 86 (4.78) | 0.6603 |
| Grade 1 | 37 (4.12) | 34 (3.77) | 71 (3.94) | 0.7183 |
| Grade 2 | 8 (0.89) | 7 (0.78) | 15 (0.83) | 0.8026 |
| Joint pain | 3 (0.33) | 3 (0.33) | 6 (0.33) | 1.0000 |
| Grade 1 | 3 (0.33) | 3 (0.33) | 6 (0.33) | 1.0000 |
| Headache | 48 (5.34) | 56 (6.22) | 104 (5.78) | 0.4797 |
| Grade 1 | 34 (3.78) | 43 (4.77) | 77 (4.28) | 0.3515 |
| Grade 2 | 15 (1.67) | 13 (1.44) | 28 (1.56) | 0.7086 |
| Cough | 10 (1.11) | 5 (0.55) | 15 (0.83) | 0.2078 |
| Grade 1 | 8 (0.89) | 4 (0.44) | 12 (0.67) | 0.2648 |
| Grade 2 | 2 (0.22) | 2 (0.22) | 4 (0.22) | 1.0000 |
| Breathing trouble | 3 (0.33) | 0 | 3 (0.17) | 0.1244 |
| Grade 1 | 3 (0.33) | 0 | 3 (0.17) | 0.1244 |
| Pruritus at non-inoculated sites (no skin damage) | 4 (0.44) | 5 (0.55) | 9 (0.50) | 1.0000 |
| Grade 1 | 3 (0.33) | 4 (0.44) | 7 (0.39) | 1.0000 |
| Grade 2 | 1 (0.11) | 1 (0.11) | 2 (0.11) | 1.0000 |
| Acute allergic reaction | 3 (0.33) | 1 (0.11) | 4 (0.22) | 0.3740 |
| Grade 1 | 2 (0.22) | 1 (0.11) | 3 (0.17) | 0.6244 |
| Grade 2 | 1 (0.11) | 0 | 1 (0.06) | 0.4994 |
| Fatigue | 27 (3.00) | 38 (4.22) | 65 (3.61) | 0.2062 |
| Grade 1 | 19 (2.11) | 35 (3.88) | 54 (3.00) | 0.0373 |
| Grade 2 | 8 (0.89) | 3 (0.33) | 11 (0.61) | 0.1444 |
| **Unsolicited adverse reactions within 0-30 days** | 67 (7.45) | 66 (7.33) | 133 (7.39) | 0.9285 |
| Grade 1 | 50 (5.56) | 54 (5.99) | 104 (5.78) | 0.7620 |
| Grade 2 | 25 (2.78) | 15 (1.66) | 40 (2.22) | 0.1129 |
| **Overall adverse reactions within 0-30 days** | 184 (20.47) | 177 (19.64) | 361 (20.06) | 0.6805 |
| Grade 1 | 162 (18.02) | 160 (17.76) | 322 (17.89) | 0.9022 |
| Grade 2 | 54 (6.01) | 40 (4.44) | 94 (5.22) | 0.1394 |
| Grade 3 | 1 (0.11) | 3 (0.33) | 4 (0.22) | 0.6246 |

^a^*P* value is calculated using Fisher's exact test

^b^Data presents as n (%)

^c^Light blue color denotes there is statistically significant difference in the adverse reaction between NVSI-06-07 and BBIBP-CorV boosting groups
