# Supplementary material for: Immunogenicity and safety of NVSI-06-07 as a heterologous booster after priming with BBIBP-CorV: a phase 2 trial
Source: Signal Transduct Target Ther. 2022 Jun 6;7:172. doi: 10.1038/s41392-022-00984-2 (PMC9167817; doi:10.1038/s41392-022-00984-2)
Supplement: Supplementary file 2 — Supplementary Protocol [file 41392_2022_984_MOESM2_ESM.pdf]

Clinical Trial Protocol

Sequential Immunization of Recombinant COVID-19 vaccine (CHO cells) and Inactivated COVID-19 Vaccine (Vero cells) in Population Aged 18 Years and Above

Protocol Title:	Clinical study on Sequential Immunization of Recombinant COVID-19 Vaccine (CHO cells) and Inactivated COVID-19 Vaccine (Vero cells) in Population Aged 18 Years and Above
Product Name:	Recombinant COVID-19 Vaccine (CHO cells)
Specification:	20ug/dose/0.5 ml
Protocol Number:	CNBG-REC-2021002
Version Date:	23 July, 2021
Version Number:	1.0

Sponsor (Seal): China National Biotec Group Co., Ltd (CNBG)

Sinopharm CNBG National Vaccine & Serum Institute

Lanzhou Institute of Biological Products Co., Ltd

Responsible Institute (seal):

Statistical Institute (Seal):

TABLE OF CONTENTS

TABLE OF CONTENTS 3
STATEMENT OF COMPLIANCE 6
INVESTIGATOR'S COMPLIANCE DECLARATION 7
List of abbreviations 8
Synopsis 10
1. Introduction 17
2. Background and Principle 17
2.1 Background of disease 17
2.2. Pathogenic background 18
2.3. Vaccine background 19
3. Study Objective 22
3.1. Primary Objectives 22
3.2. Secondary Objectives 22
3.3 Exploratory Objectives 23
4. Study Design 23
4.1. Trial Description 23
4.2. Procedures and Methodology 23
4.3. Study Endpoint 25
4.4 Trial Hypothesis 25
4.5 Safety Observation Indicators and Grading Criteria 26
4.6 Randomization and Blinding 31
5. Screening and Withdrawal of Subjects 32
5.1 Subject Population 32
6. Product Introduction 34
6.1 Description and Characteristics of the Investigational Products 34
6.2 Vaccine Packaging and Labeling 35
6.3 Vaccine Storage and Transportation 36
6.4 Administration and Immunization Schedule 37
7. Study Workflow and Visiting Method 38
7.1 Schedule of Visits 38
7.2 Study Procedure 38
7.3 Safety Observation and Follow-up 40

7.4 Laboratory Testing 41
7.5 Concomitant Medication 45
7.6 Criteria for Suspension or Early Termination 45
7.7 Protocol Violation and Deviation 46
7.7 Study Duration 46
7.8 Site Closure 46
8. Safety Definition, Reporting and Monitoring 47
8.1 General Precautions 47
8.2 Risk Prevention Measures Related to COVID-19 47
8.3 Handling and Reporting of Serious Adverse Events 48
8.4. Outcome of Serious Adverse Events 51
9. Completion of Clinical Trial 51
10. Statistical Considerations 51
10.1 Sample Size Considerations 51
10.2 Analysis Set 51
10.3 Statistical Method 52
10.4 Subgroup Analysis 53
10.5 Interim Analysis 53
10.6 Multiplicity 53
10.7 Handling of Missing Data 53
11. Ethical and Regulatory Matters 54
11.1. Ethics Committees and Regulatory Authorities 54
11.2. Responsibilities of the Sponsor 55
11.3. Responsibilities of the Investigator 55
11.4. Subject Information and Consent 56
11.5. Compensation to Subjects 57
11.6. Subject Confidentiality 57
11.7. Amendment to Subject Related Information 57
11.8. Direct Access to Source Documentation 58
12. Study Management 59
12.1. Case Report Form (eCRF) Handling 59
12.2. Source Data and Subject Files 59

12.3. Investigator Site File and Archiving ... 60
12.4. Monitoring, Quality Assurance and Inspection by Authorities ... 61
12.5. Changes to the Study Protocol ... 61
12.6. Study Report and Publication Policy ... 62
13. References ... 63

STATEMENT OF COMPLIANCE

This study will be performed in compliance with Good Clinical Practice (GCP), the Declaration of Helsinki (with amendments) and local legal and regulatory requirement.

Signature Page

The signature below constitutes the approval of this protocol and the attachments and provides the necessary assurances that this trial will be conducted according to all stipulations of the protocol, including all statements regarding confidentiality, and according to Good Clinical Practice (GCP), Declaration of Helsinki (with amendments) and the laws and regulations of the countries in which the study takes place.

<table>
<tr><td>Protocol name</td><td colspan="2">Clinical study on Sequential Immunization of Recombinant COVID-19 Vaccine (CHO cells) and Inactivated COVID-19 Vaccine (Vero cells) in Population Aged 18 years and above</td></tr>
<tr><td>Protocol number</td><td colspan="2">CNBG-REC-2021002</td></tr>
<tr><td>Version date</td><td colspan="2">Jul 23, 2021</td></tr>
<tr><td>Version number</td><td colspan="2">Version 1.0</td></tr>
<tr><td>Sponsor</td><td colspan="2">China National Biotec Group Co. Ltd. (CNBG)
Sinopharm CNBG National Vaccine & Serum Institute
Lanzhou Institute of Biological Products Co., Ltd</td></tr>
<tr><td>Sponsor Project leader</td><td>Name: Yang Yunkai
Company: China National Biotec Group Co., Ltd. (CNBG)
Address: No.B 2 Shuangqiao Road, Chaoyang District, Beijing, 100024 P.R. of China
Zip Code: 100024
Tel: 13601126881
E-mail: yangyunkai@sinopharm.com</td><td>Signature:

Date:</td></tr>
</table>

INVESTIGATOR'S COMPLIANCE DECLARATION

- I have read this protocol and agree to conduct the study as outlined herein, and as implemented by any future protocol amendment/update, according to the terms of the clinical trial contract, and in accordance with supplementary study: conduct procedures and/or guidance or documents of this study, complying with the obligations and requirements of clinical investigators and all other requirements listed in relevant national and international regulations including ICH GCP guidelines.8
- I assume responsibility for the compliance of the site personnel reporting to me or assisting me with the study.
- I confirm that I am aware of my obligations towards relevant regulatory authorities, as it concerns my participation in this study as a researcher.
- I agree to disclose and provide information to the Sponsor on any potential conflict of interest I may have participating in this study as investigator.
- I declare that I will co-operate with the Sponsor personnel and/or representatives, and vendors managing or supporting the study, including CRO, timely and adequately to ensure timely study conduct and compliance with study documents and relevant regulations.
- I am fully familiar with correct method of using vaccine described in the protocol, and other information provided by sponsor, including but not limited to the following contents: current investigator's brochure (IB) or equivalent documents and relavant supplements.
- I am familiar with and will abide by the GCP, the Guiding Principles for Quality Management of Vaccine Clinical Trials (Tentative) and all existing regulatory requirements.

**Protocol Name**	Clinical trial on Sequential Immunization of Recombinant COVID-19 vaccine (CHO cells) and Inactivated COVID-19 Vaccine (Vero cells) in Population aged 18 years and above
**Protocol Number**	CNBG-REC-2021002
**Version date**	Jul 23, 2021
**Version Number**	Version 1.0

Name of Investigator (please print) ______________________

Investigator Signature: ________________________________

Date: ____

CONFIDENTIAL

LIST OF ABBREVIATIONS

ACE2	Angiotensin converting enzyme 2
ADE	Antibody Dependence Enhancement
AE	Adverse Event
BP	Blood Pressure
CI	Confidence Interval
COA	Certificate of Analysis
CoV	Coronavirus
COVID-19	Coronavirus disease of 2019
DNA	Deoxyribonucleic acid
DSMB	Data and Safety Monitoring Board
ECRF	Electronic Case Report Form
EDC	Electronic Data Capture System
EAC	Endpoint Information Committee
FAS	Full Analysis Set
GCP	Good Clinical Practice
GMP	Good Manufacturing Practice
GMT	Geometric Mean Titer
GMI	Geometric mean increase
IB	Investigator's Brochure
ICF	Informed Content Form
IEC	Independent Ethics Committee
MERS	Middle East Response Syndrome
NMPA	National Medical Products Administration
MFAS	Modified Full Analysis Set
PPS	Per-Protocol Set
PCR	Polymerase Chain Reaction
RNA	Ribonucleic acid
SAE	Serious Adverse Events
SARS	Severe Acute Respiratory Syndrome
SOP	Standard Operating Procedure
SS	Safety Set
SUSAR	Suspected and Unexpected Serious Adverse Reactions

CONFIDENTIAL

VED	Vaccine Enhanced Disease
WHO	World Health Organization

CONFIDENTIAL

Synopsis

**Study Title**	Clinical trial on Sequential Immunization of Recombinant COVID-19 Vaccine (CHO cells) and Inactivated COVID-19 Vaccine (Vero cells) in Population aged 18 years and above
**Products Characteristic**	This recombinant protein was expressed by recombinant Chinese hamster ovary (CHO) cells and designed based on the receptor-binding domain (RBD) of SARS-CoV-2 Spike (S) protein. Engineered cells were cultured, harvested and purified by a series of processes, and then added with aluminum hydroxide adjuvant.
**Indication**	This product is used for population aged 18 years and above. Vaccination with this vaccine can stimulate the body to produce immune response to SARS-CoV-2, which is used to prevent COVID-19.
**Study Design**	This is randomized, blinded and positive controlled design. Among the subjects who have been vaccinated with two doses of Inactivated COVID-19 vaccine (Vero cell), based on a step-wise approach, the subjects will receive one dose of recombinant COVID-19 vaccine sequentially at different shedules of 1-3 months, 4-6 months and ≥ 6 months after two doses of vaccination, and the subjects vaccinated at different schedules will be randomly assigned to different sequential immunization groups. At the same time, each sequential immunization group will be matched with a control group with the inactivated COVID-19 vaccine (vero cells) as the booster dose.<br>**Sample Size**: Total sample size is 1,800, which will be randomly assigned to recombinant vaccine sequential group and inactivated vaccine sequential group in a 1: 1 ratio, with 300 subjects in each sequential group.<br>**Immunization Schedule:**<br>The subjects who have been vaccinatied with two doses of inactivated COVID-19 vaccine according to the immunization schedule of 0 and 21 (+7) days will receive sequential vaccination of one dose of recombinant COVID-19 vaccine (CHO cells) or inactivated COVID-19 Vaccine (Vero cells) at different schedules of 1-3 months, 4-6 months and ≥6 months.<br>**Route of Administration**: lateral deltoid muscle of upper arm, intramuscular injection<br>**Safety Observation:**

The subjects will be observed at the study site for 30 minutes after vaccination, and local and systemic adverse events will be collected. Within 0-30 days after sequential vaccination of booster dose, the local and systemic reactions of the subjects will be actively followed up and recorded on the diary card.

Serious adverse events (SAEs) will be collected from the first dose of vaccination and up to 6 months following the full course of immunization.

Immunogenicity Observation:

Blood samples of all the subjects will be collected before the vaccination of sequential dose and 14 days, 28 days, 3 months, 6 months, 9 months and 12 months after sequential vaccination for detection of neutralizing antibodies and anti-protein specific IgG antibodies.

Collect 12.5ml blood samples of all the subjects and separate serum. Nentralizing antibody will be determined by microdose cytopathogenic efficiency (CPE) assay and specific IgG antibody will be tested by enzyme-linked immunosorbent assay (ELISA).

Time points of blood sample collection is described in Table 1 Sample Size and Procedures of Clinical Trial.

Table 1 Sample Size and Procedures of Clinical Trial

Sequential Vaccination Time of Recombinant Vaccine	Booster Immunization	Sequential Vaccine Group	Sample Size	Safety	Blood Collection Time Point
1-3 months after 2 doses	31-90 days (+14)	Recombinant Vaccine	300	Collect AEs within 0-7 days and 8-30 days after immunization with sequential booster dose, and SAEs up to 6 months following immunization with the booster dose.	Before the sequential dose ①14 days②, 28days③, 3 months④, 6 months⑤, 9 months⑥, 12 months⑦after sequential
		Inactivated Vaccine	300		
4-6 months after 2 doses	91-180 days (+14)	Recombinant Vaccine	300		
		Inactivated Vaccine	300		
≥ 6 months after 2 doses	≥ 181 days	Recombinant Vaccine	300		
		Inactivated vaccine	300		

					vaccination of one dose.
Total sample size			**1800**		

Note:

1) Blood collection window at 14 days, 28 days is +10 days, blood collection window at 3M, 6M, 9M, 12M is +30 days.

2) If the blood sample test results show that the antibody level is too low after the full immunization, blood collection at subsequent time points will not be carried out.

Study Objective

Primary Objectives:

To evaluate the immunogenicity of recombinant COVID-19 vaccine (CHO cells) and inactivated COVID-19 vaccine (Vero cells) 14 days after sequential vaccination of one dose in populations≥18 years of age.

To evaluate the immunogenicity of recombinant COVID-19 vaccine (CHO cells) and inactivated COVID-19 vaccine (Vero cells) 28 days after sequential vaccination of one dose in populations≥18 years of age.

Secondary Objectives:

To evaluate the safety of recombinant COVID-19 vaccine (CHO cells) and inactivated COVID-19 vaccine (Vero cells) 28 days after sequential vaccination of one dose in populations ≥18 years of age.

To evaluate the optimal immunization schedule of sequential vaccination of recombinant COVID-19 vaccine (CHO cells) in populations≥18 years of age.

To evaluate the immune persistence of sequential vaccination of recombinant COVID-19 vaccine (CHO cells) and inactivated COVID-19 vaccine (Vero cells) in populations≥18 years of age.

Exploratory Objectives:

To evaluate the vaccine efficacy of recombinant COVID-19 vaccine (CHO cells) against COVID-19, especially severe cases, and deaths 14 days after sequential vaccination at different schedules.

	To evaluate the vaccine efficacy of recombinant COVID-19 vaccine (CHO cells) against different variants 14 days after sequential vaccination at different schedules.
**Investigational Vaccine**	Investigational vaccine: Recombinant COVID-19 Vaccine (CHO cell)<br>Manufacturer: Sinopharm CNBG National Vaccine & Serum Institute<br>Lanzhou Institute of Biological Products Co., Ltd<br>Specifications: 0.5 ml/vial. The dose for human use is 0.5 mL per time, which contains 20ug of viral protein antigen.<br>Storage conditions: 2-8 ℃<br>Batch number: 20201113 Expiry date: Nov 11, 2022<br><br>Control vaccine: Inactivated SARS-CoV-2 Vaccine (Vero cell)<br>Manufacturer: Beijing Institute of Biological Products Co., Ltd.<br>Specifications: 0.5 ml/vial. The dose for human use is 0.5 mL per time, which contains 6.5U of antigen.<br>Storage conditions: 2-8 ℃<br>Batch number: 2021071710 Expiry date: Jul 8, 2023
**Inclusion Criteria**	- Age range: populations aged 18 years and above.<br>- Judged by the investigator that the health condition is well after inquiry and physical examination.<br>- Vaccinated with 2 doses of Sinopharm-CNBG inactivated COVID-19 vaccine according to the product insert.<br>- Female subjects who are not nursing or pregnant at the time of enrolment (negative urine pregnancy test) and have no family planning within the first 6 months after enrollment. Effective contraceptive measures have been taken within 2 weeks before inclusion.<br>- During the whole follow-up period of the study, be able and willing to complete the whole prescribed study plan;<br>- With self ability to understand the study procedures, the informed consent & voluntarily sign an informed consent form and is able to comply with the requirements of the clinical study protocol.

**Excluion Criteria**	- Confirmed cases, suspected cases or asymptomatic cases of COVID-19;<br>- With a history of SARS and MERS infection (self-report, on-site inquiry);<br>- Has vaccinated with any other vaccine rather than one or more doses of Sinopharm-CNBG inactivated COVID-19 vaccine;<br>- Axillary temperature ≥ 37.3 ℃ (forehead temperature ≥ 37.8 ℃);<br>- Previous severe allergic reactions to vaccination (such as acute allergic reactions, urticaria, dyspnea, angioneurotic edema or abdominal pain)<br>- Allergy to known components of recombinant COVID-19 vaccine (e.g., aluminum and histidine);<br>- Severe respiratory diseases, severe liver and kidney diseases, hypertension (systolic blood pressure ≥ 150 mmHg, diastolic blood pressure ≥ 90 mmHg), diabetic complications, malignant tumors, various acute diseases or acute attacks of chronic diseases;<br>- Has been diagnosed with congenital or acquired immunodeficiency, HIV infection, lymphoma, leukemia or other autoimmune diseases;<br>- Has a history of convulsions, epilepsy, encephalopathy, long-term alcohol and drug abuse, history of thyroidectomy, history of infectious diseases, mental illness or family history (direct);<br>- With congenital malformation or developmental disorder, genetic defect, severe malnutrition, etc.;<br>- Has a history of coagulation dysfunction (e.g., coagulation factors deficiency and coagulation diseases);<br>- Asplenia or splenectomy, functional asplenia caused by any situation;<br>- Receving anti-TB (tuberculosis) therapy;<br>- Received immunoenhancement or inhibitor therapy within 3 months (continuous oral or IV administration for more than 14 days);<br>- Received other vaccines within 14 days before vaccination;<br>- Received blood products within 3 months before vaccination;<br>- Received other investigational drugs within 6 months before vaccination;<br>- Plans to move before the end of the study or leave the local area for a long time during the scheduled study visits;

	- Other circumstances judged by investigators that are not suitable for this clinical trial**.**
**Subject's early withdrawal**	**Early withdrawal means that the subject fails to complete the vaccination and blood collection procedures in accordance with clinical study protocol, and investigator decides whether to continue the follow-up related research according to the situation. When any of the following conditions occur, the subject will withdraw from the clinical trial in advance.**<br>- The subject requests to withdraw from the clinical trial;<br>- Intolerable adverse events, whether related to the investigational drug or not;<br>- The health status of the subjects does not allow them to continue to participate in this trial;<br>- The subjects are vaccinated with other clinical investigational vaccines during the study period;<br>- Any other reason that investigator considers.
**Criteria for suspension and early termination**	**In case of any of the following circumstances, the trial shall be suspended, and the investigator, the sponsor and the Ethics Committee shall jointly hold a meeting to decide whether to terminate the clinical trial early:**<br>- The number of subjects with adverse reactions of severity Grade 3 or above exceeds 15% of the subjects vaccinated in this subgroup;<br>- One case of Grade 4 adverse reaction related to vaccination or SUSAR has occured.<br>**In case of any of the following circumstances, the clinical trial shall be terminated early:**<br>- Sponsor has found that the vaccine has potential safety hazards or the trial has quality related problems, and requiring the complete termination of the study.<br>- The Ethics Committee requests the termination of the trial because of the violation of ethics in the trial.<br>- Request by the administrative authority to terminate the trial.
**Trial End point**	**Immunogenicity endpoint:**<br>**Primary endpoint:**<br>GMT and the 4-fold rise of anti-SARS-CoV-2 neutralizing antibody 14 days after sequential vaccination of one booster dose;<br>GMT and 4-fold rise of anti- SARS-CoV-2 neutralizing antibody 28 days after sequential

	vaccination of one booster dose.<br>**Secondary endpoint:**<br>GMT of anti-SARS-CoV-2 IgG antibody, 4-fold rise, GMI, and proportions of neutralizing antibody titered ≥ 1: 16, ≥ 1: 32 and ≥ 1: 64 before vaccination and 28 days after vaccination of the sequential booster dose.<br>**Immune persistence endpoint:**<br>Anti-SARS-CoV-2 neutralizing antibody and GMT of IgG antibody 3 months, 6 months, 9 months and12 months after full course of immunization, and the proportions of neutralizing antibody titered ≥ 1: 16, ≥ 1: 32 and ≥ 1: 64.<br><br>**Safety endpoint:**<br>The incidence and serverity of any adverse reactions/events within 30 minutes after vaccination;<br>The incidence and serverity of solicited adverse reactions/events within 0-7 days after vaccination;<br>The incidence and severity of unsolicited adverse reactions/events within 8-30 days after vaccination;<br>The incidence of SAE and AESI observed after vaccination and up to 6 months after full course of immunization.<br>**Exploratory entpoints:**<br>The vaccine efficay of recombinant COVID-19 vaccine (CHO cells) against COVID-19, severe cases and deaths after 14 days following sequential vaccination in population aged 18 years and above.

Clinical Trial Protocol

Sequential Immunization of Recombinant COVID-19 vaccine (CHO cells) and Inactivated COVID-19 vaccine (Vero cells) in Population Aged 18 years and above

CONFIDENTIAL

1. INTRODUCTION

The recombinant COVID-19 vaccine (CHO cell) developed by Sinopharm CNBG National Vaccine & Serum Institute (NVSI) and Lanzhou Institute of Biological Products Co., Ltd. (LIBP) affiliated to China National Biotec Group Co., Ltd (CNBG) is used to prevent the diseases caused by SARS-CoV-2. After being reviewed by National Medical Products Administration (NMPA) in accordance with *Drug Administration Law of the People's Republic of China*, *Vaccine Administration Law of the People's Republic of China* and *Provisions for Drug Registration*, it has been approved for Phase I/II clinical study in China, and the data obtained from the Phase I/II clinical trial in population aged 18 years and above completed showed good safety profile.

In this project, a randomized, blinded, positive control method will be used to evaluate the safety and immunogenicity of sequential vaccination of recombinant COVID-19 vaccine (CHO cells) in healthy population aged 18 years and above. This study was approved by the Ministry of Health of UAE.

2. BACKGROUND AND PRINCIPLE

2.1 Background of disease

According to WHO data, as of July 23, 2021, there were 194,595,271 confirmed cases and 4,165,733 deaths due to COVID-19, with a mortality rate of 2.1%. The infection sources of COVID-19 are mainly SARS-CoV-2 infected patients and asymptomatic infected persons, but it is found that patients in incubation period are also contagious, and they are highly contagious within 5 days after onset. SARS-CoV-2 is mainly transmitted through respiratory droplets and close contact, which may be transmitted by aerosol. Contact with virus contaminated articles can also cause infection. Because SARS-CoV-2 can be isolated from patients' feces and urine, environmental pollution caused by feces and urine can also cause aerosol or contact transmission.

People are generally susceptible, and they can get infection in all ages. People can get certain immunity after infection or vaccination with COVID-19 vaccine, but the duration is not clear. Based on the current epidemiological investigation, the incubation period of virus is generally 1 ~ 14 days, mostly 3 ~ 7 days, and the incubation period of very few cases can reach 24 days.

The main clinical symtoms of COVID-19 are fever, dry cough and fatigue, and the first symptoms of some patients are loss of smell and taste. A few patients are accompanied by nasal congestion, runny nose, sore throat, muscle pain and diarrhea. According to clinical symptoms, it can be divided into four categories: mild, moderate, severe and critical. Severe patients often have dyspnea and/or hypoxemia one week after onset, and severe patients can rapidly progress to acute respiratory distress syndrome, septic shock, metabolic acidosis, coagulation dysfunction and multiple organ failure. Very few patients may also have central nervous system involvement and acrovascular necrosis. It is worth noting that severe and critical patients may have moderate to low fever or even no obvious fever during the course of disease. Mild patients may show low fever, slight fatigue, smell and taste disorders, etc., without pneumonia. A few patients have no obvious clinical symptoms after being infected with SARS-CoV-2. According to the current cases, most patients have a good prognosis, while a few patients are in critical condition. The prognosis of the elderly and those with chronic basic diseases is poor. The clinical process of pregnant women with COVID-19 is similar to that of patients of the same age. Children's cases have relatively mild symptoms.

2.2. Pathogenic background

SARS-CoV-2 belongs to β coronavirus. Under cryoelectron microscope, it is approximately spherical particles with variable diameter, mainly around 100nm. SARS-CovV-2 contains dense virus matrix, surrounded by lipid bilayer, and its outer surface protrudes. It is an enveloped virus, which is composed of non-segmental positive-sense single-stranded RNA and protein. It belongs to Orthocoronavirinae and is widely distributed in humans and other mammals.

The genome of SARS-CoV-2 strain is 29.9 kb long and encodes 9860 amino acids. SARS-CoV-2 has four main structural proteins, known as spike protein (S), membrane protein (M), envelope protein (E) and nucleocapsid protein (N), as well as several helper proteins. S, M and E proteins constitute the viral envelope and are the main surface antigens of virus-induced immune response. S protein is a transmembrane glycoprotein with a molecular weight of about 150kDa, which is divided into two subunits, namely S1 and S2, by Furin protease of host cells. S1 contains one RBD, which is responsible for determining the cell targeting between host cells and virus receptor binding domains; As a membrane fusion subunit, S2 can mediate virus to fuse with host

CONFIDENTIAL

cells. M protein plays an important role in determining the shape of viral envelope. It can bind with other structural proteins, and binding with N protein helps to stabilize nucleocapsid and promote the assembly of virus. E protein plays an important role in the process of virus production and maturation. N protein is located in the core of virus and combines with viral RNA to form nucleocapsid. N protein has a highly conserved sequence, and it participates in virus replication and infection of host cells.

The binding of spike protein (S protein) with angiotensin converting enzyme 2 (ACE2) protein is essential for SARS-CoV-2 infecting human cells. After fusion, transmembrane protein serine 2 (TMPRSS2), which exists on the surface of host cells, scavenges ACE2 and activates S protein. Activation of the S protein causes a conformational change and allows the virus to enter the host cell. The affinity of S protein of SARS-CoV-2 with ACE2 is 10 to 20 times that of SARS virus, which may be the reason of strong transmission of COVID-19. ACE2 expressed in respiratory epithelial cells and type II alveolar cells of lung is the receptor of SARS-CoV-2 entering host cells. Besides lung, ACE2 is also distributed in intestinal epithelial cells of ileum and colon, vascular smooth muscle cells, proximal tubular epithelial cells of kidney, urinary tract epithelial cells of bladder and testis. Therefore, COVID-19 patients will not only have respiratory diseases, but also have heart, kidney, and digestive system diseases. ACE2 expression in vascular intima can damage endothelial cells and lead to the risk of thrombotic events. In addition, the immune system releases a large number of cytokines to viral infection sites or secondary infection, which can induce cytokine storm and septicemia symptoms.

In addition, coronavirus is sensitive to ultraviolet rays and heat. For 30 minutes at 56 ℃, ether, 75% ethanol, chlorine-containing disinfectant, peracetic acid and chloroform can effectively inactivate the virus, but chlorhexidine cannot effectively inactivate the virus.

2.3. Vaccine background

As of 26 March 2021, according to the statistics from World Health Organization (WHO), there are 267 COVID-19 vaccine projects in active research and development around the world, with diversified technical routes, mainly including inactivated vaccines, recombinant protein vaccines, viral vector vaccines and nucleic acid vaccines. There are 83 candidate vaccines in clinical stage, including 27 recombinant protein vaccines, among which NVX-CoV2373 developed by Novavax is in Phase III stage, recombinant protein vaccine developed by Zhifei Bio has been authorized for emergency use in China, and others are in Phase I/II study. And four inactivated vaccines, four recombinant viral vector vaccines and four nucleic acid vaccines are in

CONFIDENTIAL

Phase III clinical trial.

2.3.1 Inactivated vaccine

Inactivated vaccine is made from virus that has been grown in cell culture and have been lose its pathogenicity by physical or chemical method, but only retain its antigenicity. As a traditional vaccine preparation method, the process is mature. At present, there are 22 inactivated vaccines under research around the world, of which 11 are in clinical stage. The inactivated vaccines of Sinopharm CNBG Wuhan Institute of Biological Products and Beijing Institute of Biological Products have carried out Phase III clinical study in UAE, and have been approved for conditional marketing in 8 countries including UAE and China, and authorized emergency use in 86 countries. The published clinical results showed good safety and efficacy. The seroconversion rate of antibody in two-dose immunization schedule is 100%, and the vaccine efficacy is 79%. Phase III clinical results of inactivated vaccine CoronaVac of Sinovac Research & Development Co., Ltd. in Brazil, Turkey and Indonesia showed that the vaccine efficacy is higher than 50% efficacy threshold set by the World Health Organization, and it has been approved for conditional marketing in China and authorized for emergency use in many countries and regions.

2.3. 2 Recombinant Protein Vaccine

Compared with other types of vaccines, recombinant protein vaccines are one of the key directions of research and development in novel vaccines due to its significant advantages in safety and low cost. At present, 96 COVID-19 recombinant protein vaccine candidates are under development, and 27 of them are at clinical evaluation stage.

The recombinant nanoparticle vaccine NVX-COV2373 developed by NOVAVAX consists of full-length SARS-CoV-2 spike glycoprotein trimer and MATRIX-MTM adjuvant. The latest published phase III clinical interim results from UK showed that among 15,000 volunteers aged 18-84 years (27% volunteers are over 65 years old), the overall efficacy was 89.3% (56 cases in the placebo group and 6 cases in the vaccine group), and the efficacy against B.1.1.7 variant was 85.6%. According to the clinical data from Phase IIB in South Africa, among the 4,400 volunteers (6% were HIV positive), the efficacy of NVX-COV2373 in HIV negative population was 60% (29 cases in placebo group and 15 cases in vaccine group), and the efficacy of 49.4% if HIV positive population was included. Among COVID-19 infections, 90% cases were 501Y.V2 variants.

In the research pipeline of China, Zifei's recombinant protein vaccine (CHO cell) contains of SARS-COV-2 S protein receptor binding domain (RBD) unique dimer as antigen, adjuvanted by traditional adjuvant, and has been authorized for emergence use in China. The published clinical phase II data showed that among 900 volunteers, the seroconversion rate of neutralizing antibody

was 97% and the titer of neutralizing antibody (GMT) was 102.5 (CPE test method), which was twice as high as the antibody in the convalescent serum. In terms of safety, no serious adverse events related to vaccines were observed. In addition, Clover Biopharmaceuticals' recombinant protein vaccine is also in Phase II/III clinical trial.

2.3. 3 Recombinant Viral Vector Vaccines

Viral vector vaccine uses replicative or non-replicative viruses as vectors, recombines specific antigen gene fragments into the vector genome, and delivery them into host cells, and induces expression and induces corresponding immune response. Currently, there are 19 recombinant viral vector vaccines in clinical stage, of which 3 are in the process of Phase III clinical trial.

AZD1222 (CHADOX1 NCOV-19), jointly developed by Oxford University and AstraZeneca, uses replication-deficient chimpanzee adenovirus CHADOX1 as vector to expresses SARS-CoV-2 S protein, and has been authorized for emergency use in UK, Brazil and other countries and regions. Recently, the European Union approved the conditional marketing of the vaccine for immunization in population aged 18 years and above, and the efficacy was evaluated as 59.5%. Previously, the Phase III clinical results published by AstraZeneca from the UK and Brazil trials showed that the average vaccine efficacy was 70% (vaccine efficacy of 90% with 1.5 doses, N=2741; vaccine efficacy of 62% with full doses, N=11636).

AD5-NCOV, jointly developed by Academy of Military Sciences and CanSino, uses replication-defective human adenovirus type 5 as vector to expresses SARS-CoV-2 S protein, and has been approved for conditional marketing in China. The published phase II clinical data showed that 28 days after single dose inoculation, the titers of RBD specific antibody in high dose group (1 × 1011) and low dose group (5 × 1010) were 656.5 and 571.0, respectively. The titer of neutralizing antibody was 19.5 and 18.3 respectively, and the seroconversion rate of neutralizing antibody was 59% and 47% respectively.

2.3. 4 NUCLEIC ACID VACCINE

Nucleic acid vaccines introduce DNA or mRNA encoding specific antigens into human cells, express corresponding antigen proteins, and induce immune response. Currently, there are 60 nucleic acid vaccines in active research and development, and 21 vaccines in clinical stage, including 11 mRNA vaccines and 10 DNA vaccines.

mRNA-1273 developed by MODERNA, uses liposome nanoparticles (LNP) delivery system, and encodes SARS-CoV-2 S-2P antigen (composed of SARS-CoV-2 glycoprotein and complete S1-S2 cleavage site). mRNA-1273 has been granted emergency use authorization by FDA in mid-

CONFIDENTIAL

December 2020. The results of a Phase III clinical trial showed that among more than 30,000 subjects who received vaccine or placebo randomly, the vaccine efficacy was 94.1% (185 cases in the placebo group versus 11 cases in the vaccine group), and there was zero case of severe COVID-19 in the vaccine group.

mRNA vaccines BNT162B1 and BNT162B2 jointly developed by BIONTECH, Pfizer and Fosun Pharma uses LNP delivery system and encode SARS-CoV-2 S protein RBD trimer and full-length S protein respectively. BNT162B2 has been authorized for emergency use in the United States, the European Union and other regions, and is the first COVID-19 vaccine authorized by WHO for emergency use listing. The previously published Phase III clinical trial data showed that among 44,000 subjects, vaccine efficacy after two doses of vaccine was 95% (162 cases in the placebo group and 8 cases in the vaccine group).

3. STUDY OBJECTIVE

To evaluate the safety and immunogenicity of sequential vaccination of recombinant COVID-19 vaccine (CHO cells) developed by Sinopharm CNBG National Vaccine & Serum Institute in healthy population aged 18 years and above.

3.1. Primary Objectives

To evaluate the immunogenicity of recombinant COVID-19 vaccine (CHO cells) and inactivated COVID-19 vaccine (Vero cells) 14 days after sequential vaccination of one dose in population≥18 years of age.

To evaluate the immunogenicity of recombinant COVID-19 vaccine (CHO cells) and inactivated COVID-19 vaccine (Vero cells) 28 days after sequential vaccination of one dose in population≥18 years of age.

3.2. Secondary Objectives

To evaluate the safety of recombinant COVID-19 vaccine (CHO cells) and inactivated COVID-19 vaccine (Vero cells) 28 days after sequential vaccination of one dose in population≥18 years of age.

To evaluate the optimal immunization schedule of sequential vaccination of recombinant COVID-19 vaccine (CHO cells) in population≥18 years of age.

To evaluate the immune persistence of sequential vaccination of recombinant COVID-19 vaccine (CHO cells) and inactivated COVID-19 vaccine (Vero cells) in population≥18 years of

CONFIDENTIAL

age.

3.3 Exploratory Objectives

To evaluate the vaccine efficacy of recombinant COVID-19 vaccine (CHO cells) against COVID-19, especially severe cases, and deaths, after 14 days following sequential vaccination at different schedules.

To evaluate the vaccine efficacy of recombinant COVID-19 vaccine (CHO cells) against different variants after 14 days following sequential vaccination at different schedules.

4. STUDY DESIGN

4.1. Trial Description

This clinical trial is a randomized, blinded and positive controlled design.

4.2. Procedures and Methodology

Sample size:

Total of 1,800 healthy subjects aged 18 years and above who have been vaccinated with 2 doses of inactivated COVID-19 vaccine, will be enrolled, and randomly assigned to experimental group and control group in a 1:1 ratio., with 300 subjects in each sequential group.

Immunization Schedule:

The subjects who have been vaccinatied with two doses of inactivated COVID-19 vaccine according to the immunization schedule of 0 and 21 (+7) days will receive sequential vaccination of one dose of recombinant COVID-19 vaccine (CHO cells) or inactivated COVID-19 Vaccine at different schedules of 1-3 months, 4-6 months and ≥6 months.

Route of Administration: lateral deltoid muscle of upper arm, intramuscular injection

Safety Observation:

The subjects will be observed at the study site for 30 minutes after vaccination, and local and systemic adverse events will be collected. Within 0-30 days after vaccination, the local and systemic reactions of the subjects will be actively followed up and recorded on the vaccination diary card.

Serious adverse events (SAEs) need to be monitored from the first dose of vaccination and up to 6 months following the full course of immunization.

Immunogenicity Observation:

Blood samples of all the subjects will be collected before the vaccination of sequential dose and 14 days, 28 days, 3 months, 6 months, 9 months and 12 months after sequential vaccination for detection of neutralizing antibodies and anti-protein specific IgG antibodies.

CONFIDENTIAL

Collect 12.5ml blood samples of all the subjects and separate serum. Nentralizing antibody will be determined by microdose cytopathogenic efficiency (CPE) assay and specific IgG antibody will be tested by enzyme-linked immunosorbent assay (ELISA).

Time points of blood sample collection is described in Table 1 Sample Size and Procedures of Clinical Trial.

Table 1 Sample size and Procedures of Clinical Trial

Sequential Vaccination Time of Recombinant Vaccine	Booster Immunization	Sequential Vaccine Group	Sample Size	Safety	Blood Collection Time Point
1-3 months after 2 doses	31-90 days	Recombinant Vaccine	300	Collected solicited AEs within 0-7 days and non-solicited AEs within 8-30 days after immunization with sequential booster dose, and SAEs up to 6 months following immunization with the booster dose.	Before the sequential dose ①, 14 days②, 28 days ③, 3 months ④, 6 months ⑤, 9 months ⑥, 12 months ⑦after sequential vaccination of one dose.
		Inactivated Vaccine	300		
4-6 months after 2 doses	91-180 days	Recombinant Vaccine	300		
		Inactivated Vaccine	300		
≥ 6 months after 2 doses	≥ 181 days	Recombinant Vaccine	300		
		Inactivated Vaccine	300		
Total sample size			**1800**		

Note:

1) Blood collection window at 14 days, 28 days is +10 days, blood collection window at 3M, 6M, 9M, 12M is +30 days.

2) If the blood sample test results show that the antibody level is too low after the full immunization, blood collections at subsequent time points will not be carried out.

CONFIDENTIAL

4.3. Study Endpoint

4.3.1 Immunogenicity Endpoint:

Primary Endpoints:

GMT and 4-fold rise of anti-SARS-CoV-2 neutralizing antibody 14 days after sequential vaccination of one booster dose;

GMT and 4-fold rise of anti-SARS-CoV-2 neutralizing antibody 28 days after sequential vaccination of one booster dose.

Secondary Endpoints:

GMT of anti-SARS-CoV-2 IgG antibody, 4-fold rise, GMI and proportions of neutrializing antibody titiered ≥ 1: 16, ≥ 1: 32 and ≥ 1: 64 before vaccination and 28 days after vaccination of sequential booser dose.

4.3. 2 Safety Endpoints:

The incidence of any adverse reactions/events observed within 30 minutes after vaccination;

The incidence of adverse reactions/events observed within 0 ~ 7 days and 8 ~ 30 days after vaccination.

The incidence of SAEs observed up to 6 months after vaccination and full immunization.

4.3.3 Immune Persistence Endpoints:

GMT of anti-SARS-CoV-2 neutralizing antibody and IgG antibody, the proportions of neutralizing antibody titered ≥ 1: 16, ≥ 1: 32 and ≥ 1: 64 at 3 months, 6 months, 9 months and 12 months after full course of immunization,

4.3.4 Exploratory Endpoints:

The vaccine efficay of recombinant COVID-19 vaccine (CHO cells) against COVID-19, severe cases and deaths after 14 days following sequential vaccination in population aged 18 years and above.

4.4 Trial Hypothesis

① The 4-fold rise of neutralizing antibody of recombinant COVID-19 vaccine (CHO cells) after sequential vaccination with one dose of vaccine is not inferior to that of inactivated COVID-19 vaccine (Vero cells), and the threshold of non-inferiority is-10%. If the lower bound of 95% CI is >-10%, the hypothesis of non-inferiority in 4-fold rise between vaccine and control groups will be concluded.

② After sequential vaccination of one dose of recombinant COVID-19 vaccine (CHO cells),

CONFIDENTIAL

GMT is not inferior to COVID-19 inactivated vaccine (Vero cells), and the non-inferior threshold is 2/3 (GMT ratio; the difference on log10 scale is-0.176). If the lower bound of GMT ratio 95% CI is > 2/3, the hypothesis of non-inferiority in GMT between vaccine and control groups will be concluded.

4.5 Safety Observation Indicators and Grading Criteria

4.5.1 Safety Observation Indicators

(1) Adverse events collected in 0-7 days

Local adverse events: pain, induration, swelling, rash, redness and pruritis.

Systemic adverse events: fever, diarrhoea, constipation, dysphagia, anorexia, vomiting, nausea, myalgia(systemic), arthralgia, headache, cough, dyspnoea, systemic pruritis (no skin damage), abnormal skin mucosa, acute allergic reactions, fatigue, and dizziness

Other adverse events: any medical events other than the above mentioned during clinical studies, such as acute diseases, accidental injuries, etc.

(2) Adverse events collected in 8-30 days

Any medical events, such as acute diseases and accidental injuries occurred during 8~30 days in clinical studies.

(3) Pregnancy events

Collect all pregnancy events and outcomes between vaccination and end of the study (6 months after full course of vaccination). Follow-up of the new-borns should last at least 30 days after birth.

4.5.2 Adverse Event Grading Criteria

Local adverse events, systemic adverse events, vital signs, and laboratory testing parameters after vaccination were determined based on the *Guiding Principles of Adverse Events Grading Criteria for Clinical Trials of Prophylactic Vaccines*.

Table 2 Local Adverse Events Grading Table

Symptoms/signs	Grade 1	Grade 2	Grade 3	Grade 4
Pain	Do not or slightly affect physical activities	Affect physical activities	Affect daily life	Loss of basic self-care ability, or hospitalization
Induration *, swelling * * #	2.5 ～ < 5 cm in diameter or 6.25 ～	5 ~ <10cm in diameter or 25 ～ < 100cm^2 in	Diameter ≥10 cm or area ≥100 cm^2 or	Abscess, exfoliative

	< 25 cm^2 in area not or slightly affecting daily life	area or affecting daily life	ulceration or secondary infection or phlebitis or sterile abscess or wound drainage or seriously affect daily life	dermatitis, dermal or deep tissue necrosis
Rash *, flush * * #	2.5 ~ < 5 cm in diameter or 6.25 ~ < 25 cm^2 in area not or slightly affecting daily life	5 ~ < 10 cm in diameter or 25 ~ < 100 cm^2 in area or affecting daily life	Diameter ≥10 cm or area ≥100 cm^2 or ulceration or secondary infection or phlebitis or sterile abscess or wound drainage or serious affect daily life	Abscess, exfoliative dermatitis, dermal or deep tissue necrosis
Pruritus	Itching at the vaccination site was relieved by itself or within 48 hours after treatment	Itching at the vaccination site did not relieve within 48 hours after treatment	Affect daily life	NA

Note: * In addition to measuring the diameter directly for grading evaluation, the progress and change of measurement results should also be recorded.

** Maximum measured diameter or area shall be used.

The evaluation and grading of induration and swelling, rash and redness should be based on the functional grading and actual measurement, with higher grading be selected.

When judging severity, it can be judged as a next grade adverse event only when it meets the grading criteria of the previous one.

Table 3 Systemic Adverse Events Grading Table

Physical Sign	Grade 1	Grade 2	Grade 3	Grade 4
Fever * (axillary temperature ℃)	37.3 ~ < 38.0	38.0 ~ < 38.5	38.5 ~ < 39.5	≥ 39.5 for more than 3 days
Diarrhea	Mild or transient, 3 ~ 4 times/day, abnormal fecal characteristics, or mild diarrhea lasting less than 1 week	Moderate or persistent, 5 ~ 7 times/day, abnormal fecal characteristics, or diarrhea > 1 week	> 7 times/day, abnormal fecal characteristics, hemorrhagic diarrhea, orthostatic	Hypotensive shock, requiring hospitalization

CONFIDENTIAL

			hypotension, and electrolyte imbalance, requiring intravenous infusion > 2L	
Constipation *	Need fecal softener and dietary adjustment	Need laxative drugs	Stubborn constipation requires manual dredging or enema	Toxic megacolon or intestinal obstruction
Dysphagia	Mild discomfort when swallowing	Restricted diet	Diet and conversation are very limited; can't eat solid food	Can't eat liquid food; Need intravenous nutrition
Anorexia	Loss of appetite, but no reduction in food intake	Loss of appetite, food intake decreased, but body weight did not decrease significantly	Loss of appetite and obvious weight loss	Need measures to intervene (e.g., gastric tube feeding, parenteral nutrition)
Vomiting	1 ~ 2 times/24 hours without affecting activities	3 ~ 5 times/24 hours or limited activity	> 6 times within 24 hours or intravenous rehydration is required	hospitalization or other nutrition due to hypotensive shock
Nausea	Transient (< 24 hours) or intermittent and normal food intake	Persistent nausea leads to reduced food intake (24 ~ 48 hours)	Persistent nausea leads to almost no food intake (> 48 hours) or need for intravenous rehydration	Life Threatening
Myalgia (local)	not affect daily activities	Slightly affect daily activities	Severe myalgia, seriously affects daily activities	Emergency or hospitalization
Arthralgia	Mild pain without hindering function	Moderate pain, need analgesics and/or pain interferes with function, but does not affect daily activities	Severe pain, need for painkillers and/or pain affects daily activities	Disabling pain
Headache	not affect daily activities and not need treatment	Transient, slightly affect daily activities and may require treatment or intervention	Seriously affect daily activities and need treatment or intervention	Intractable, requiring emergency or hospitalization

CONFIDENTIAL

Coughing	Transient, without treatment	Persistent cough, effective treatment	Paroxysmal cough, treatment uncontrollable	Emergency or hospitalization
Dyspnea	Dyspnea during exercise	Dyspnea in normal activity	Difficulty breathing at rest	Difficulty breathing, needing oxygen therapy, hospitalization or assisted breathing
Pruritus at non-vaccination site (No skin damage)	Slight itching, not or slightly affects daily life	affects daily life	Itching makes it impossible for daily life	NA
Abnormal Skin Mucosa	Erythema/pruritis/color	Diffuse rash/maculopapular rash/dryness/desquamation	Blister/exudation/desquamation/ulcer	dermatitis involves mucosa, or erythema multiforme, or is suspected of Stevens-Johnson syndrome
Acute Allergic Reaction * *	Local urticaria (blister) without treatment	Local urticaria, requiring treatment or mild angioedema, requiring no treatment	Extensive urticaria or angioedema requiring treatment or mild bronchospasm	Anaphylactic shock or life-threatening bronchospasm or laryngeal edema
Fatigue	Not affect daily activities	Affect daily activities	Seriously affect daily activities and cannot work	Emergency or hospitalization

Note: * Axillary temperature is usually used in China, tympanic temperature/temporal artery temperature = axillary temperature +0.5 ℃. When persistent high fever occurs, the cause of high fever should be determined as soon as possible;

* For constipation, attention should be paid to the changes before and after vaccination;

When judging severity, it can be judged as a next grade adverse event only when it meets the grading criteria of the previous one.

CONFIDENTIAL

For clinical abnormalities not covered in the above table, the severity grading evaluation of adverse events should be carried out according to the following criteria.

Grade 1	Grade 2	Grade 3	Grade 4	Grade 5
Mild: Short-term (< 48h) or slight discomfort, which does not affect activities and does not need treatment	Moderate: Mild or moderate activity restriction, which may require medical treatment, no or only mild treatment	Severe: obvious activity Restricted, need to see a doctor and receive treatment, and may need to be hospitalized	Critical: It may be life-threatening, severely limited in activities, and needs monitoring and treatment	Death

4.5. 3 Relationship between Adverse Events and Investigational Vaccines

Impossible: Adverse events occur due to other factors, and there is sufficient evidence to prove that adverse reactions/events are caused by other reasons and unrelated to d vaccination.

Unlikely: The occurrence of adverse events may be caused by other factors, such as the clinical status of the subject, other treatments or accompanying medication, which are inconsistent with the known adverse reactions of vaccination.

Possible: Adverse events are consistent with known investigational vaccine information, have a reasonable temporal sequence with vaccination, and/or have occurred for vaccination. There is also a causal relationship with the investigational vaccine, but it may also be related to other factors.

Likely: Adverse events are consistent with the known investigational vaccine information and have causal relationship with the investigational vaccine, and cannot be explained by other factors, such as the clinical status of the subject, other treatments or concomitant medication.

Definite: Adverse events are consistent with the known investigational vaccine information and have a causal relationship with the investigational vaccine, and this relationship cannot be explained by other factors, such as the clinical status of the subject, other treatments or accompanying drugs. In addition, adverse events occurred repeatedly when the subjects used the investigational vaccine again.

4.5.4 Adverse Event of Special Interest

According to the CIOMS VII definition, AESI refers to a class of scientific and medical events (including serious or non-serious) related to investigational vaccines or projects, which require investigators to continuously monitor and quickly communicate with the sponsor. These events need further study to understand their characteristics; Depending on the nature of the event,

CONFIDENTIAL

the sponsor also needs to communicate quickly with other relevant parties (such as regulatory authorities).

AESI related to this vaccine include: **nervous system diseases** (generalized convulsions, Guillain-Barre syndrome, acute disseminated encephalomyelitis, aseptic meningitis, general convulsions, peripheral facial paralysis, loss of smell and taste), immune system diseases (vaccine-related respiratory diseases increase, anaphylactic shock, multisystem inflammatory syndrome in children), **respiratory diseases** (acute respiratory distress syndrome), **cardiac system diseases** (acute cardiovascular injury (including myocarditis, pericarditis, arrhythmia, heart failure, myocardial infarction), blood system damage (thrombocytopenia, coagulation dysfunction (including coagulation diseases, thrombosis, thromboembolism, internal/external bleeding, stroke)), **kidney system diseases** (acute kidney injury), liver system diseases (acute liver injury) and **skin system diseases** (chilblain-like lesions, single organ skin vasculitis, erythema multiforme, alopecia). The follow-up period of AESI is 6 months after the full course of immunization.

4.6 Randomization and Blinding

4.6.1 Randomization

Stratified block randomization method will be employed in the study to perform stratifications according to sequential vaccination time (1-3 months, 4-6 months, and ≥6 months after 2 doses). Interactive Web Response System (IWRS) will be used in subject randomization and vaccine allocation.

The randomization statistician will produce subject randomization list and vaccine randomization list by SAS 9.4 (ot later version), which will be imported into IWRS by system engineer. After one subject is sreened successfully, the investigator at each center will log onto IWRS to get randomization number of the subject. Before vaccination, the investigator will receive the vaccine number from IWRS and inoculate vaccines accordingly. If the vaccine is damaged, the investigator could acquire a new vaccine number from IWRS and inoculate the vaccine with new vaccine number.

4.6.2 Blinding

Before the start of the study, the staffs from sponsor who will not participate in the clinical trial will blind the vaccines to be used in the study together with the unblinded randmization statistician. The vaccine label will be sticked on the specified position of each vaccine according to vaccine randomization list. The randomization statistician will supervise and guide the process of blinding. After blinding is completed, the vaccine randomization list will be sealed by the unblind randomization statistician. The whole blinding process will be recorded and written in a

CONFIDENTIAL

document named blinding records, which is one of the important documents of this trial. The blinding personnel cannot participate in other related works of the trial, and they are not allowed to tell any information to any personnel who participats in the trial.

The subject randomization list and vaccine randomization list will be sealed, including randomization number, vaccine number and the corresponding group assignment, together with the randmozation seed number. They will be well sealed and saved until the database is locked.

4.6.3 Emergency Blind Code Breaking

In an emergency, when the investigator thinks that the group assignment information of one subject is beneficial for the handling of adverse events, it will be broken through the Emergency Blind Code Breaking Module in IWRS. The investigators need to discuss with the Sponsor and both shall reach agreement before performing emergency blind code breaking.

5. SCREENING AND WITHDRAWAL OF SUBJECTS

The subjects must fulfill all eligibility criteria to be involved in this study. No exemptions from any in-/exclusion criteria will be allowed. If any deviation from eligibility is retrospectively detected for an already randomized subject, the investigator and sponsor must determine immediately the safety to treat this subject further within the study.

5.1 Subject Population

5.1.1 Inclusion Criteria

- Age range: populations aged 18 years and above;
- Judged by the investigator that the health condition is well after inquiry and physical examination;
- Vaccinated with 2 doses of CNBG inactivated COVID-19 vaccine according to the product insert;
- Female subjects who are not nursing or pregnant at the time of enrolment (negative urine pregnancy test) and have no family planning within the first 6 months after enrollment. Effective contraceptive measures have been taken within 2 weeks before inclusion.
- During the whole follow-up period of the study, be able and willing to complete the whole prescribed study plan;
- With self ability to understand the study procedures, the informed consent & voluntarily sign an informed consent form and is able to comply with the requirements of the clinical study protocol.

5.1.2 Exclusion Criteria

- Confirmed cases, suspected cases or asymptomatic cases of COVID-19;

CONFIDENTIAL

- With a history of SARS and MERS infection (self-report, on-site inquiry);
- Has vaccinated with any other vaccines rather than one or more doses of Sinopharm CNBG inactivated COVID-19 vaccine;
- Axillary temperature ≥ 37.3 ℃ (forehead temperature ≥ 37.8 ℃);
- Previous severe allergic reactions to vaccination (such as acute allergic reactions, urticaria, dyspnea, angioneurotic edema or abdominal pain)
- Allergy to known components of recombinant COVID-19 vaccine (e.g., aluminum and histidine);
- Severe respiratory diseases, severe liver and kidney diseases, hypertension (systolic blood pressure ≥ 150 mmHg, diastolic blood pressure ≥ 90 mmHg), diabetic complications, malignant tumors, various acute diseases or acute attacks of chronic diseases;
- Has been diagnosed with congenital or acquired immunodeficiency, HIV infection, lymphoma, leukemia or other autoimmune diseases;
- Has a history of convulsions, epilepsy, encephalopathy, long-term alcohol and drug abuse, history of thyroidectomy, history of infectious diseases, mental illness or family history (direct);
- With Congenital malformation or developmental disorder, genetic defect, severe malnutrition, etc.;
- Has a history of coagulation dysfunction (e.g. coagulation factors deficiency and coagulation diseases);
- Asplenia or splenectomy, functional asplenia caused by any situation;
- Receving anti-TB (tuberculosis) therapy;
- Received immunoenhancement or inhibitor therapy within 3 months (continuous oral or IV administration for more than 14 days);
- Received other vaccines within 14 days before vaccination;
- Received blood products within 3 months before vaccination;
- Received other investigational drugs within 6 months before vaccination;
- Plans to move before the end of the study or leave the local area for a long time during the scheduled study visits;
- Other circumstances judged by investigators that are not suitable for this clinical trial.

5.1.3 Criteria for Early Withdrawal from the Trial

Early withdrawal means that the subjects fail to complete vaccination and blood collection

CONFIDENTIAL

according to the protocol, and the investigators decide whether to continue the follow-up research according to the situation. In any of the following situations, the subject will withdraw from the clinical trial in advance.

- Subjects asked to withdraw from clinical trials;
- Intolerable adverse events, whether related to the investigational drug or not
- The health status of the subjects does not allow them to continue to participate in this trial.
- Subjects are vaccinated with other investigational vaccines during the study period.
- Any reasons the investigator deems necessary.

5.1.4 Criteria for Clinical Trial Suspension and Termination

In case of any of the following circumstances, the clinical trial shall be suspended or terminated, and the investigator, the sponsor and the ethics committee shall jointly decide whether to terminate the clinical trial in advance:

- After vaccination, the number of subjects with adverse reactions with severity of Grade 3 exceeds 15% of the number of subjects vaccinated in this subgroup.
- One case of Grade 4 adverse reaction or SUSAR was confirmed to be related to vaccination.

In case of any of the following circumstances, the clinical trial shall be terminated:

- The Sponsor found that the vaccine has potential safety hazard, or the study has quality problems and requires the complete termination of the trial.
- The Ethics Committee requests termination of the trial because of the ethical violation in the study.
- The administrative authority requests termination of the trial.

6. PRODUCT INTRODUCTION

6.1 Description and Characteristics of the Investigational Products

Recombinant COVID-19 vaccine (CHO cells) is a recombinant protein expressed by recombinant Chinese hamster ovary (CHO) cells and designed based on the receptor binding domain (RBD) of S protein of SARS-CoV-2. Engineered cells were cultured, harvested and purified by a series of processes, and then added with aluminum hydroxide adjuvant.

This product is a sterile formulation, packed in vial with a specification of 0.5 ml/dose/vial. After shaking, it is an opalescent suspension, which can be layered due to precipitation and easily shaken away without lumps.

The main active ingredient of this product is the recombinant protein formed by the receptor

CONFIDENTIAL

binding region of SARS-CoV-2 spike protein. Excipients include sodium chloride, aluminum hydroxide adjuvant, histidine, etc. Each dose contains 20μg of recombinant protein, 4.38 mg of sodium chloride, 0.39 mg of histidine, 0.30 mg of aluminum and water for injection. Used to prevent diseases caused by SARS-CoV-2.

6.1.1 Investigational Vaccine

Investigational Vaccine: Recombinant COVID-19 Vaccine (CHO cell)

Manufacturers: Sinopharm CNBG National Vaccine and Serum Institute (NVSI), Lanzhou Institute of Biological Products Co., Ltd. (LIBP)

Specifications: 0.5 ml/vial per human use, which contains 20ug of viral protein antigen.

Storage Condition: 2-8 ℃.

Vaccine Batch Number: 20201113

Expiry Date: Nov 11, 2022

Control vaccine: Inactivated COVID-19 Vaccine (Vero cell)

Manufacturer: Beijing Institute of Biological Products Co., Ltd.

Specifications: 0.5 ml/vial per human use, which contains 6.5U of antigen.

Storage conditions: 2-8 ℃

Vaccine Batch Number: 2021071710.

Expiry Date: Jul 8, 2023

6.1.2 Production Process

The recombinant COVID-19 vaccine (CHO cells) developed in this project takes CHO cells as the express system and SARS-CoV-2 spike protein as the target, applying genetic engineering technology to construct and screen stable cell lines with recombinant expression protein. After culture, harvesting and a series of purification processes and adding aluminum hydroxide adjuvant, the vaccine is finished after filling.

6.2 Vaccine Packaging and Labeling

All investigational vaccines are packaged in doses in boxes with the same appearance and only marked by the vaccine number. Each study ID will be matched with a unique vaccine number, the format of the vaccine number is AE+4-digit Arabic number. See the following label for detailed information.

CONFIDENTIAL

6.2.1 Vaccine Labeling

(1) Each dose of vaccine is packaged separately and the vaccine number is sticked onto.

(2) After the vaccine is put into use, the initials of the subject and the vaccination date shall be filled on the outer package.

Clinical Trial Labeling Illustration

Outer Packaging Box Label

**Recombinant COIVD-19 Vaccine (CHO cell)**
**For Clinical Use Only**
**Vaccine Number：AExxxx**
**Initials：　　Vaccination Date：　YY　MM　DD**
**Storage at 2-8℃, 0.5mL/dose**
**Batch Number：**

Vaccine Label:

**Recombinant COVID-19 Vaccine(CHO cell)**
**（For Clinical Use Only）**
Vaccine Number：AExxxx
Batch Number：XXXX

6.3 Vaccine Storage and Transportation

Vaccines should be stored and transported at 2 ~ 8 ℃ away from light to prevent freezing. The temperature during vaccine transportation and storage shall be dynamically monitored and recorded. If the storage and transportation temperature conditions exceed the specified range, the on-site investigators should immediately contact the personnel of the responsible clinical institutions and the sponsors to decide whether the vaccine can be used.

The management of receiving, keeping, formulation, recovery, returning/destroying of the investigational vaccine shall conform to the requirements of relevant laws and regulations.

6.3.1 Vaccine Transportation

CONFIDENTIAL

The whole process of vaccine management should meet the cold chain requirements, and there should be vaccine transportation and storage conditions that meet the requirements of the protocol. During the vaccine transportation, there should be a transportation sheet and temperature monitoring. Upon arrival, the packaging condition and unpacking temperature should be recorded. After the recipient receives the vaccines, the transportation sheet should be signed, faxed, or copied to the sender. Both parties should properly keep the transportation sheet.

6.3.2 Vaccine Storage and Distribution

The investigational vaccines should be kept in a separate area, locked in dedicated cabinet and managed by dedicated personnel. Vaccine recipient must verify and record the batch number, expiration period and delivery status of vaccines, establish work forms for vaccine handover, registration, use and recovery, fill in them as required, and keep them in the work records.

The trial vaccine shall not be used in non-clinical trial population.

6.3.3 Vaccine Records

Vaccine handover records: The sponsor will provide investigational vaccines, control vaccines (placebo) and vaccine handover sheet, and investigator will verify the name, batch number and quantity of the vaccine upon receiving.

Vaccine registration and use records: the investigator shall establish vaccine registration and use records and distribute investigational vaccine and control vaccines according to the number of people in the observation groups.

Vaccine recycle record: The abandoned, expired and the remaining vaccines in this trial are returned to sponsor. Sponsor receives vaccines and verifies the batch number and quantity of vaccines, fills in the vaccine handover form, and makes relevant records, which are signed by vaccine manager and sponsor representatives

6.4 Administration and Immunization Schedule

Route of Administration: lateral deltoid muscle of either upper arm, intramuscular injection.

Vaccination Procedure: Among the population who had completed two doses of inactivated COVID-19 vaccines according to the 0 and 21 days (+7 days) immunization schedule, one dose of recombinant COVID-19 vaccine (CHO cells) or inactivated COVID-19 vaccines (Vero cells) will be inoculated at different time points of 1~3 months, 4~6 months, and ≥ 6 months for sequential immunization

7. STUDY WORKFLOW AND VISITING METHOD

7.1 Schedule of Visits

Table 4 lists the assessment items and procedures according to the phase of the clinical stuides and visit.

7.2 Study Procedure

7.2.1 Screening Period, Baseline Period, and Vaccination Procedure (D0)

The first visit was conducted according to the following procedure and evaluation processes.

✓ **Recruitment**

The investigators will jointly publicize this clinical trial recruitment notice to qualified volunteers/guardians, examined, and approved by the Ethics Committee, with local medical staff, and recruit and register candidate subjects based on the principle of voluntary participation. During the study, the recruitment should be adjusted in real time progress, to ensure that the gender and age of the participants are relatively balanced

✓ **Informed Consent**

Informed consent refers to the voluntary participation of subjects in clinical studies. Prior to the commencement of any test-related procedures (any genetics-related sample collection, testing, etc.), a written informed consent must be obtained from the subject.

After arriving at the research site, the subjects first give informed consent. The investigators inform the subjects of the informed consent form of this clinical trial orally and in writing, including study purpose, procedure, duration, risks and benefits, etc. Under the condition of voluntary participation, the subjects and research doctors signed an informed consent form together. The informed consent form shall be made in duplicate, and the guardian or principal of the volunteer shall keep a copy, and the original shall be kept at the study site.

Collect contact information (address, e-mail, landline, and mobile phone numbers, etc., if applicable) of subjects and their families.

✓ **Physical Examination, Inclusion/Exclusion Criteria**

Volunteers who signed the informed consent were given height, weight, body temperature, blood pressure (aged 18 and above), cardiopulmonary auscultation, skin examination. Within 10 minutes before the temperature test, the subjects are forbidden to eat or drink anything hot or cold, or smoke.

According to the "inclusion criteria" and "exclusion criteria", the investigators inquire, understand, and check the vaccination records of the subjects, that is, the past medical history, and

CONFIDENTIAL

judged whether the subjects should be enrolled in this clinical trial according to the inquiry results.

- ✓ **Study Number Assignment**

Eligible subjects are assigned unique study numbers in sequence. Once the study number is assigned, it cannot be reassigned to other subjects.

- ✓ **Blood Sample Collection before Vaccination**

Women of childbearing age (from menarche to menopause) need to collect about 2ml urine samples for urine pregnancy test before vaccination.

About 12.5 ml of blood samples are collected on an empty stomach for SARS-CoV-2 antibody detection after recruitment.

Nasopharyngeal swabs are collected for PCR detection to establish antibody baseline and PCR baseline for booster inoculation. After sample collection, the subjects can enter the following process.

- ✓ **Vaccination**

Subjects can be vaccinated after sample collection. Before vaccination, it is necessary to check the information of subjects and the investigational vaccines, obtain vaccines with corresponding numbers and open the outer package. After the vaccine label and outer package label are checked, fill in the initials of the vaccinee's name and vaccination date on the label of the outer package.

Vaccination site is at the lateral deltoid muscle of upper arm, in principle, the non-habitual arm of the subject.

During vaccination, the investigational vaccine and control vaccine should be kept at 2 ~ 8 ℃ (the temperature should be monitored and recorded every 1 hour), and it should take no more than 30 minutes from the time the vaccine is taken out of the heat preservation container to the completion of vaccination.

During vaccination, in case of abnormal color, damage, insufficient loading or precipitation, it is necessary to stop vaccination immediately, report to the sponsor, auditor and on-site person in charge, discard the original vaccine according to the procedure after confirming the damage of the vaccine, and obtain a new vaccine number in the system by the distribution principle of the central randomized system after approval.

Record the injection site (right arm or left arm) and vaccination information.

CONFIDENTIAL

✓ **On-site Medical Observation**

Observe the immediate adverse reactions 30 minutes after vaccination. Doctors explain on-site adverse reaction judgment, measurement methods, recording methods, precautions, reporting methods, etc. Diary cards, rulers and thermometers are distributed and train subjects how to use thermometers correctly, observe adverse events and fill in diary cards within 0-7 days after vaccination, and inform them of the time to make an appointment to return the diary cards (return the completed diary cards on Day 14 Visit 4).

Ask the subjects to try to take temperature measurements at the same time every day within 7 days after vaccination.

Before leaving the study site, the vaccination site and body temperature of the subjects should be checked, and the observed adverse events should be evaluated and recorded in the electronic system.

Inform the subjects that they should take the initiative to provide any adverse reactions and accompanying medication. If there is any reaction after vaccination, it is necessary to inform the study center in time. PI or qualified SI will evaluate them, provide the subjects with correct response plan, and ask them to return to the clinic for immediate evaluation if necessary

.

7.2.2 Safety Visit Procedure (Telephone Follow-up)

Telephone follow-ups should be performed and evaluated according to the following procedures:

✓ Telephone follow-up subjects confirms that they have completed the diary card as required (until Visit 4).

✓ Evaluate whether the subjects are contraceptive and adhere to the lifestyle that meets the study requirements, evaluation the subjects' willingness and ability to conform to the study requirements.

✓ Evaluate and record any adverse events since the previous visit.

7.3 Safety Observation and Follow-up

7.3.1 Safety Observation

(1) Observe the site for 30 minutes after vaccination to collect local and systemic adverse events.

(2) Telephone follow-up once in 6-24 hours after vaccination, within 1-3 days and 4-7 days respectively, the subjects were followed-up by telephone and guided to observe the adverse events

CONFIDENTIAL

correctly and fill in the diary card.

(3) On the 14th day (Visit 4), the investigator reviews the filling of the diary card during this period and re-collect the diary card.

(4) On the 8th -30th day after vaccination, the investigator uses telephone follow-up once a week, combined with active report of the subjects to monitor the safety.

(5) When it is known that the subject has adverse reactions/events of Grade 3 or above, a face-to-face visit should take place within 24 hours.

(6) SAEs and AESIs are observed from the first dose to 6 months after the full course of vaccination by means of monthly telephone follow-up and self-reports from the subjects.

Note: Subjects should report respiratory tract infection symptoms such as fever and cough. Investigators should make comprehensive judgments, carefully identify whether it is common solicitation symptoms after vaccination or infection caused by other reasons, and closely observe the disease progress. Once the disease worsens, report it to the person in charge of the site quickly, and start the green treatment channel quickly to ensure that the subjects get timely diagnosis and treatment.

7.3.2 Safety Information Record

After vaccination, all subjects should complete the diary card. Investigators will ask subjects about adverse events during on-site visits and telephone follow-up after vaccination (except for scheduled/unscheduled visits to the research center). If the subject has persistent local or systemic reaction within 0-30 days after vaccination, it should be continuously recorded and reexamined until the symptoms are relieved.

7.4 Laboratory Testing

Collect laboratory samples (including blood samples) after physical examination, and complete sample collection before vaccination.

The sampling time and arrangement of laboratory testing samples during the study period are shown in the testing flow chart, and the samples are tested and analyzed by the central and/or local laboratories.

Pregnancy Sample Collection

All female subjects of childbearing age need to collect urine/blood samples for pregnancy test, and the test results will be used for screening.

Nucleic Acid Sample Collection

All subjects need to collect nasopharyngeal swabs for RT-PCR detection at the same time of recruitment, and the test results will be used for subsequent analysis.

CONFIDENTIAL

Blood Sample Collection before Immunization (D0)

After recruited, all subjects collect about 12.5 ml venous blood before vaccination, separate the serum within 24 hours, package into multiple test tubes (each tube is not less than 0.5 ml), and store at-20 ℃ or below for antibody detection.

Blood Sample Collection after Immunization

About 12.5 ml of venous blood was collected from all subjects at 14 days, 28 days, 3 months, 6 months, 9 months and 12 months after immunization, and the serum was separated within 24 hours and packed into 2 tubes (each tube is not less than 0.5 ml), which were stored at-20 ℃ and below for immunogenicity neutralizing antibody detection.

Blood Sample Collection of Cases (Suspected or Confirmed)

After the subjects develop typical symptoms of SARS-CoV-2 infection, about 4 ml of venous blood is collected for antibody detection, and the serum is separated and packed into 2 tubes (each tube is not less than 0.5 ml) within 1-3 days. In addition, about 10ml venous blood is collected, and cytokines, cellular immunity and other detection items are carried out according to local medical conditions, laboratory clinical level. Serum is stored at -20 ℃ and below (antibody detection is carried out for subjects with confirmed and suspected cases).

Blood Sample Numbering Rule

Before the sequential dose: study number – 0

14 days after vaccination (D14+10 days) study number – 1

28 days after vaccination (D28 + 10 days) study number -2

3 months after vaccination (D90 + 30 days) study number-3

6 months after vaccination (D180 + 30 days) study number -4

9 months after vaccination (D270 + 30 days) study number -5

12 months after vaccination (D360 + 30 days) study number-6

Suspected and confirmed cases are coded in P1, P2, P3…according to the order of blood collection.

Table 4 Schedule of Events

Visit number	V1	V2	V3	V4	V5	V6	V7	V8	V9
Date (Security Mode)	D0<br>👓	D3<br>☎	D7<br>☎	D14<br>👓	D28<br>👓	3M after V1<br>👓	6M after V1<br>👓	9M after V1<br>👓	12M after V1<br>👓
Visit (window +/-days)<br>The subsequent visit is postponed according	0D	-2D	-3D	+ 10D	+ 10D	+ 30D	+ 30D	+ 30D	+ 30D
**SCREENING/BASELINE**									
Study informed consent [1]	✓								
Inclusion/Exclusion criteria evaluation	✓								
Demographics	✓								
Medication history	✓								
Contraception [2]	✓	✓	✓	✓	✓	✓	✓	✓	✓
IWRS Registration	✓								
**INTERVENTION**									
Vaccination	✓								
**SAFETY ASSESSMENT**									
Vital signs (blood pressure, body temperature, heart rate)	✓								
Height, Weight	✓								
Physical Examination	✓								
Observation at site after vaccination [3]	✓								
Telephone follow-up after vaccination	✓	✓	✓			Once per month within D28-6M			
Concomitant Medication	✓	✓	✓	✓	✓	✓	✓		
Adverse events	✓	✓	✓	✓	✓	✓	✓		
SAE	✓	✓	✓	✓	✓	✓	✓		
Diary Card [4]	✓			✓					
**LABORATORY TESTING**									
COVID-19 PCR[5]	✓								
Neutralizing Antibody Test	✓			✓	✓	✓	✓	✓	✓
Specific IgG Antibody Test (anti-N, anti-S protein)	✓			✓	✓	✓	✓	✓	✓

CONFIDENTIAL

1. Consent process completed, and form signed before any study-related procedures are conducted.

2. Urine/blood pregnancy test must be completed within 24 hours prior to vaccination for women of childbearing potential, and only negative test results are eligible for enrollment.

3. All subjects should be under observation for at least 30 minutes after vaccination.

4. Paper diary cards will be used for safety information records.

5. PCR testing could be performed any time during the study when the subject developed any suspected symptoms.

CONFIDENTIAL

7.5 Concomitant Medication

Within 0-30 days, actively follow up and observe the local and systemic adverse events of the subjects and record them on the vaccination diary card. At the same time, it is necessary to record the drug used of the subjects in the vaccination diary card.

Medication Allowed: During the clinical trial, if the subject has adverse events, necessary medication and medical treatment should be allowed.

Vaccines Allowed: Other vaccines are allowed during the trial, which should be carried out 14 days after the trial vaccination. Emergency vaccination, such as rabies vaccine and tetanus vaccine, is not restricted.

Medication Record: To understand the influence of drug use during the trial on vaccine safety and immunogenicity, and to collect adverse events that may be related to vaccination without omission, investigators should instruct the guardians of the subjects to record the medical treatment and medication in diary cards as much as possible and assist in collecting the hospitalization medication records of the subjects during the observation period. When SAE occurs, copies of corresponding medical records and medication records should be collected and kept. The following drugs need to be transcribed into the electronic case report form (eCRF).

(1) Hormone/steroid drugs and other immunosuppressants.

(2) Antiallergic Drugs

(3) Antipyretics/analgesics/NSAIDs

(4) Prophylactic Vaccines

(5) Therapeutical Medical Products

(6) Antibiotics

(7) Antiviral Drugs

(8) Chinese Patent Drugs

(9) Others

7.6 Criteria for Suspension or Early Termination

In case of any of the following circumstances, the trial shall be suspended, and the investigator, the sponsor and the Ethics Committee shall jointly hold a meeting to decide whether to terminate the clinical trial early:

- One case of Grade 4 adverse reaction related to vaccination or SUSAR in any group, including death or life-threatening SAE;
- The number of subjects with adverse reactions of severity Grade 3 or above

CONFIDENTIAL

exceeds 15% of the subjects vaccinated in this subgroup;

- DSMB evaluation of clinical research has high potential safety risks.

In case of any of the following circumstances, the clinical trial shall be terminated:

- Sponsor has found that the vaccine has potential safety hazards or the trial has quality related problems, and requiring the complete termination of the study.
- The Ethics Committee requests the termination of the trial because of the violation of ethics in the trial.
- Request by the administrative authority to terminate the trial

7.7 Protocol Violation and Deviation

7.6.1 Protocol Violations

The list of protocol violations is as follows (including but not limited to):

- Fail of obtain subject's informed consent;
- Subjects who did not meet the inclusion criteria or met the exclusion criteria were enrolled in the study;
- Subjects received wrong research interventions (such as vaccination errors);
- Fail to report serious adverse events (SAE) within specified time;
- Received other investigational vaccines during the study period.

CONFIDENTIAL

7.6.2 Protocol Deviations

The list of protocol deviations is as follows (including but not limited to):

- Not receive the investigational vaccine during the window period;
- Not collect blood during the window period;
- The interval between vaccination and other vaccines is not long enough, except for emergency vaccination such as rabies.

7.7 Study Duration

Eearly Subject Recruitment: 1 month;

Sequential Vaccination + immune persistence observation: about 18 months

Sample Testing: about 1 month

Complete the Summary Report: about 1 month

7.8 Site Closure

All subjects completed long-term safety observation for 6 months after vaccination and blood collection for 12 months after immunization. All original data are collated, entered, and verified and submitted to the statistician. After clarifying the data after blind audit by the statistician, the study site is closed.

8. SAFETY DEFINITION, REPORTING AND MONITORING

8.1 General Precautions

This clinical trial is a randomized, double-blind and controlled trial design.

Before the start of the trial, the sponsor shall strictly examine the trial site in accordance with GCP requirements, focusing on whether the environmental facilities of the trial site meet the requirements of "Vaccination Management Standards" and "Guiding Principles for Quality Management of Vaccine Clinical Trials". Ensure the first aid facilities and first aid equipment in the first aid room are complete and effective, and the first aid doctors have corresponding qualifications and capabilities. Emergency related personnel (emergency doctors, emergency nurses, ambulance drivers, etc.) are trained to be qualified and familiar with the transfer routes and procedures of the agreed hospital. They are on standby at the trial site during vaccination. The trial site shall be equipped with ambulances. The ambulance shall be parked in a fixed position to keep the vehicle in good condition and in an emergency and shall be under the command and transfer of the emergency response team at any time. During the vaccination period, the agreement hospital will make daily preparations for medical personnel, instruments and equipment, first aid drugs and first aid sites to ensure that the subjects can receive timely treatment. The trial site shall formulate an emergency plan, stipulate personnel responsibilities, contact numbers, rescue routes and other measures to ensure timely handling of unexpected adverse events, and ensure effective contact between subjects and investigators so that any adverse events can be reported and handled quickly.

CONFIDENTIAL

8.2 Risk Prevention Measures Related to COVID-19

(1) Trial site consideration

Investigators during recruitment should confirm whether there are new COVID-19 cases or suspected cases in the same village/community by investigating or inquiring volunteers. Whether fever, dry cough, fatigue, nasal obstruction, runny nose, sore throat, myalgia, diarrhea, shortness of breath and dyspnea occurred recently;

(2) Strengthen the management and personal protection of subjects during the study period

Disinfection of each functional area of clinical research should be strictly done in the test site according to regulations, and windows should be opened and ventilated regularly. Strictly implement independent areas and special passages, and the recipients and their

accompanying personnel wait in relative divisions to avoid contact with people other than research doctors and nurses; All functional areas and public places should be equipped with hand disinfectants and temperature measuring devices, and recipients and their accompanying personnel entering the clinical research site should be equipped with masks to disinfect their hands and measure their body temperature.

The trial site shall strictly disinfect each functional area of clinical research according to regulations, and regularly open windows for air-ventilation. Strict implementation of independent areas and special passages, recipients and their accompanying personnel waiting in relative zones, to avoid contact with research doctors, nurses and other personnel other than research; All functional areas and public places should be equipped with hand disinfectants and temperature measuring devices. Recipients entering the clinical research site and their accompanying personnel are required to be equipped with masks to disinfect their hands and measure their body temperature.

During the first dose of inoculation, the on-site investigators shall remind the subjects to strengthen their own protection. If COVID-19 pandemic occurs locally, the subjects shall be provided with necessary protective materials such as masks, alcohol, etc. in time, and close attention to the health status of the subjects shall be drawn, especially the symptoms related to COVID-19.

CONFIDENTIAL

(3) Detection of COVID-19 RT-PCR

Subjects need to be tested for COVID-19 RT-PCR before first dose vaccination. PCR tests should be performed before 2nd dose vaccination, 14 days after 2nd dose, 28 days after 2nd dose, and regularly afterwards at the sites with permitted conditions. Investigators need to regularly follow up and track the subjects to identify whether they have developed fever, dry cough, fatigue and other symptoms recently. If the above symptoms exist, the subjects will be collected samples for etiological detection of SARS-CoV-2 according to the prevention and protection policy for COVID-19 and based on the symptoms of the subjects, or are advised to go to the hospital for blood testing and radiographic examination. If there are no symptoms such as fever, dry cough and fatigue, the subject should be home isolated according to the requirements of COVID-19 prevention and control protocol.

8.3 Handling and Reporting of Serious Adverse Events

Serious adverse events refer to adverse medical events such as disability or loss of function, the need for hospitalization or prolonged hospitalization of subjects, and congenital abnormalities or birth defects.

Monitoring and reporting of adverse events in vaccine clinical trials are jointly completed by subjects, serious adverse event investigators, trial sites and responsible institutions at different observation time points with different stages.

The sponsor is the main body responsible for monitoring, evaluating and SAE reporting safety information of vaccine clinical trials. The person shall be designed as the administrator of clinical trial safety information monitoring and SAE reporting, and work with investigators to establish SOPs for clinical trial safety information monitoring and SAE reporting, know well the latest status of the safety information of the whole clinical trial, and timely report updates to all clinical trial institutions/ investigators and regulatory authorities.

If it is difficult to make a judgment on the correlation between SAE and vaccine or there is doubt about the judgment, when it is necessary to make a new judgment, the expert meeting shall make a judgment after argumentation.

(1) On-site Treatment Measures

Emergency plans for SAE treatment in clinical trials shall be established at the trial site, and all relevant personnel shall be trained. If the subjects show serious adverse events, the investigators shall immediately take appropriate measures for the subjects and record them. The investigators must take relevant approaches to know in time any clinically significant diseases/events related to vaccination. According to relevant national regulations, the subjects should be received appropriate treatment in time in the designated hospitals.

CONFIDENTIAL

Investigators at the trial site should follow up serious adverse events until the symptoms disappear or stabilize. The progress and outcome of all symptoms will be recorded in detail, and all drug treatments and medical treatments will be recorded at each follow-up. Investigators should truthfully record serious adverse events, which shall be evaluated and discussed in the final report after the test is completed or terminated.

During the whole observation process, if the subjects suffer from physical injuries caused by serious adverse reactions related to vaccination, which is confirmed by the expert investigation team, the local insurance company will give corresponding compensation.

(2) Reporting Procedures for Serious Adverse Events

- Reporting procedures of investigators

Any serious adverse event, whether or not related to the investigational vaccine, the

investigator must submit the initial report of the "Serious Adverse Event Report Form" to the drug administration department, the sponsor and the ethics committee by fax, e-mail or EDC system or personal delivery within 24 hours after learning about it.

Subsequently, the follow-up report of the "Serious Adverse Event Report Form" shall be submitted regularly until the end of the event. All information is reported in the "Serious Adverse Event Report Form" in the form of written reports, including description of adverse reactions/events, onset time and type, duration, intensity, causal relationship with vaccination, results, treatment methods (symptomatic treatment) and other relevant clinical and laboratory data.

When receiving the report of serious adverse events/reactions, the investigator shall, together with the sponsor, comprehensively consider the duration, scope, intensity, outcome and the willingness of the subject to decide whether the subject should continue to participate in the study or withdraw from the study in advance.

- Reporting procedure of sponsors

During the clinical trial of drugs, sponsors need to quickly report unexpected and serious adverse reactions (SUSAR) that are definitely related to or suspicious of the tested drugs in the form of case-by-case safety reports according to the Standards and Procedures for Rapid Reporting of Safety Data during Clinical Trial of Drugs. If the researcher and the sponsor cannot reach an agreement on the judgment of the causal relationship between adverse events and drugs, either party's judgment cannot exclude those related to the test drugs, and the sponsor should also make a quick report.

CONFIDENTIAL

For SUSAR that is fatal or life-threatening, the sponsor should report it as soon as possible after the first knowledge, but not more than 7 days, and report it within the following 8 days to improve the follow-up information (Note: the day when the sponsor first learned it is the 0th day). For SUSAR that is not fatal or life-threatening, the sponsor should report it as soon as possible after the first knowledge, but not more than 15 days. For other potential serious safety risk information, the sponsor should also report to the national drug evaluation agency as soon as possible, and at the same time make medical and scientific judgment on each situation. After the first report, the sponsor shall continue to track the serious adverse reactions and submit relevant new information or changes to the previous report in a timely manner in the form of a follow-up report. The reporting time limit shall be within 15 days from the date of obtaining the new information.

8.4. Outcome of Serious Adverse Events

The outcomes of serious adverse events include: (1) Symptoms resolved (with sequelae); (2) Symptoms resolved (no sequelae); (3) Symptoms persist; (4) require hospitalization; (5) require medical intervention to prevent serious outcomes; and (6) death.

9. COMPLETION OF CLINICAL TRIAL

After obtaining the immunogenicity test results 28 days after the full course of immunization, and completing the safety follow-up for no less than 1 month, and ensuring the authenticity, integrity and accuracy of clinical trial data, the database is locked and unblinded to perform safety, immunogenicity and vaccine efficacy evaluation, and blood collections for 12 months immune persistence are completed, the study site will be closed.

10. STATISTICAL CONSIDERATIONS

10.1 Sample Size Considerations

Assume 4-fold rise rate after sequential vaccination achieve 85%, 208 subjects in each arm will be required to have 80% power to conclude non-inferiority with margin of -10% and one-sided significance level of 2.5% using Miettinen & Nurminen method.

CONFIDENTIAL

Equal GMT after sequential immunization is assumed, and standard deviation of GMT after log10 transformation is considered to be 0.7. 250 subjects in each arm will be required to have 80% power to conclude non-inferiority with margin of 2/3 and one-sided significance level of 2.5%.

Considering about 15%~20% drop-out rate, 600 subjects in three sequential cohorts (1-3 months, 4-6 months and ≥6 months after 2 doses), and 1800 subjects (900 subjects in test and control groups respectively) in total are planned to enroll.

10.2 Analysis Set

Full Analysis Set (FAS): It includes all randomized participants who follow ITT principle, receive 1 dose of vaccine, and have valid immunogenicity data before the first dose.

Per-Protocol Set (PPS): It includs all participants who do not violate the inclusion criteria/exclusion criteria, receive 1 dose of vaccine, and have both valid pre- and post-vaccination immunogenicity data. PPS will be defined for immunogencity evaluations 14 and 28 days after sequential vaccination, respectively.

Immunogenicity Persistence Set (IPS): It include the participants who are collected

with blood samples at each time point for immunogencity persistence evaluation and with valid antibody data. Define the IPS for immunogenicity for 3, 6, 9, 12, 18 and 24 months after sequential immunization respectively.

Safety Set (SS): It includes all participants who receive one dose of vaccination.

In immunogencity analysis, the participant who takes the vaccine erroneously will be included as randomized, and the safety data will be analyzed as treated.

10.3 Statistical Method

10.3.1 General Considerations

Summary statistics including the number of patients, mean, standard deviation, median, minimum and maximum, will be presented for all continuous variables. For categorical variables, per category, the absolute counts (n) and percentages (%) of patients with data, and if appropriate, the number of patients with missing data, will be presented.

All statistical analysis will be performed by using SAS 9.4 or later version.

10.3.2 Subject Disposition

CONFIDENTIAL

The number of subjects who are enrolled, completed and discontinued will be summarized in each group. The reason for discontinuation will be analyzed as well.

The demographic characteristics will be summarized and described by treatment group.

10.3.3 Immunogenicity Analysis

ANCOVA model will be employed to analyze post-vacciantion GMT. In the model, log-trandformed GMT after vacciantion will be included as dependent variable, pre-vaccination GMT after log-transformation is the covariate and group assignment and sequential vaccination time (1-3 months, 4-6 months and ≥6 months after the sequential dose) are the fixed effect. The adjusted LSMean in each arm and difference between the two groups together with 95% confidence interval (CI) will be estimated from the model. After inverse log-transformation, the adjusted GMT after vaccination and GMT ratio together with 95% CI between treatment and control groups will be derived. If the lower bound of 95%CI is larger than 0.67, the non-inferiority result will be concluded.

The 4-fold rise, and the proportions of antibody tittered ≥1:16、≥1:32 and ≥1:64 after vaccination in both vaccine and placebo groups will be estimated. The corresponding 95% CIs will be derived from Clopper-Pearson method. The difference between the two groups and their 95%CIs will be estimated by CMH method including the stratification factor (sequential vaccination time). If the lower bound of 95%CI in 4-fold rise rate is larger

than -10%, the non-inferiority will be concluded.

The descriptive statistics, including geometric mean and corresponding 95% CI, are employed to summarize GMT and GMI after sequential dose. The t-tests after log transformation will be compared between the two groups.

The reverse cumulative distribution plot is to depict the pre- and post-vaccination antibody data in the vaccine and placebo groups.

10.3.4 Safety Evaluation

Adverse events and serious adverse events will be coded using the Medical Dictionary for Regulatory Activities (MedDRA), and summarized by system organ class (SOC) and preferred term (PT). AEs will be summarized by solicied and unsolicited AEs as well. The treatment emergent adverse events (TEAEs) will be analyzed here. AEs occurring before vaccination will be listed.

All TEAEs, vaccine-related TEAEs, vaccine-unrelated TEAEs, Grade≥3 TEAEs and vaccine-related Grade≥3 TEAEs will be summarized by group. The frequency, subjects and percentage in each group will be reported. Fisher exact test will be used to compare the difference between the groups. The strat time and severity of TEAEs will be summaried. The vaccine-related TEAEs and vaccine-unrelated TEAEs will be listed.

CONFIDENTIAL

All TESAE, vaccine-related TESAEs and vaccine-unrelated TESAEs will be summarized by group. The frequency, subjects and percentage in each group will be reported. Fisher exact test will be used to compare the difference between the groups. All TESAEs will be listed.

10.4 Subgroup Analysis

The immunogencity analysis will be conducted in each sequential vaccination cohort (1-3 months, 4-6 months and ≥6 months after two doses).

10.5 Interim Analysis

No interim analysis will be planned in this study.

10.6 Multiplicity

Only when both non-inferiority conclusion in 4-fold rise and GMT after vaccaition between treatment and control groups are achieved, the non-inferiority between the two groups will be concluded finally. Therefore, no multiplicity adjustment is required here.

10.7 Handling of Missing Data

In FAS immunogenicity analysis, LOCF (Last Observation Carried Forward) method will be used to impute the missing values in antibody data after vaccination. And other

immunogencity endpoints will be derived from the imputed data. The missing data in safety and immunogencity persietence evaluation will be not imputed.

11. ETHICAL AND REGULATORY MATTERS

This clinical trial will be conducted by the Sponsor, the Investigator, and delegated Investigator staff and Sub-investigator, in accordance with consensus ethics principles derived from international ethics guidelines, including the Declaration of Helsinki, and the ICH guidelines for good clinical practice (GCP), all applicable laws, rules and regulations.

11.1. Ethics Committees and Regulatory Authorities

The ICH-GCP (E6 (R2)) guidelines require that approval must be obtained from an Independent Ethics Committee (IEC) prior to participation of human subjects in research studies. Prior to the study onset, the protocol, informed consent, advertisements to be used for subject recruitment, and any other written information regarding this study to be provided to the subject or the subject's legal guardian must be approved by the IEC. Documentation of the relevant national IEC approval and of the IEC compliance with ICH Guideline E6 will be maintained by the site and will be available for review by the Sponsor or its designee or by the authorized members of regulatory agencies.

CONFIDENTIAL

The respective Ethics Committees approvals should be signed by the IEC Chairman or designee and must identify the IEC name and address, the clinical protocol by title and/or protocol number and the date approval and/or favorable opinion was granted. Also, a list of the EC members who attended the meeting when the Protocol/ Protocol amendment was discussed, including names and qualifications, needs to be provided by the EC to the investigator or the sponsor/ his representative.

If any alterations, other than changes of administrative nature only, are made to the study protocol, a formal protocol amendment will be issued and submitted to relevant IEC for approval. The amendment will not be implemented until IEC approval, except in cases where immediate implementation is necessary to eliminate or prevent imminent hazard to the subjects.

In the same way, approval from regulatory authorities (RA) should be granted before beginning the study. The investigator or the sponsor representative must provide to the regulatory authorities the name and address of the EC along with a statement from the EC that it is organized according to GCP and the applicable laws and regulations. Amendments will be submitted to RA too for approval.

11.2. Responsibilities of the Sponsor

The Sponsor of this clinical trial is responsible to regulatory authorities for taking all reasonable steps to ensure the proper conduct of the clinical trial as regards ethics, clinical trial protocol compliance, and integrity and validity of the data recorded on the e-CRFs. Thus, the main duty of the monitoring team is to help the Investigator and the Sponsor maintain a high level of ethical, scientific, technical and regulatory quality in all aspects of the clinical trial.

At regular intervals during the clinical trial, the site will be contacted, through monitoring visits, letters or telephone calls, by a representative of the monitoring team to review study progress, Investigator and subject compliance with clinical trial protocol requirements and any emergent problems. These monitoring visits will include but not be limited to review of the following aspects: subject informed consent, subject recruitment and follow-up, SAE documentation and reporting, AE documentation, IP allocation, subject compliance with the instructions, IP accountability, concomitant therapy use, and quality of data.

CONFIDENTIAL

11.3. Responsibilities of the Investigator

The Investigator is responsible for ensuring that the clinical study is performed in accordance with the protocol, the ethical principles that have their origin in the Declaration of Helsinki (version as of October 2013) as well as with the ICH Note for Guidance on Good Clinical Practice (ICH, Topic E6, R2, 2016), relevant site SOPs and applicable regulatory requirements. These documents state that the informed consent of subjects is an essential precondition for participation in the clinical study.

The Investigator is required to ensure compliance with all procedures required by the clinical trial protocol and with all study procedures provided by the Sponsor (including security rules). The Investigator agrees to provide reliable data and all information requested by the clinical trial protocol (with the help of the e-CRF, Discrepancy Resolution Form [DRF] or other appropriate instrument) in an accurate and legible manner according to the instructions provided and to ensure direct access to source documents by Sponsor representatives.

If any circuit includes transfer of data particular attention should be paid to the confidentiality of the subject's data to be transferred.

The Investigator may appoint such other individuals as he/she may deem appropriate as Sub-investigators to assist in the conduct of the clinical trial in accordance with the clinical trial protocol. All Sub-investigators shall be appointed and listed in a timely manner. The Sub-investigators will be supervised by and work under the responsibility of the Investigator. The Investigator will provide them with a copy of the clinical trial protocol and all necessary information.

In 1998, the US Food and Drug Administration (FDA) introduced a regulation (21 CFR, Part 54) entitled "Financial Disclosure by Clinical Investigators." For studies conducted in any country that could result in a product submission to the FDA for marketing approval and which contribute significantly to the demonstration of efficacy and safety of the drug (named "covered studies" by the FDA), the Investigator and all sub-Investigators are obliged to disclose their financial interest which they or their spouses and dependent children may have in the Sponsor. This information is required during the study and until 12 months after its completion.

11.4. Subject Information and Consent

CONFIDENTIAL

A prerequisite for a subject participating in the study is his/her or their legal representative or parent's/guardian's written informed consent. Adequate information must therefore be given to the subject by the Investigator/ designated personnel before informed consent is obtained. One or more Informed Consent Forms in the local language and prepared in accordance with the Note for Guidance on Good Clinical Practice (ICH, Topic E6 (R2)) will be provided by the Sponsor for the purpose of obtaining informed consent. In addition to this written information, the Investigator or his designate will inform the subject verbally. In doing so, the wording used will be chosen so that the information can be fully and readily understood by laypersons. The Informed Consent Form will be revised whenever important new information becomes available that may be relevant to the consent of subjects.

The subject must be informed that his/her personal trial-related data will be used by CNBG and his subsidiaries (sponsor) in accordance with the local data protection law. The level of disclosure must also be explained to the subject.

The subject must be informed that his/ her medical records may be examined by authorized monitors or Clinical Quality Assurance auditors appointed by CNBG and his subsidiaries (sponsor), by appropriate IRB / IEC members, and by inspectors from

regulatory authorities.

The Informed Consent Form must also be signed and personally dated by the subject and legal representative or parents/guardian and by the Investigator/person designated by the Investigator to conduct the informed consent discussion. Provision of consent will be confirmed in the eCRF by the Investigator. The signed and dated declaration of informed consent will remain at the Investigator's site and must be safely archived by the Investigator so that the forms can be retrieved at any time for monitoring, auditing and inspection purposes. A copy of the signed and dated information and consent should be provided to the subject prior to participation.

11.5. Compensation to Subjects

Appropriate insurance coverage is provided by China National Biotec Group (CNBG) in line with legal requirements and GCP guidance. Details can be asked at the investigator's site (certificates and conditions in the Investigator Site File).

Insurance coverage will be provided by a local insurance provider.

11.6. Subject Confidentiality

CONFIDENTIAL

The investigator(s) will respect and protect the confidentiality of the subject in all possible ways. Subject identification, other than subject number, initials and date of birth, will not appear in any Case Report Form (eCRF) pages or other documents given to the Sponsor. Only the investigator and the persons authorized to verify the quality and integrity of the study will have access to subject records where the subject can be identified.

Ensure that the personal secrets of the subjects will not be disclosed under the conditions of testing, biological sample collection, reporting and publication, etc. Only the subject code, blood sample number, blood collection time and test index are recorded in the blood sample. It is strictly limited to the core testing personnel to obtain electronic or written copies.

11.7. Amendment to Subject Related Information

Should a Protocol amendment become necessary, the subject information and consent form may need to be revised to reflect the changes to the Protocol. It is the responsibility of the investigator to ensure that an amended consent form is reviewed and has received approval/ favorable opinion from the IRB / IEC and CA and/or the regulatory authority has provided approval / has been notified (depending on local laws and regulations), and that it is signed by all subjects subsequently entered in the trial and those currently in the trial, if affected by the amendment.

11.8. Direct Access to Source Documentation

Source data are all the information in original records and certified copies of original records of clinical findings, observations, or other activities in the study, which are necessary for the reconstruction and evaluation of the study. Source data are contained in source documents (originals or certified copies).

Source Documents are original documents, data, and records (e.g., clinical and office charts, laboratory notes, memoranda, subjects' diaries, pharmacy dispensing records, recorded data from automated instruments, copies or transcriptions certified after verification as being accurate and complete, subject files, and pharmacy records or prescriptions, laboratory reports or, computer printouts from the laboratory websites, vaccine handover sheet and delivery notes). All of them are expected to be reviewed signed, dated and assessed by investigator. The source documents must contain study participation information.

It is the Investigator’s obligation to collect and present of all relevant medical data in the subject’s medical file. Sponsor name and trial number, subject identification (name, date of birth, address, etc.), information that the informed consent was obtained prior any screening procedures, visit dates, subject number, vaccine information, efficacy data, safety data, concomitant medications and date and reason of completion of the study.

Source records should be preserved for the maximum period of time required by local requirements.

All information recorded on the eCRFs for this study must be consistent with the subject’s source documentation.

Besides the monitor of the Contract Research Organization (CRO), regulatory authorities, members of ethics committees and the Sponsor's clinical quality assurance group or any other Sponsor’s representative, may carry out source data checks and/or on-site audits or inspections. Direct access to source data will be required for these audits and inspections; they will be carried out giving due consideration to data protection and medical confidentiality. The investigator will assure the CRO and the sponsor of the necessary support at all times.

12. STUDY MANAGEMENT

12.1. Case Report Form (eCRF) Handling

The data recorded during the course of this study will be documented in the form of eCRF, and must be forwarded to the sponsor or appointed designee. Then they should be processed, evaluated and stored in anonymous form in accordance with data protection regulations.

The investigator must ensure that the eCRFs and any other associated documents contain no mention of any subject names and other privacy information. The eCRFs must be completely filled in. They are regulatory documents and must be suitable for submission to authorities.

All data in the eCRFs must be derived from source documentations. Clinical data will be captured via local medical system, the data will be transmitted to CTMS and generate eCRF. The investigator site staff will enter and edit the data via a secure network, with secure access features (username, password and secure identification or username and password – an electronic password system). A complete electronic audit trail will be maintained. The investigator will approve the data using an electronic signature (Ref: 21 CFR Part 11), and this approval is used to confirm the accuracy of the data recorded. The electronic CRFs (eCRFs) will be used, the investigator's data will be accessible from the investigator's site throughout the trial. Relevant medical history prior to enrolment will be documented at the baseline visit. Thereafter during the trial, narrative statements relative to the subject's progress during the trial will be maintained. The electronic CRFs must be kept current to reflect subject status at each phase during the course of the trial. The subjects must not be identified on the electronic CRF by name. Appropriate coded identification (i.e. Subject Number) must be used. The investigator must make a separate confidential record of these details (subject identification code list) to permit identification of all subjects enrolled in a clinical trial in case follow-up is required. While a trial is ongoing and until the access to the database has been terminated, there will be no Documentation of Changes (DOCs).

12.2. Source Data and Subject Files

Source documents provide evidence for the existence of the subject and substantiate the integrity of the data collected. Source documents are filed at the investigator's site.

The investigator has to keep a written or electronic subject file for every subject

participating in the clinical study. In this subject file, the available demographic and medical information of a subject has to be documented, in particular the following: name, date of birth, sex, height, weight, statement of entry into the study, study identification, randomization number, the date of informed consent, all study visit dates, predefined performed examinations and clinical findings, observed AEs, and reason for withdrawal from the study, if applicable.

It should be possible to verify the inclusion and exclusion criteria defined in the protocol from the available data in this file.

It must be possible to identify each subject by using this subject file. Additionally, any other documents with source data, especially original printouts of data that were generated by technical equipment have to be filed. This includes but not limited to ECG tracings, X-ray films, CT and MRI scans, laboratory value listings and QoL questionnaires, etc. All these documents have to bear at least the subject identifier and the printing date printed by the recording device to indicate to which subject and to which study procedure the document belongs. The medical evaluation of such records should be documented as necessary and signed/dated by the investigator.

CONFIDENTIAL

Computerized subject files will be printed whenever source data verification is performed by the monitor. Printouts must be signed and dated by the investigator, countersigned by the monitor and kept in a safe place.

Data entered in the eCRFs that are transcribed from source documents must be consistent with the source documents or the discrepancies must be explained. The investigator may need to request previous medical records or transfer records, depending on the case; also, current medical records must be available.

Data on the subject dairies are considered source data and have to be stored along with the subject file. The data of the dairies will be forwarded to the sponsor or a CRO appointed by the sponsor for data entry into the data base.

12.3. Investigator Site File and Archiving

The Investigator will be provided with an Investigator Site File at the start of the study. This file contains all relevant documents necessary for the conduct of the study. This file must be safely archived after termination of the study. It is the responsibility of the Investigator to ensure that the files are stored for at least 15 years beyond the end of the clinical study. All original subject files must be stored for the longest possible time permitted by the regulations at the hospital, research institute, or practice in question. If archiving can no longer be maintained at the site, the Investigator should notify the Sponsor.

12.4. Monitoring, Quality Assurance and Inspection by Authorities

This study is to be conducted in accordance with the ICH Note for Guidance on Good Clinical Practice (ICH, Topic E6 (R2), 2016). The appointed clinical monitor will arrange regular visits to the study center(s) to check progress with the study and to check eCRF completion.

During monitoring visits, the monitors will:

- Help resolve any problems;
- Examine eCRF for omission of data, compliance and possible AEs;
- Discuss inconsistencies in the study data;
- Ensure that all study materials are correctly stored and dispensed;
- Check adherence to the obligations of the investigator;
- Review consent forms, in particular the date of consent and signature;
- Perform source data verification as described below.

In line with International Conference on Harmonization (ICH)-Good Clinical Practice (GCP) guidelines, monitoring will include verification of data entered in the eCRF against original subject records. This verification will be performed by direct access to the original subject records, and the Sponsor guarantees that subject confidentiality will be respected at all times. Participation in this study will be taken as agreement to permit direct source data verification.

CONFIDENTIAL

The investigator / institution will permit trial-related monitoring, audits, IRB / IEC review and regulatory inspection, providing direct access to all related source data / documents. eCRFs and all source documents, including progress notes and copies of laboratory and medical test results must be available at all times for review by the sponsor’s clinical trial monitor or a CRO appointed by the sponsor, auditor and inspection by health authorities (e.g. FDA). The Clinical Research Associate (CRA) / on site monitor and auditor may review all CRFs/eCRFs, and written informed consents.

12.5. Changes to the Study Protocol

Changes to, or formal clarifications of, the study protocol must be documented in writing.

Major changes to the protocol will be described in a "Protocol Amendment". It will be submitted to the relevant Ethics Committee(s)/Institutional Review Board(s) and to authorities, where required. Approval/favorable opinion from the relevant Ethics Committee(s)/Institutional Review Board(s) will be required prior to implementation of the amendment.

Any amendment affecting the subject requires the subject's informed consent prior to implementation.

Changes of administrative or technical nature will be recorded in a document entitled "Administrative Change to Study Protocol". It will be sent for information to the relevant Ethics Committee(s)/ Institutional Review Board(s) or to authorities, if so required. Amendments and administrative changes will be signed by all signatories of the protocol.

All Investigators will acknowledge the receipt and confirm by their signature on the Amendment or Administrative Change Signature Sheet that they will adhere to the Amendment/Administrative Change. This sheet will be issued in duplicate and after signing, one will be filed in the Investigator Site File and one in the Study Master File.

12.6. Study Report and Publication Policy

After conclusion of the study, an integrated clinical and statistical study report shall be written by the Sponsor.

The respective EC and competent authority need to be notified about the end of the trial (last subject/subject out) or early termination of the trial.

China National Biotec Group (CNBG) is as much as possible dedicated to support process of free exchange of relevant scientific information. Any publication of the result of this trial must be consistent with the CNBG publication policy. The rights of the investigator and of the sponsor with regard to publication of the results of this trial are described in the investigator contract. As a general rule, no trial results should be published prior to finalization of the Clinical Trial Report (CTR).

CONFIDENTIAL

The present trial will be published in a clinical trial registry indicating the trial dates and indication as well as the number of sites and location. The subject identity should be kept confidential.

13. REFERENCES

[1] Wu A, Peng Y, Huang B, Ding X, Wang X, Niu P, Meng J, Zhu Z, Zhang Z, Wang J et al: Genome Composition and Divergence of the Novel Coronavirus (2019-nCoV) Originating in China. Cell Host Microbe 2020.

[2] Chan JF, Yuan S, Kok KH, To KK, Chu H, Yang J, Xing F, Liu J, Yip CC, Poon RW et al: A familial cluster of pneumonia associated with the 2019 novel coronavirus indicating person-to-person transmission: a study of a family cluster. Lancet 2020, 395(10223):514-523.

[3] Li Q, Guan X, Wu P, Wang X, Zhou L, Tong Y, Ren R, Leung KSM, Lau EHY, Wong JY et al: Early Transmission Dynamics in Wuhan, China, of Novel Coronavirus-Infected Pneumonia. N Engl J Med 2020.

[4] Wrapp D, Wang N, Corbett KS, Goldsmith JA, Hsieh CL, Abiona O, Graham BS, McLellan JS: Cryo-EM structure of the 2019-nCoV spike in the prefusion conformation. Science 2020.

[5] General Office of the National Health Council: SARS-CoV-2 Pneumonia Prevention and Control Program (7th Edition). 2020.

CONFIDENTIAL

[6] Ababneh M, Alrwashdeh M, Khalifeh M: Recombinant adenoviral vaccine encoding the spike 1 subunit of the Middle East Respiratory Syndrome Coronavirus elicits strong humoral and cellular immune responses in mice. Vet World 2019, 12(10):1554-1562.

[7] "Guiding Principles for Quality Management of Vaccine Clinical Trials (Tentative)", National Medical Products Administration, Food and Drug Administration, Pharmaceutical Administration [2013] No.228, October 31, 2013

[8] "Regulations on the Administration of Qualification Accreditation of One-time Vaccine Clinical Trial Site", National Medical Products Administration, Food and Drug Administration, Pharmaceuticals Administration [2013] No.248, December 10, 2013

[9] Provisions on the Administration of Reporting Serious Adverse Events in Vaccine Clinical Trials (Tentative), National Medical Products Administration, No.6 [2014] of the Food and Drug Administration, January 17, 2014

[10] Good Clinical Practice (GCP), National Medical Products Administration, 2003

[11] Measures for Identification of Abnormal Reactions to Vaccination: Ministry of Health, Ministry of Health Decree No.60, July 17, 2008

[12] Standards and Procedures for Rapid Reporting of Safety Data during Drug Clinical Trials, Drug Evaluation Center, April 27, 2018

[13] "Guidelines for Classification Standards of Adverse Events in Clinical Trials of Prophylactic Vaccines" Circular No.102 of 2019

[14] FDA Development and Licensure of Vaccines to Prevent COVID-19, Guidance for Industry, June 2020

[15] China Diagnosis and Treatment Protocol for Novel Coronavirus Pneumonia (Trial Version 8)”

[16] UAE National Guidelines for Clinical Management and Treatment of COVID-19, July 15, 2020 (Version 4.3) (UAE)

[17] Bahrain COVID-19 National Protocols, Oct 11, 2020

[18] COVID-19 Treatment Guidelines Panel. Coronavirus Disease 2019 (COVID-19) Treatment Guidelines. National Institutes of Health.

[19] Al Kaabi N, Zhang Y, Xia S. Effect of 2 Inactivated SARS-CoV-2 Vaccines on Symptomatic COVID-19 Infection in Adults: A Randomized Clinical Trial. JAMA. 2021 May 26. doi: 10.1001/jama.2021.8565. Epub ahead of print. PMID: 34037666.

[20] Blackwelder WC. "Proving the Null Hypothesis" in Clinical Trials. Control. Clin.

[21] Julious SA. Sample sizes for clinical trials with Normal data. Statist. Med. 2004; 23:1921-1986.

CONFIDENTIAL

Appendix

Case Definition

Suspected cases:

Comprehensive judgment based on epidemiological history and clinical symptoms:

Have any of the epidemiological history, and have two or more A symptoms, or have one or more B symptoms; or

with imaging features of COVID-19

If there is no clear epidemiological history, they should have two or more A symptoms or one or more B symptoms and detectable SARS-CoV-2 specific IgM; or have two or more A symptoms and One or more B symptoms; with imaging features of COVID-19

① Epidemiological history

A. Long-term residence or stay in the affected area for more than 7 days is deemed to have an epidemiological history; or

CONFIDENTIAL

B. History of travel or residence in the community where the case was reported within 14 days before the onset of illness; or

C. In contact with SARS-CoV-2 infected or asymptomatic infected persons within 14 days before the onset; or

D. Cluster cases (2 or more cases of fever and/or respiratory symptoms occurred in a small area such as home, office, school, etc. within 2 weeks).

② Clinical symptoms

Symptoms A (last for at least 2 days): fever (axillary temperature ≥37.5℃); chills; sore throat; fatigue; nasal congestion or runny nose; body pain, muscle pain; headache; nausea or vomiting; diarrhea.

Symptoms B: Cough (last for at least 2 days); newly developed taste or smell disorders (last for at least 2 days); shortness of breath or difficulty breathing;

③ Imaging features of COVID-19

Confirmed cases:

On the basis of the determination of the suspected case, there’s also a COVID-19 PCR

test result.

Differential diagnosis:

Encourage any possible pathogenic differential diagnosis.

Clinical Classifications of Confirmed COVID-19 Cases:

Mild

The clinical symptoms are mild, and there is no imaging characteristics of pneumonia.

Moderate

Showing fever, respiratory symptoms, and imaging characteristics of pneumonia.

Severe

Meet any of the following criteria:

1. Respiratory distress (RR≥30 breaths/min);
2. Oxygen saturation≤93% at rest;
3. Arterial partial pressure of oxygen (PaO_2)/ fraction of inspired oxygen (FiO_2)≦300mmHg (1mmHg=0.133kPa);
4. The clinical symptoms progressively worsened, and the chest imaging showed >50% obvious lesion progression within 24-48 hours.

CONFIDENTIAL

Critical

Meet one of the following criteria:

1. Respiratory failure and requiring mechanical ventilation;
2. Shock;
3. With other organ failure that requires ICU care;
4. Death
